# Bis-α,ω-bisacylphosphane oxides: simple access to crosslinked polymers with tunable properties

**DOI:** 10.1039/d6ta01719c

**Published:** 2026-05-26

**Authors:** Renata Raptova, Tanja Wiesner, Daniel Griess, Julian Maier, Roland C. Fischer, Anne-Marie Kelterer, Mercedes Linares-Moreau, Christoph Walkner, Thomas Griesser, Mathias Wiech, Georg Gescheidt, Michael Haas

**Affiliations:** a Institute of Inorganic Chemistry, Graz University of Technology Stremayrgasse 9/IV 8010 Graz Austria michael.haas@tugraz.at; b Christian Doppler Laboratory for New Semiconductor Materials based on Functionalized Hydrosilanes Stremayrgasse 9/IV 8010 Graz Austria; c Institute of Physical and Theoretical Chemistry, Graz University of Technology Stremayrgasse 9/II 8010 Graz Austria; d Chair of Chemistry of Polymeric Materials, Technical University of Leoben Otto-Glöckel-Strasse 2 8700 Leoben Austria; e Chair of General and Analytical Chemistry, Technical University of Leoben Franz-Josef-Strasse 18 8700 Leoben Austria

## Abstract

We present an efficient one-pot synthesis of bis-α,ω-bisacylphosphine oxides (Bis-BAPOs) obtained by coupling α,ω-dibromoalkanes with sodium bis(mesitoyl)-phosphide. The resulting tetrafunctional photoinitiators display absorption characteristics similar to the parent BAPO, yet allow pairwise, wavelength-selective activation. Stepwise irradiation at *λ* = 450 nm and *λ* = 385 nm triggers consecutive α-cleavage of the BAPO and the subsequently generated monoacylphosphane oxide (MAPO) moieties, enabling controlled polymer growth and branching. This strategy enables the preparation of hydrophilic, lipophilic, and amphiphilic polymer materials from standard monomers. Atomic force microscopy and contact-angle measurements reveal pronounced differences in surface chemistry despite homogeneous film morphologies, highlighting the impact of initiator architecture on interfacial properties. Migration analyses and photo-DSC experiments demonstrate that the molecular structure of the photoinitiator governs curing behaviour, conversion kinetics, and extractability, underscoring the potential of bis-BAPOs as tunable components for advanced photopolymer materials.

## Abstract 2

We present an efficient one-pot synthesis of bis-α,ω-bisacylphosphine oxides (Bis-BAPOs) obtained by coupling α,ω-dibromoalkanes with sodium bis(mesitoyl)-phosphide.

## Graphical abstract

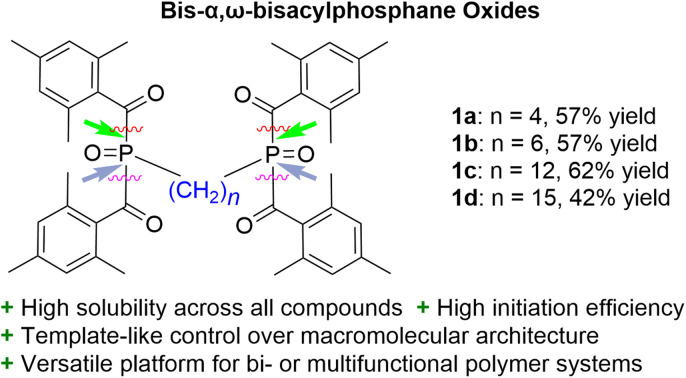

## Introduction

**Fig. 1:**
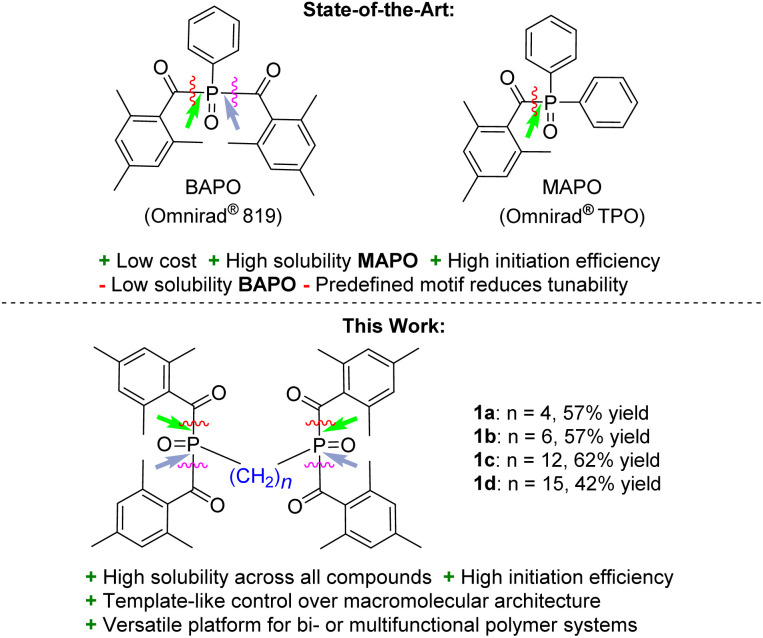
State of the Art P-based PIs (top). Bis-α,ω-bisacylphosphane oxides (bottom, this work).

The demand for tunable and sustainable materials with industrial relevance has driven innovation. Here, UV/Vis-curable systems have been utilized in many applications, ranging from coatings to 3D printing.^1^ Photoinitiators are essential components of such formulations because they direct the course of polymerizations allowing temporal and spatial control.^2^ Radical polymerization offers the use of a wide variety of monomers. However, a standard procedure involving photolysis and the use of monofunctional monomers is not suited to provide polymers with specific interfacial properties, *e.g.*, hydrophobic/hydrophilic or self-healing domains.^3^ It has been shown that bisacylphosphane oxide-based (BAPO) initiators act as selective two-phase reagents.^4,5^ A primary irradiation of BAPOs (450–500 nm) in the presence of monomers produces a polymer with a monoacylphosphane end group (MAPO) (see Fig. 1, state-of-the-art). This latter end group again acts as a photoinitiator when irradiated at a wavelength below 430 nm. Accordingly, polymers with two blocks can be produced in a convenient way.^4,6^ More recently, acylgermanes have emerged as an attractive alternative class of visible-light photoinitiators, combining strong absorption in the near-UV/visible region with high initiation efficiency and reduced oxygen sensitivity.^7^ Crosslinking is of advantage because it enhances material strength. Using a photoinitiator, which predefines the nodes in a crosslinked polymer in a controlled way, offers advantages over the use of polyfunctional monomers.

The aim of our work is to indicate a straightforward access to BAPO-based photoinitiations, which can be successively and selectively activated at appropriate wavelengths and, at the same time, act as nodal points in crosslinked polymers. We evaluate their potential application as decisive reagents for tailor-made bifunctional polymers (see Fig. 1, this work).

## Results and discussion

### Synthesis of Bis-BAPOs

**Scheme 1:**
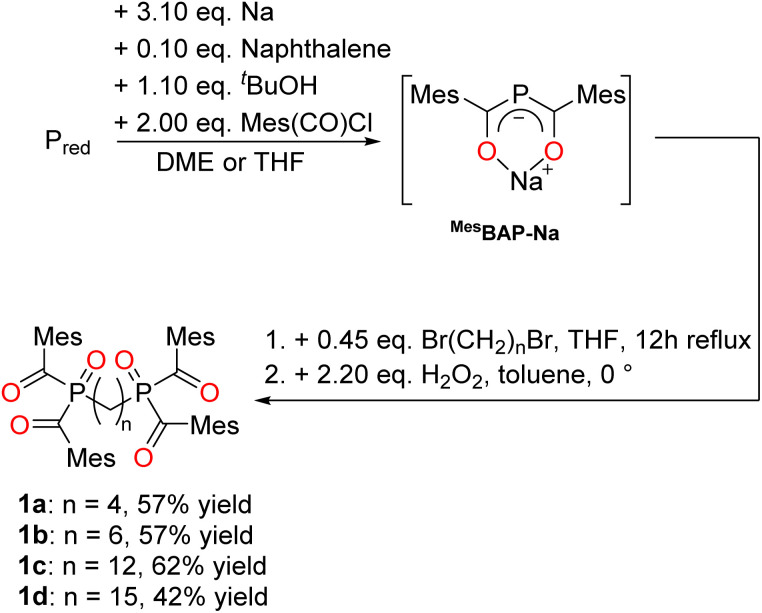
Synthetic procedure towards bis-α,ω-bisacylphosphane oxides (Bis-BAPOs) 1a–d.

We decided to use the sodium bis(mesitoyl)-phosphide MesBAP-Na as our building block, as it is also the main intermediate for the BAPO synthesis.^8^ Consequently, this phosphide was reacted without prior isolation with 0.45 equivalents of corresponding dibromoalkanes (1,4-dibromobutane, 1,6-dibromohexane, 1,12-dibromododecane and 1,15-dibromopentadecane) and refluxed for 12 hours. *In situ* oxidation, aqueous work-up and column chromatography gave the products 1a–d in moderate to good yields of 42–62% (compare Scheme 1). Compounds 1a–d exhibit high stability under ambient conditions and are not sensitive to air or moisture. No decomposition or significant changes in their spectroscopic properties were observed even after prolonged exposure to atmospheric humidity.

NMR spectra and detailed assignments are provided in the Experimental section and in the SI. The observed ^13^C-NMR shifts of the carbonyl C atoms of all isolated Bis-BAPOs were found in the region between *δ* = 216.4 and *δ* = 217.5 ppm, which is typical for carbonyl groups attached to the P=O moiety. In addition, the ^31^P-NMR shifts of the phosphorous atoms were found in the region between *δ* = 26.3 and *δ* = 28.6 ppm. This is in line with related photoinitiators.^8,9^

### X-ray crystallography

**Fig. 2:**
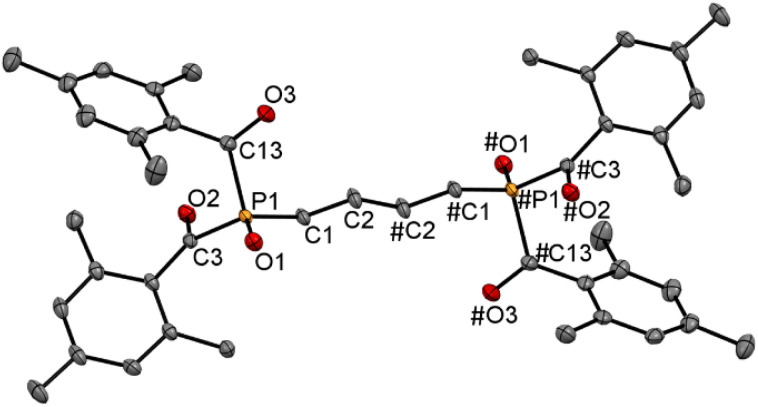
ORTEP representation of compound 1a. Thermal ellipsoids are drawn at the 50% probability level. Hydrogen-atoms are omitted for clarity. Selected bond lengths [Å] and angles [°] with estimated standard deviations: P(1)–O(1) 1.4823(11), P(1)–C(1) 1.8053(13), P(1)–C(3) 1.8803(14), O(2)–C(3) 1.2165(17), O(3)–C(13) 1.2124(18), C(1)–C(2) 1.5330(19), C(1)–P(1)–C(3) 102.82(6), O(1)–P(1)–C(1) 115.41(7), O(2)–C(3)–P(1) 111.71(10).

**Fig. 3:**
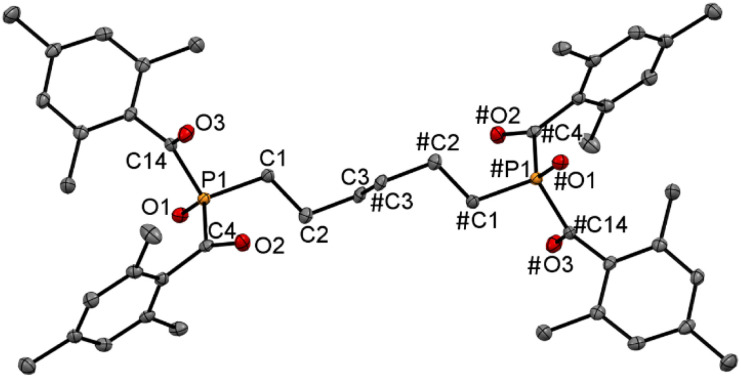
ORTEP representation of compound 1b. Thermal ellipsoids are drawn at the 50% probability level. Hydrogen atoms are omitted for clarity. Selected bond lengths [Å] and angles [°] with estimated standard deviations: P(1)–O(1) 1.478(12), P(1)–C(1) 1.795(16), P(1)–C(4) 1.893(16), O(2)–C(4) 1.212(19), O(3)–C(14) 1.219(2), C(1)–C(2) 1.529(2), C(1)–P(1)–C(4) 102.78(7), O(1)–P(1)–C(1) 117.60(7), O(2)–C(4)–P(1) 116.40(12).

Crystals suitable for single-crystal XRD were obtained for compounds 1a and 1b. These crystal structures are described in the following section. Both single crystals could be grown by cooling concentrated solutions in diethyl ether to −30 °C. All compounds show the expected bond lengths and angles. The molecular structures are depicted in Fig. 2 and 3. More information about the crystal structures is provided in the SI (Table S9).

### Optical properties and reactivity

**Fig. 4:**
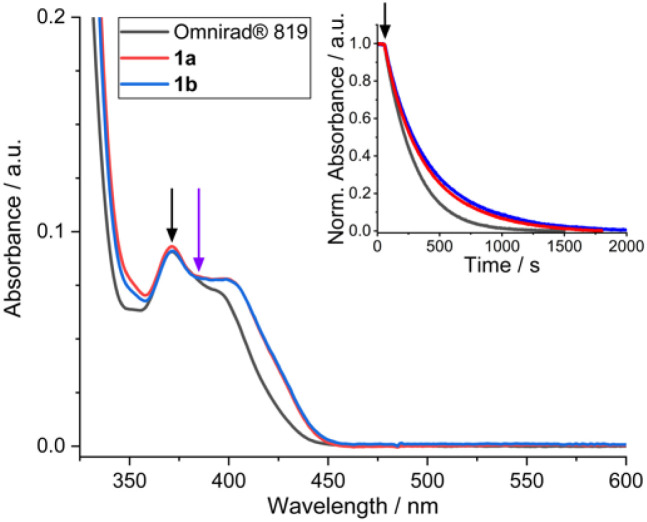
UV/Vis spectra of 1a, 1b, and Omnirad 819 in THF (10^−4^ mol l^−1^). The insert shows the decay of the absorbance at 371 nm (black arrow) upon irradiation with a 385 nm LED (purple arrow, also used for the determination of the corresponding quantum yield, see SI).

The UV/Vis spectra of Bis-BAPOs 1a–d exhibit very similar characteristics (Fig. 4). The absorptions resemble those of BAPO^10,11^ (Omnirad 819, phenylbis(2,4,6-trimethylbenzoyl)-phosphine oxide). They display two distinct absorption maxima: a π–π* transition at 320 nm and a broad n–π* transition reaching from approx. 370 to 450 nm. Excitation of the n–π* transition induces α-cleavage at the P(O)–C(O) bond. The UV spectrum of 1a and 1b was simulated using Density Functional Theory for the more stable all-trans forms. Two long-wavelength bands can be assigned, which include all n–π* excitations. S_1_ and S_2_ are characterized as local n–π* excitations on either side of the chain forming the long-wavelength part of the band at 400 nm, whereas S_3_ and S_4_ consist of n–π* transitions at both sides of the chain leading to the shorter-wavelength part of the band at around 370 nm. The relevant excitation energies, orbitals and transition densities are collected in the SI Table S4.

Based on these results, initiator 1b was selected as a representative model compound for the polymer synthesis studies. This choice was made for practical reasons (availability, handling, and well-balanced solubility), while still being fully representative of the whole series.

**Scheme 2:**
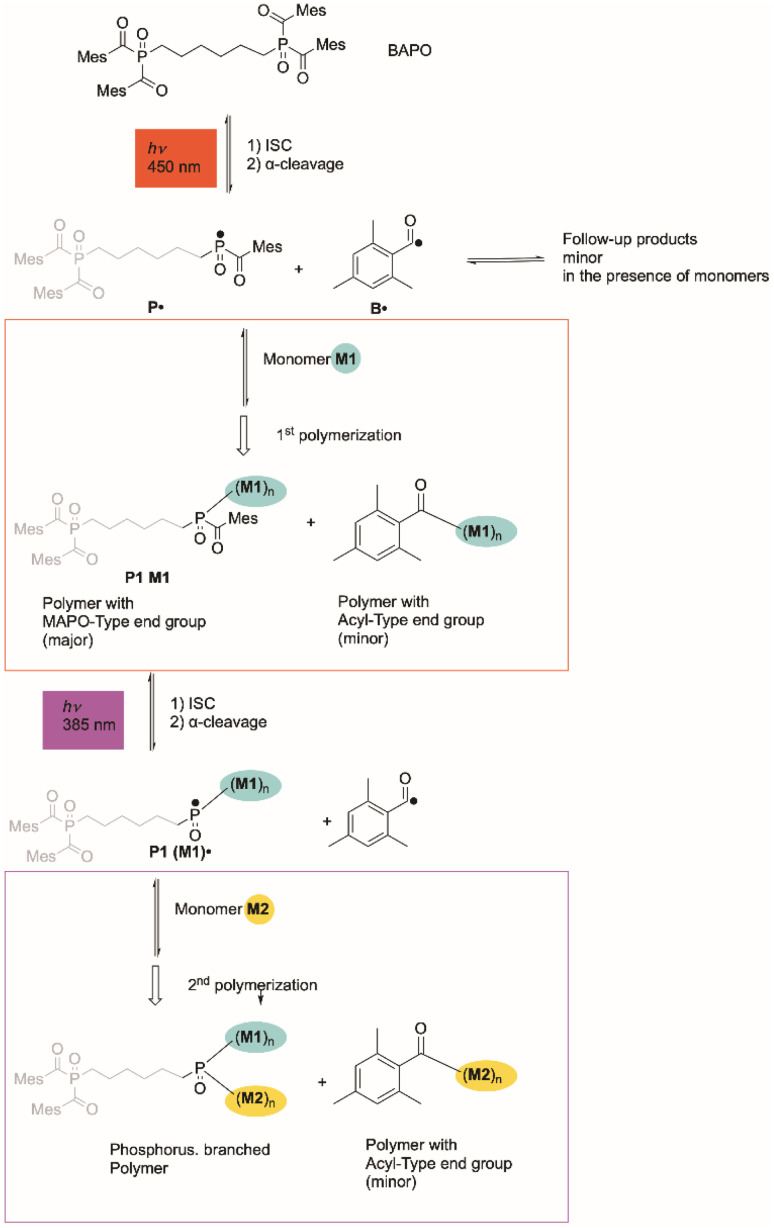
Consecutive and wavelength-dependent photo-induced reactions of 1b in the presence of monomers M1 and/or M2. For simplicity only one of the BAPO moieties is highlighted in the graph. Note that the reactions of the photoinitiating moieties happen randomly on both sides of 1b. Monomers M1 and M2 are interchangeable. Direct follow-up products of P˙ and B˙ are presented in the SI. Based on the rapid kinetics for the addition of P˙ and B˙ to the monomer double bonds, these latter products are formed at negligible yield.

Accordingly, irradiation at 405 nm (LED) results in the formation of phosphonyl (P˙) and mesitoyl radicals (B˙) (Scheme 2).

**Fig. 5:**
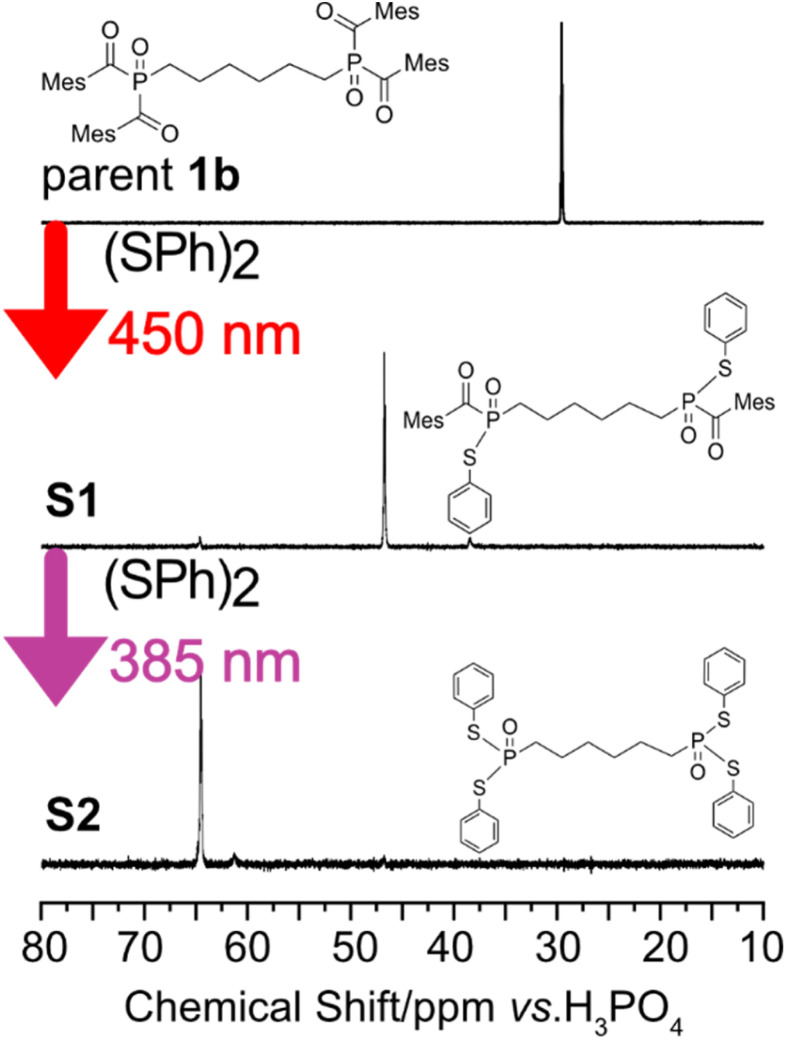
^31^P-NMR spectra of 1b in the presence of diphenyl disulfide ((PhS)_2_). Experimental conditions: 30 mM photoinitiator Bis-BAPO, 7 equiv. (PhS)_2_ in CDCl_3_, (1) irradiation step: 10 min at 450 nm LED (*in situ*), (2) irradiation step: 10 min at 385 nm LED (*ex situ*).

When the radical P˙ undergoes a follow-up reaction and becomes covalently bound to a monomer, forming a P1(M1 or M2) moiety, P1 represents a MAPO.^10^ This species is again photo-active, but at a lower wavelength (band 385–400 nm).^4,6^ Consequently, irradiation with 385 nm produces the secondary initiating radical ˙P1(M1 or M2). To confirm this selective stepwise α-cleavage, we have conducted trapping experiments using 1b and diphenyl disulfide (Ph_2_S_2_).^12^ Upon initial irradiation at 450 nm, the formation of *S*-phenyl(2,4,6-trimethylbenzoyl)phosphino-thioate (S1) becomes evident by a ^31^P NMR resonance at *δ* = 47 ppm (*vs. δ* = 26 ppm for parent 1b). Subsequent irradiation at 385 nm resulted in a ^31^P NMR signal at *δ* = 65 ppm indicating selective formation of *S*,*S*-diphenyl phosphonodithioate (S2, Fig. 5). These experiments reveal the wavelength-dependent reactivity of 1b in terms of the formation of phosphorus-centred radicals. Analogously performed spin-trap experiments substantiate these findings (see SI).

We have translated the conceptual two-wavelength experiments described above to produce branched polymers. The first step is the photolysis of 1b using an LED emitting at 450 nm leading to the α-cleavage of the BAPO moiety. In the presence of a monomer M1 the corresponding polymer is formed (P1M1). It carries a MAPO-type end group (polymer–P(O)–C(O)–Mes (Mes = mesitoyl)). MAPOs reveal a blue-shifted n–π* band at 300–440 nm. Accordingly, after the first polymerization has been accomplished irradiation at 385 nm leads to a second α-cleavage of the MAPO-type end group (Scheme 2). The presence of a monomer (either M1 or a different monomer M2) leads to a branched polymer. At 385 nm, the quantum yields *Φ* for 1a and 1b are 42 ± 4% and 34 ± 4%, respectively. Parent BAPO (Omnirad® 819) gave *Φ* = 49 ± 2% under the same conditions.

**Scheme 3:**
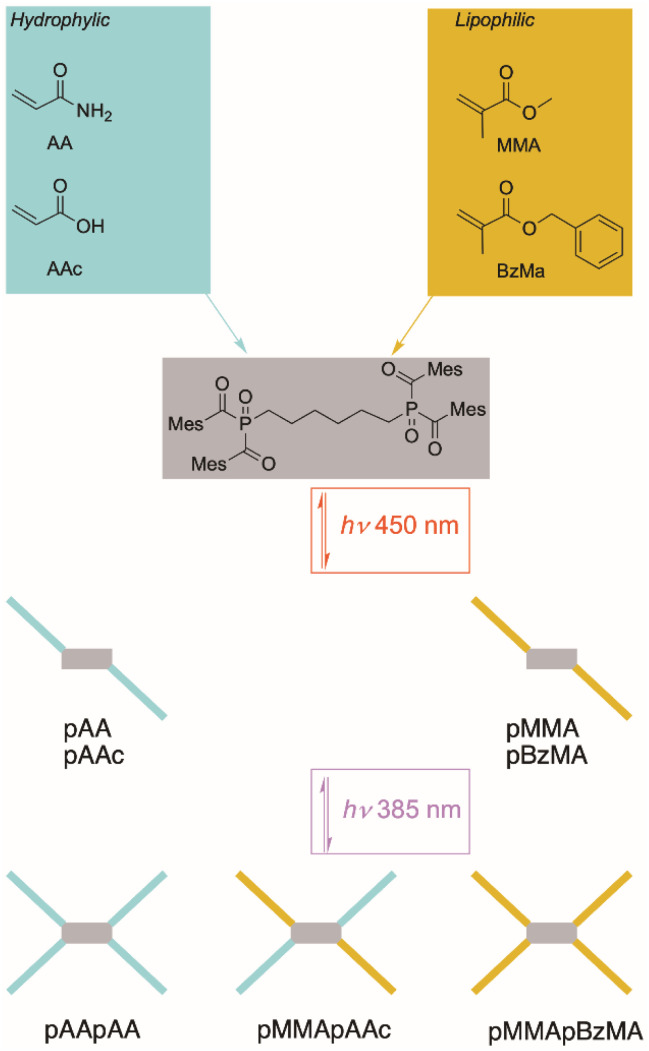
Combinations of hydrophilic and lipophilic monomers used for wavelength-dependent polymerizations with 1b as photoinitiator. For simplicity the products are colour coded to indicate the character of the polymers and the products formed by polymerization with the mesitoyl radicals (B˙) are omitted. This is justified because it has been well established, that the addition of the P-centred radicals to double bonds overrules that of B˙-type radicals by at least one order of magnitude.

To indicate the scope of the materials available by using initiators of type 1a–d, we have used hydrophilic acrylamide (AA) and acrylic acid (AAc) and lipophilic methyl methacrylate (MMA) and benzyl methacrylate (BzMA) as monomers. In a first step, by irradiating at 450 nm, we have synthesized branched homopolymers with either hydro- (pAA) or lipophilic (pMMA) monomers. After the isolation of the primary polymers, the second step (irradiation at 378 nm) was performed leading to either hydrophilic (pAApAA, pAApAc), lipophilic (pMMApMMA, pMMApBz), or amphiphilic polymers (pMMApAA, pMMApAc, Scheme 3). The polymerization processes were followed and established by IR and NMR spectroscopy (see SI).

**Fig. 6:**
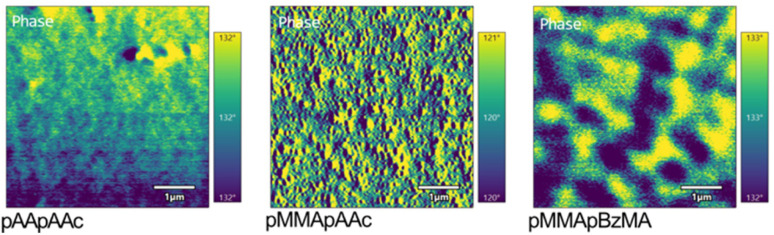
AFM topography of polymeric films pAApAA, pMMApAAc, and pMMApBzMA.

We have characterized the surfaces of the corresponding polymer films using Atomic Force Microscopy (AFM). Fig. 6 indicates the AFM images of pAApAA and pMMApBzMA with a hydrophilic and lipophilic, respectively, and pMMApAAc representing an amphiphilic surface. For all three samples, the surface morphology is flat and homogeneous with average RMS roughness below 1 nm (see SI also for all remaining samples). The pMMApBz sample shows some degree of phase separation, distinguished by domains of approx. 300–500 nm, which exhibit a clear contrast both in topography and in the phase image.

**Fig. 7:**
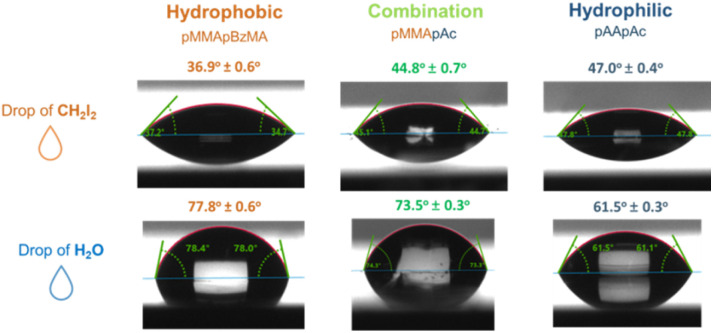
Wettability of pAApAA, pMMApAAc, and pMMApBzMA surfaces for H_2_O and CH_2_I_2_ determined by contact-angle measurements (for details, see SI).

The AFM images indicate that the surfaces are rather smooth, nevertheless, they reveal clearly distinct properties. This becomes apparent when the wettability of the covalently fused derivatives pAApAA, pMMApAAc, and pMMApBzMA is characterized by contact-angle measurements. With H_2_O, the contact angle is highest for lipophilic pMMApBzMA (*ca.* 78°) and gradually decreases to 74° and 62° for pMMApAAc and pAApAA, respectively. This behaviour is reversed with non-polar CH_2_I_2_ (37° → 45° → 47°, Fig. 7).

### Evaluation of photoinitiator reactivity by photo-DSC

**Fig. 8:**
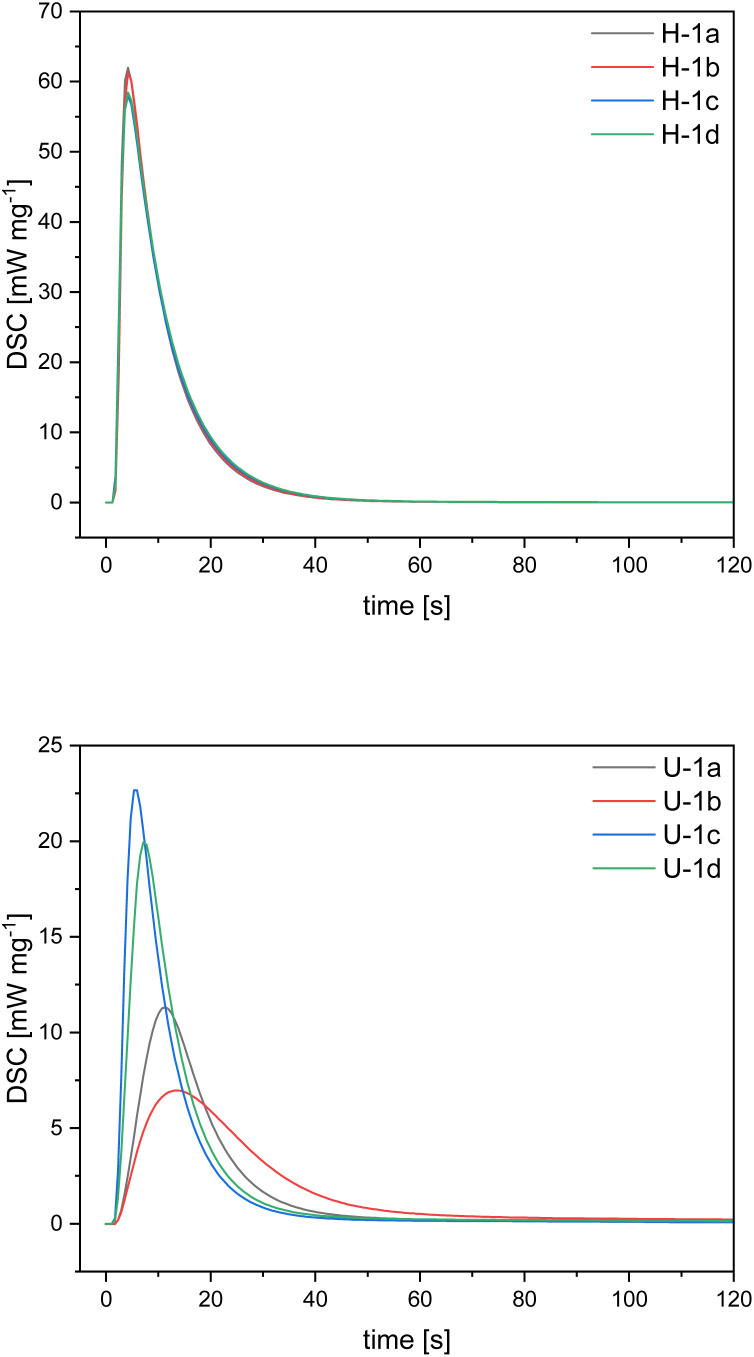
Photo-DSC plot of resin formulations based on HDDA (top) and UDMA/TEGDMA (bottom) containing photoinitiators 1a–d.

Fig. 8 shows that the reaction heats for polymerizations of HDDA (1,6-hexanediol diacrylate) with 1a–d are rather similar with values of 601–613 mJ mg^−1^ (24.5 mW cm^−2^, polychromatic, mercury pressure lamp). The *t*_95%_ values (the time required to reach 95% of the total heat release) are between 26 and 28 s.

In contrast the UDMA/TEGDMA-based resin (urethane dimethacrylate/triethylene glycol dimethacrylate, 70 : 30 wt%), 1a–d exhibit pronounced differences. As illustrated in Fig. 8 and Table 1, initiators 1c (233 mJ mg^−1^; 29.4 s) and 1d (233 mJ mg^−1^; 35.4 s) show significantly higher total reaction enthalpies and demonstrate accelerated polymerization kinetics compared to initiators 1a (184 mJ mg^−1^; 43.2 s) and 1b (182 mJ mg^−1^; 58.2 s). This suggests that the rapid chain growth induced by the HDDA acrylate overrules the reactivity of the photoinitiator. Conversely, the UDMA/TEGDMA system, being methacrylate-based and characterized by slower polymerization kinetics, displays a stronger dependency on the initiator structure and efficiency. The enhanced curing rate and increased exothermicity observed for the longer-chain initiators (1c and 1d) may be attributed to several factors, including improved miscibility with the resin matrix, increased absorption efficiency in the relevant spectral range, or reduced oxygen inhibition effects. The latter may arise from steric hindrance caused by bulkier initiator structures, which could limit the diffusion of atmospheric oxygen into the polymerizing network. Leaching was monitored by determining the total phosphorus content (ICP-MS, inductively coupled plasma mass spectrometry of MeCN extracts of the cured materials (initiator content 2%)). Accordingly, this analysis includes unreacted photoinitiator and phosphorus-containing degradation products. The phosphorus content is highest for 1a, gradually decreasing for 1b and 1c; the latter being basically identical for 1d. This somehow follows the trend that longer-chain molecules exhibit reduced leaching (see SI for details). Additional factors are the higher molar P content in 1b*vs.* the remaining derivatives, the differing bulkiness of 1a–d, and (slightly) different quantum yields.

**Table 1:** Results of photo-DSC measurements for the resins with photoinitiators 1a–d

Bis-BAPO	Δ*H*_polym._/mJ mg^−1^		*t*_95%_/s	
	HDDA	UDMA/TEGDMA	HDDA	UDMA/TEGDMA
1a	603	184	26.4	43.8
1b	601	182	27.0	58.2
1c	602	233	28.2	29.4
1d	613	233	28.2	35.4

## Conclusions

Wavelength-selective activation of simply accessible bis-α,ω-bisacylphosphane oxides provides a straightforward and efficient strategy to access polymers with highly tunable properties. The stepwise and orthogonal photocleavage of the BAPO and MAPO motifs of the newly developed photoinitiators enables precise sequence control using standard monomers, allowing the synthesis of hydrophilic, lipophilic, and amphiphilic polymer architectures in a modular fashion.

However, the photoinitiator itself (depending on the character of the monomers) has a decisive influence on the character of the products although being present at low concentration. This is demonstrated by differences in curing kinetics,^13^ surface properties, and migration behavior. These findings show that photoinitiators with a pre-defined structural motive serve as viable templates for constructing functional polymer networks with tailored performance.

## Ulrich Schubert dedication

This contribution comprises key aspects compatible with Professor Schubert's scientific work and philosophy. His research has consistently combined innovative molecular and inorganic synthesis with thorough materials characterization to establish clear structure–property relationships.

## Author contributions

R. R. and T. W. conceived the idea, designed the overall experiments, and wrote the manuscript (lead). D. G. and M. W. were responsible for experimental investigations (support). R. C. F. collected the X-ray data and solved the crystal structures. J. M. and T. G. measured the photo-DSC. A. K. performed the theoretical calculations. C. W. measured the migration behavior. G. G and M. H. were in charge for methodology and conceptualization, review and editing of the manuscript, project administration and funding acquisition (lead). All authors discussed the results and commented on the manuscript.

## Conflicts of interest

There are no conflicts to declare.

## Supplementary Material

TA-014-D6TA01719C-s001

TA-014-D6TA01719C-s002

## Data Availability

CCDC 2532017 and 2532018 contain the supplementary crystallographic data for this paper.^14*a*,*b*^ All data necessary to support the findings of this study are available in the supplementary information (SI). Supplementary information is available. See DOI: https://doi.org/10.1039/d6ta01719c.
