# Supplementary material for: Bis-α,ω-bisacylphosphane oxides: simple access to crosslinked polymers with tunable properties
Source: J Mater Chem A Mater. 2026 May 26;14(42):28374–9. doi: 10.1039/d6ta01719c (PMC13254757; doi:10.1039/d6ta01719c)
Supplement: TA-014-D6TA01719C-s001 [file TA-014-D6TA01719C-s001.pdf]

Supplementary Information (SI) for Journal of Materials Chemistry A.
This journal is © The Royal Society of Chemistry 2026

Supporting Information

Bis-α,ω-bisacylphosphine Oxides: Simple Access to Crosslinked Polymers with Tunable Properties.

Renata Raptova,[c] Tanja Wiesner,[a] Daniel Griess,[a,b] Julian Maier,[d] Roland C. Fischer,[a] Anne-Marie Kelterer,[c] Mercedes Linares Moreau,[c] Christoph Walkner,[e] Thomas Griesser,[d] Mathias Wiech,[c] Georg Gescheidt*[c] and Michael Haas*[a,b]

a. Institute of Inorganic Chemistry, Graz University of Technology
Stremayrgasse 9/IV, 8010 Graz (Austria)
E-mail: michael.haas@tugraz.at
b. Christian Doppler Laboratory for New Semiconductor Materials based on Functionalized Hydrosilanes
Stremayrgasse 9/IV, 8010 Graz (Austria).
c. Institute of Physical and Theoretical Chemistry, Graz University of Technology
Stremayrgasse 9/II, 8010 Graz (Austria)
d. Chair of Chemistry of Polymeric Materials, Technical University of Leoben, Otto-Glöckel-Strasse 2, 8700 Leoben, Austria
e. Chair of General and Analytical Chemistry, Technical University of Leoben, Franz-Josef-Strasse 18, 8700 Leoben, Austria

Abstract:

We present an efficient one-pot synthesis of bis-α,ω-bisacylphosphine oxides (Bis-BAPOs) obtained by coupling of α,ω-dibromoalkanes with sodium bis(mesitoyl)-phosphide. The resulting tetrafunctional photoinitiators display absorption characteristics similar to parent BAPO yet allow pairwise, wavelength-selective activation. Stepwise irradiation at λ = 450 nm and λ = 385 nm triggers consecutive α-cleavage of the BAPO and the subsequently generated MAPO (monoacylphosphane oxide) moieties, enabling controlled polymer growth and branching. This strategy enables the preparation of hydrophilic, lipophilic, and amphiphilic polymer materials from standard monomers. Atomic force microscopy and contact-angle measurements reveal pronounced differences in surface chemistry despite homogeneous film morphologies, highlighting the impact of initiator architecture on interfacial properties. Migration analyses and photo-DSC experiments further demonstrate that the molecular structure of the photoinitiator strongly governs curing behaviour, conversion kinetics, and extractability, underscoring the potential of bis-BAPOs as tunable components for advanced photopolymer materials

Table of Contents

Synthesis and Characterization 3
  General Considerations 3
  Compound 1a 3
  Compound 1b 4
  Compound 1c 4
  Compound 1d 5
DFT Calculations 7
  Computational Method 7
  Computational Results 7
Photochemical Characterization 11
  Steady-State Photolysis and Determination of Quantum Yields 11
NMR-Spectroscopy 20
EPR-Spectroscopy 24
Synthesis of Polymers 25
Atomic Force Microscopy 29
Contact Angle Measurements 32
NMR-Spectra 33
  Compound 1a 33
  Compound 1b 35
  Compound 1c 37
  Compound 1d 39
X-Ray Crystallography 41
  General Considerations 41
  Crystallographic Data 41
Leaching Experiments 42
References 43

Synthesis and Characterization

General Considerations

All air- and/or moisture-sensitive reactions were performed under inert conditions using either standard Schlenk techniques or a nitrogen-filled glove box. Solvents were dried by means of a column solvent purification system.[1] All chemicals from commercial sources were used as purchased from chemical suppliers without any further purification. For the measurement of air-sensitive samples, benzene-d_6 and THF-d_8 were dried above a sodium/potassium alloy for 12-hour reflux. In addition, $CDCl_3$ was dried above Na_2SO_4 for 12-hour reflux.
^{1}H-, ^{13}C- and ^{31}P-NMR spectra were recorded either on a Varian INOVA 300, a 200 MHz Bruker AVANCE DPX, a 300 MHz Bruker Avance or a 400 MHz Jeol JNM-ECZL spectrometer with Royal HFX-Probes with auto sampler in C_6D_6, $CDCl_3$ or THF-d_8 solutions and referenced versus TMS using the internal ^{2}H-lock signal of the solvent. Infrared spectra were obtained on a Bruker Alpha-P Diamond ATR spectrometer from the solid sample. Melting points were determined using Stuart SMP50 apparatus and are uncorrected. Elemental analyses were carried out on a Hanau Vario Elementar EL apparatus. UV/Vis absorption spectra were recorded with an Agilent Cary 60 UV/VIS spectrometer.

Compound 1a

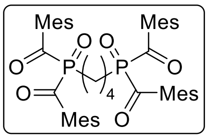

2.16 g of freshly cut sodium (93.95 mmol, 3.00 eq.), 0.40 g of naphthalene (3.13 mmol, 0.10 eq.) and 50 mL THF were placed in a flask and stirred until the color turned deep green. 1.00 g of red phosphorus (31.32 mmol, 1.00 eq., 97% pure) was added and the suspension was stirred overnight to result in a black precipitate (Na_3P). 2.94 mL of ^{t}BuOH (31.32 mmol, 1.00 eq.) were slowly added at 0 °C and stirred for 1h at room temperature. The suspension was again cooled to 0 °C and 10.45 mL of 2,4,6-trimethylbenzoyl chloride (100.22 mmol, 2.00 eq.) were slowly added, whereby the color turned yellowish green. After stirring for 1h at room temperature, the reaction mixture was filtered over celite and the filter cake was washed with more THF. The resulting clear reaction solution was cooled to 0 °C and 1.68 mL of 1,4-dibromobutane (14.09 mmol, 0.45 eq.) was added. After refluxing the reaction solution over night, the solvent was replaced by toluene to remove the resulting salts via filtration. Oxidation was achieved through the addition of 3.20 mL of hydrogen peroxide (31.00 mmol, 2.20 eq.) at 0 °C and stirring for about 2h at room temperature. Aqueous work-up was carried out with a saturated NH_4Cl-solution, separating the organic layer and extracting the aqueous layer three times with diethyl ether. The combined organic phases were dried over Na_2SO_4, filtered and the volatiles removed under vacuum. The crude product was purified by column chromatography with *n*-heptane/ethyl acetate 20/1, yielding 5.89 g (56.58% yield) as a yellow powder.

The analytical data for **1a** are as follows. **mp**: 179-182 °C. **^{1}H NMR** (300 MHz, $CDCl_3$) δ 6.85 (s, 8H; Ar-*H*), 2.28 (s, 12H, Ar-C*H*$_3$), 2.24 (s, 24H, Ar-C*H*$_3$), 2.16 (q, *J* = 4.8 Hz, 4H, C*H*$_2$), 1.69 (t, *J* = 2.8 Hz, 4H, C*H*$_2$). **^{13}C NMR** (75 MHz, $CDCl_3$) δ 216.38 (d, $^1J_{PC}$ = 53.2 Hz, *C*=O), 141.49 (Ar-*C*), 136.24 (Ar-*C*), 135.70 (Ar-*C*), 129.38 (Ar-*C*), 26.21 (d, J_{PC} = Hz, *C*H$_2$), 25.50 (d, J_{PC} = 53.3 Hz, *C*H$_2$), 22.88 (dd, J_{PC} = 15.0, 4.0 Hz, *C*H$_2$), 21.33 (Ar-*C*H$_3$), 19.87 (Ar-*C*H$_3$). **^{31}P NMR** (121 MHz, $CDCl_3$) δ 26.29 (quint).**^{31}P{^{1}H} NMR** (121 MHz, $CDCl_3$) δ 26.30 (s). **IR** (powder): ν(C=O) 1663, 1636, 1603 cm^{-1}. **UV/Vis**: λ [nm], ε[L•mol^{-1}•cm^{-1}] (toluene) 371, 1200; 403, 968. **Elem. Anal.** Calcd for $C_{44}H_{52}O_6P_2$: C, 71.53%; H, 7.09%. Found: C, 71.19%; H, 7.26%.

Compound 1b

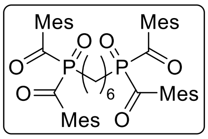

2.16 g of freshly cut sodium (93.95 mmol, 3.00 eq.), 0.40 g of naphthalene (3.13 mmol, 0.10 eq.) and 50 mL THF were placed in a flask and stirred until the color turned deep green. 1.00 g of red phosphorus (31.32 mmol, 1.00 eq., 97% pure) was added and the suspension was stirred overnight to result in a black precipitate (Na_3P). 2.94 mL of ^tBuOH (31.32 mmol, 1.00 eq.) were slowly added at 0 °C and stirred for 1h at room temperature. The suspension was again cooled to 0 °C and 10.45 mL of 2,4,6-trimethylbenzoyl chloride (100.22 mmol, 2.00 eq.) were slowly added, whereby the color turned yellowish green. After stirring for 1h at room temperature, the reaction mixture was filtered over celite and the filter cake was washed with more THF. The resulting clear reaction solution was cooled to 0 °C and 2.18 mL of 1,6-dibromohexane (14.09 mmol, 0.45 eq.) was added. After refluxing the reaction solution over night, the solvent was replaced by toluene to remove the resulting salts via filtration. Oxidation was achieved through the addition of 3.20 mL of hydrogen peroxide (31.00 mmol, 2.20 eq.) at 0 °C and stirring for about 2h at room temperature. Aqueous work-up was carried out with a saturated NH_4Cl-solution, separating the organic layer and extracting the aqueous layer three times with diethyl ether. The combined organic phases were dried over Na_2SO_4, filtered and the volatiles removed under vacuum. The crude product was purified by column chromatography with *n*-heptane/ethyl acetate 20/1, yielding 6.18 g (57.19% yield) as a yellow powder.

The analytical data for **6b** are as follows. **mp**: 160-162 °C. **^{1}H NMR** (300 MHz, $CDCl_3$) δ 6.84 (s, 8H, Ar-*H*), 2.27 (s, 12H, Ar-CH_3), 2.24 (s, 24H, Ar-CH_3), 2.18 (q, J = 5.9 Hz, 4H, CH_2), 1.58 (s, 4H, CH_2), 1.34 (s, 4H, CH_2). **^{13}C NMR** (75 MHz, $CDCl_3$) δ 216.68 (d, $^1J_{PC}$ = 52.9 Hz, *C*=O), 141.35 (Ar-*C*), 136.33 (d, J_{PC} = 40.2 Hz, Ar-*C*), 135.69 (Ar-*C*), 129.34 (Ar-*C*), 30.74 (d, J_{PC} = 13.6 Hz, *C*H_2), 26.64 (d, J_{PC} = 53.5 Hz, *C*H_2), 21.33 (Ar-*C*H_3), 21.24 (d, J_{PC} = 4.5 Hz, *C*H_2), 19.87 (Ar-*C*H_3). **^{31}P NMR** (121 MHz, $CDCl_3$) δ 27.61 (quint).**^{31}P{^{1}H} NMR** (121 MHz, $CDCl_3$) δ 27.32 (s). **IR** (powder): ν(C=O) 1669, 1650, 1605 cm^{-1}. **UV/Vis:** λ [nm], ε[L•mol^{-1}•cm^{-1}] (toluene) 371, 1080; 403, 894. **Elem. Anal.** Calcd for $C_{46}H_{56}O_6P_2$: C, 72.04%; H, 7.36%. Found: C, 71.67%; H, 7.34%.

Compound 1c

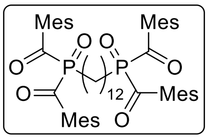

2.16 g of freshly cut sodium (93.95 mmol, 3.00 eq.), 0.40 g of naphthalene (3.13 mmol, 0.10 eq.) and 50 mL THF were placed in a flask and stirred until the color turned deep green. 1.00 g of red phosphorus (31.32 mmol, 1.00 eq., 97% pure) was added and the suspension was stirred overnight to result in a black precipitate (Na_3P). 2.94 mL of ^tBuOH (31.32 mmol, 1.00 eq.) were slowly added at 0 °C and stirred for 1h at room temperature. The suspension was again cooled to 0 °C and 10.45 mL of 2,4,6-trimethylbenzoyl chloride (100.22 mmol, 2.00 eq.) were slowly added, whereby the color turned yellowish green. After stirring for 1h at room temperature, the reaction mixture was filtered over celite and the filter cake was washed with more THF. The resulting clear reaction solution was cooled to 0 °C and 4.82 g of 1,12-dibromododecane (14.09 mmol, 0.45 eq.) was added. After refluxing the reaction solution over night, the solvent was replaced by toluene to remove the resulting salts via filtration. Oxidation was achieved through the addition of 3.20 mL of hydrogen peroxide (31.00 mmol, 2.20 eq.) at 0 °C and stirring for about 2h at room temperature. Aqueous work-up was carried out with a saturated NH_4Cl-solution, separating the organic layer and extracting the aqueous layer three times with diethyl ether. The combined organic phases were dried over Na_2SO_4, filtered and the volatiles removed under vacuum. The crude product was purified by column chromatography with *n*-heptane/ethyl acetate 20/1, yielding 7.58 g (62.19% yield) as a yellow oil.

The analytical data for **6c** are as follows. **^{1}H NMR** (300 MHz, C_6D_6) δ 6.56 (s, 8H, Ar-*H*), 2.38 (s, 24H, Ar-C*H*$_3$), 2.26 (m, 4H, C*H*$_2$), 1.97 (s, 12H, Ar-C*H*$_3$), 1.71 (m, 4H, C*H*$_2$), 1.13 (s, 16H, C*H*$_2$). **^{13}C NMR** (75 MHz, C_6D_6) δ 217.52 (d, $^1J_{PC}$ = 53.3 Hz, *C*=O), 141.03 (Ar-*C*), 137.36 (d, J_{PC} = 40.3 Hz, Ar-*C*), 136.11 (Ar-*C*), 129.52 (Ar-*C*), 31.41 (d, J_{PC} = 12.9 Hz, *C*H$_2$), 29.89 (d, J_{PC} = 14.2 Hz, *C*H$_2$), 29.42 (*C*H$_2$), 27.17 (*C*H$_2$), 26.46 (*C*H$_2$), 21.81 (d, J_{PC} = 4.5 Hz, *C*H$_2$), 21.10 (Ar-*C*H$_3$), 20.09 (Ar-*C*H$_3$). **^{31}P NMR** (121 MHz, C_6D_6) δ 28.03 (quint).**^{31}P{^{1}H} NMR** (121 MHz, C_6D_6) δ 28.03 (s). **IR** (oil): ν(C=O) 1672, 1654, 1607 cm^{-1}. **UV/Vis:** λ [nm], ε[L•mol^{-1}•cm^{-1}] (toluene) 372, 1240; 404, 1040. **Elem. Anal.** Calcd for $C_{52}H_{68}O_6P_2$: C, 73.39%; H, 8.05%. Found: C, 73.04%; H, 7.89%.

Compound 1d

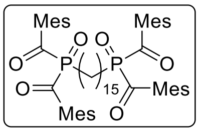

Method A: 0.63 g of freshly cut sodium (27.69 mmol, 3.00 eq.), 0.12 g of naphthalene (0.92 mmol, 0.10 eq.) and 15 mL THF were placed in a flask and stirred until the color turned deep green. 0.28 g of red phosphorus (9.23 mmol, 1.00 eq., 97% pure) was added and the suspension was stirred overnight to result in a black precipitate (Na_3P). 0.87 mL of ^{t}BuOH (9.23 mmol, 1.00 eq.) were slowly added at 0 °C and stirred for 1 h at room temperature. The suspension was again cooled to 0 °C and 3.09 mL of 2,4,6-trimethylbenzoyl chloride (18.46 mmol, 2.00 eq.) were slowly added, whereby the color turned yellowish green. After stirring for 1h at room temperature, the reaction mixture was filtered over celite and the filter cake was washed with more THF. The resulting clear reaction solution was cooled to 0 °C and 1.54 g of 1,15-dibromopentadecane (4.15 mmol, 0.45 eq.) was added. After refluxing the reaction solution over night, the solvent was replaced by toluene to remove the resulting salts via filtration. Oxidation was achieved through the addition of 2.09 mL of hydrogen peroxide (30 wt% solution, 20.31 mmol, 2.20 eq.) at 0 °C and stirring for about 2 h at room temperature. Aqueous work-up was carried out with a saturated NH_4Cl-solution, separating the organic layer and extracting the aqueous layer three times with diethyl ether. The combined organic phases were dried over Na_2SO_4, filtered and the volatiles removed under vacuum. The crude product was purified by column chromatography with *n*-heptane/ethyl acetate 20/1, yielding 1.2 g (32.2% yield) as a yellow oil.

Mathod B: 0.50 g of isolated Mes-BAP-Na (1.43 mmol, 1 eq) were weighed into a flask and dissolved in THF. The yellow solution was cooled to 0° C and 0.24 g 1,15-dibromopentadecane (0.64 mmol, 0.45 eq) was dissolved in THF and added to the reaction solution. After refluxing the reaction solution over night, the solvent was replaced by toluene to remove the resulting salts via filtration. Oxidation was achieved through the addition of 0.32 mL of hydrogen peroxide (30% solution, 3.16 mmol, 2.20 eq.) at 0 °C and the reaction was stirred for 3h at room temperature. Aqueous work-up was carried out with a saturated NH_4Cl-solution, separating the organic layer and extracting the aqueous layer three times with diethyl ether. The combined organic phases were dried over Na_2SO_4, filtered and the volatiles removed under vacuum. The crude product was purified by column chromatography with *n*-heptane/ethyl acetate (20/1 to 4/1), yielding 246.50 mg (42% yield) as a yellow solid.

The analytical data for **1d** are as follows. **mp**: 100-102 °C.**^{1}H NMR** (400 MHz, C_6D_6) δ 6.56 (s, 8H, Ar-*H*), 2.37 (s, 24H, Ar-C*H*$_3$), 2.28 – 2.20 (m, 4H, C*H*$_2$), 1.98 (s, 12H, Ar-C*H*$_3$), 1.76 – 1.65 (m, 4H, C*H*$_2$), 1.28 – 1.14 (m, 22H, C*H*$_2$).**^{13}C NMR** (101 MHz, $CDCl_3$) δ 216.51 (d, $^1J_{PC}$ = 53.3 Hz, *C*=O), 141.13 (Ar-*C*), 136.21(d, J_{PC} = 40.0 Hz, Ar-*C*), 135.54 (Ar-*C*), 129.19 (Ar-*C*), 31.05 (d, J_{PC} = 13.6 Hz, *C*H$_2$), 30.83 (*C*H$_2$), 29.58 (*C*H$_2$), 29.51 (*C*H$_2$), 29.24 (d, J_{PC} = 30.3 Hz, *C*H$_2$), 26.55 (d, J_{PC} = 53.0 Hz, *C*H$_2$), 21.18 (Ar-*C*H$_3$), 21.13 (*C*H$_2$), 21.09 (*C*H$_2$), 19.74 (Ar-*C*H$_3$).**^{31}P{^{1}H}** NMR (162 MHz, C_6D_6) δ 28.57 (s).**^{31}P** NMR (162 MHz, C_6D_6) δ 28.57 (quint) **IR** (oil): ν(C=O) 1668,

1641, 1603 cm^{-1}. **UV/Vis:** λ [nm], ε[L•mol^{-1}•cm^{-1}] (toluene) 372, 1180; 404, 970. **Elem. Anal.** Calcd for $C_{55}H_{74}O_6P_2$: C, 73.96%; H, 8.35%. Found: C, 73.70%; H, 8.55%.

DFT Calculations

Computational Method

In this work, the composite PBEh-3c functional[2] was used that combines the hybrid DFT functional PBE0 with Grimme's D3BJ dispersion correction using Becke-Johnson damping and the geometrical counterpoise method applying the m-def2-SVP basis set for all atoms. To confirm the minimum quality of the geometries, harmonic frequencies were computed. All calculations were performed with the program ORCA 6.0.1.[3] The conductor-like polarizable continuum (CPCM) model[4] was applied throughout this study for toluene. For the optimized geometries, 20 vertical excitations were then computed with the same computational method in toluene. For the simulation of the UV/Vis spectra, a Gaussian broadening (σ = 1500 cm^{-1}) was applied using the program orca_asa.[5] To correct for the systematic shift of the computed PBEh-3c bands, an offset of 30 nm was added in the simulated spectra in the pictorial comparison of the experimental and theoretical spectrum. The respective orbitals were drawn with contour values of 0.04 a.u. using the program Avogadro.[6]

Computational Results

Geometries. In agreement with the crystal structures, compound **1a** remains in its all-trans conformation of the chain connecting the two photoinitiator units, which leads to a trans orientation of both P=O groups (O=P-P=O 173.5°) with the P-P distance of 6.91 Å and the mesitoyl moieties in a T-like orientation with a dihedral angle of 44° to each other at both sides. In contrast, the optimized crystal structure of **1b** has a tgtg't conformation, also with trans orientation of the P=O groups (O=P-P=O 179.5°) separating the photoinitiator moieties by a P-P distance of 8.91 Å. Again, the mesitoyl moieties are oriented in a T-like position with ca. 33° dihedral angle at both sides of the chain. To check if the tgtg't conformation of **1b** is the lowest energy conformation in solution, we optimized the all-trans conformer of **1b** in the solvent toluene, and it was found to be 21.35 kJ/mol more stable than the optimized tgtg't crystal structure. Thus, the tgtg't conformation is not the global minimum, which might be a consequence of the stacking in the crystal structure. In the lower energy all-trans conformation, the P-P distance is somewhat larger (9.42 Å) with well-separated photoinitiator moieties. Similarly, the all-trans conformation is more stable for **1a**, but only by 1.07 kJ/mol compared to the tgt conformation which would result in a spectrum that combines both conformations. Since the C-C bonds are freely rotable in solution, we expect the all-trans conformations of **1b** to be the dominant conformations in the solvent conforming with the experimental the UV spectra.

Calculated UV/Vis Data. Compound **1a** shows one long-wavelength band with low intensity around 350 nm and a high-intensity band at around 270 nm. The first band is formed by four n-π* vertical excitations, each of them consisting of linear combinations of transitions at the C=O and P=O groups, but also including excitations at the mesitoyl moieties. The S1 band includes the HOMO-LUMO transition. Details are given in Table S1, and the relevant orbitals are depicted in Table S2. The second band of **1a** consists of π-π* transitions including various contributions from the C=O and mesitoyl groups.

Table S1. The first seven computed vertical excitations of **1a** in all-trans conformation computed with the PBEh-3c method in toluene. Linear combinations of the molecular orbitals are given for c^2>0.1. The HOMO is orbital number 196, the LUMO is orbitals number 197.

1a	eV	nm	f	MO1	c^2	MO2	c^2	MO3	c^2
S_1	3.493	354.9	0.0095	196a$\rightarrow$197a	0.17	187a$\rightarrow$197a	0.14	186a$\rightarrow$197a	0.11
S_2	3.499	354.3	0.0032	188a$\rightarrow$198a	0.20	195a$\rightarrow$198a	0.16	186a$\rightarrow$198a	0.10
S_3	3.558	348.4	0.0013	196a$\rightarrow$200a	0.21	196a$\rightarrow$199a	0.18		
S_4	3.559	348.3	0.0020	195a$\rightarrow$200a	0.20	195a$\rightarrow$199a	0.20		
S_5	4.588	270.2	0.0391	191a$\rightarrow$197a	0.31	190a$\rightarrow$197a	0.18	191a$\rightarrow$198a	0.11
S_6	4.608	269.1	0.0500	189a$\rightarrow$198a	0.28	194a$\rightarrow$198a	0.23	189a$\rightarrow$197a	0.11
S_7	4.652	266.5	0.4113	195a$\rightarrow$198a	0.17	188a$\rightarrow$198a	0.13	193a$\rightarrow$197a	0.10

Table S2. Relevant orbitals of **1a** in all-trans conformation with contour values of 0.04 a.u, computed with the PBEh-3c method in toluene. Orbital numbers are given additionally for readers convenience.

	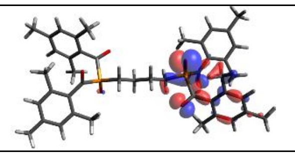	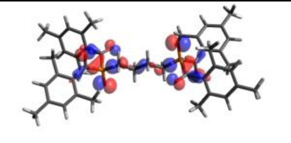	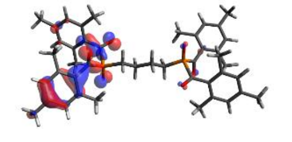
	HOMO-12=184	HOMO-10=186	HOMO-9=187
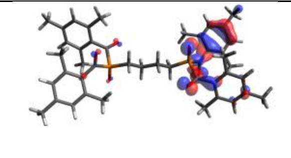	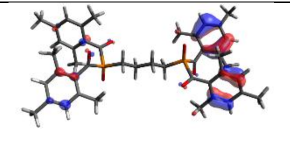	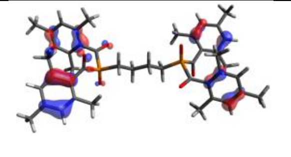	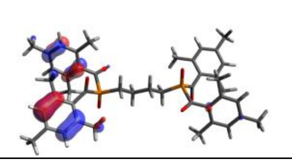
HOMO-8=188	HOMO-7=189	HOMO-6=190	HOMO-5=191
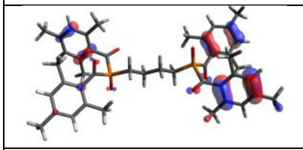	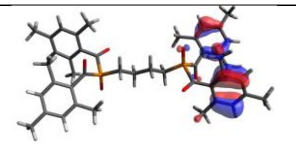	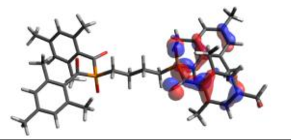	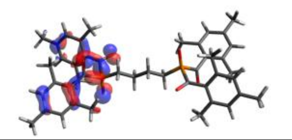
HOMO-3=193	HOMO-2=194	HOMO-1=195	HOMO=196
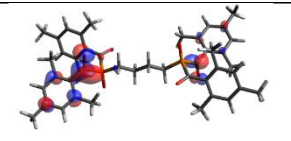	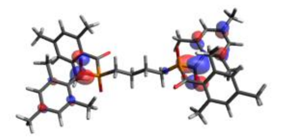	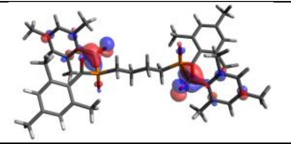	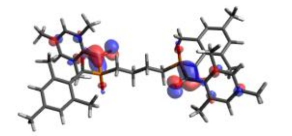
LUMO=197	LUMO+1=198	LUMO+2=199	LUMO+3=200

The calculated UV/Vis spectrum of the lower-energy *all-trans* conformer of **1b** also agrees well with the experimental spectrum showing two bands in the high-wavelength region (see Figure S1) which are built from the first four transitions (excitation data in Table S3). For a better understanding, we show the negative depleting and positive forming transition densities upon excitation, because the individual transitions are linear combinations of many orbitals (Table S4). The two lowest n–π* excitations (S_1 and S_2) are local excitations at one side of the chains forming the band at 377 nm (calculated value). The orbitals are well separated through the longer chains of **1b**. The next two n–π* excitations (S_3 and S_4) form a slightly blue-shifted band at 340 nm stemming from excitations at both sides of the chain. Excitations S_5-S_8 are the main contributions for the intense, short-wavelength band at ca. 300 nm and consist of π–π* transitions. To conclude, the chain can well separate the two PIs and such produce low-energy locally excited bands.

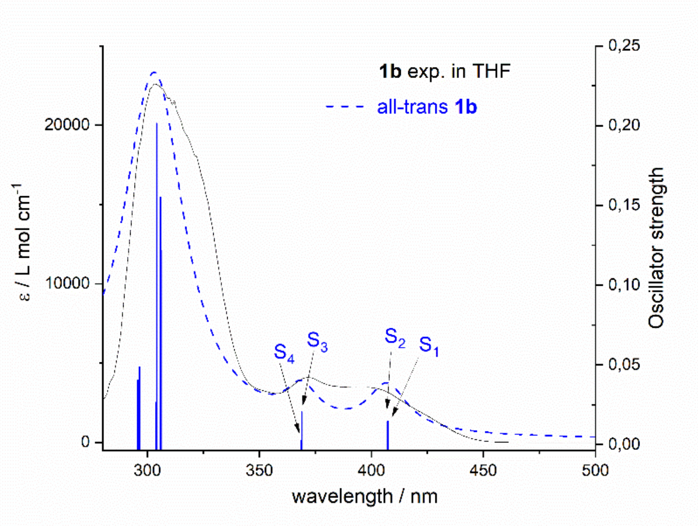

Figure S1. Simulated UV/Vis spectrum of the all-trans conformer of 1b (blue) compared to the experimental spectrum in toluene.

Table S3. The first seven computed vertical excitations of **1b** in all-trans conformation, computed with the PBEh-3c method in toluene. Linear combinations of the molecular orbitals are given for c^2>0.1. Orbital numbers are given additionally for readers convenience.

1b	eV	nm	f	MO1	c^2	MO2	c^2	MO3	c^2
S_1	3.286	377.3	0.0147	203a→205a	0.17	196a→205a	0.13	203a→206a	0.11
S_2	3.288	377.1	0.0146	204a→206a	0.17	195a→206a	0.13	204a→205a	0.11
S_3	3.658	338.9	0.0205	194a→205a	0.17	193a→206a	0.14	196a→207a	0.11
S_4	3.662	338.6	0.0025	194a→206a	0.17	193a→205a	0.15	204a→208a	0.12
S_5	4.491	276.0	0.1218	202a→205a	0.26	197a→205a	0.18	202a→206a	0.17
S_6	4.496	275.8	0.1552	201a→206a	0.21	198a→206a	0.19	201a→205a	0.14
S_7	4.524	274.1	0.2014	195a→206a	0.19	204a→205a	0.16	203a→206a	0.15

Table S4. Relevant first six transition densities of **1b** in *all-trans* conformation with contour values of 0.001 a.u, computed with the PBEh-3c method in toluene. Depleting, negative transition densities are shown in red, positive transition densities are shown in blue.

	Excitation from	Excitation to	Character
S1	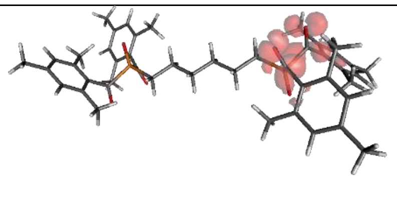	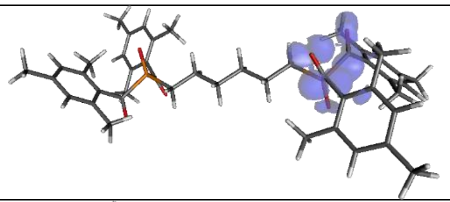	Local n–π* at C=O groups at one side of the chain
S2	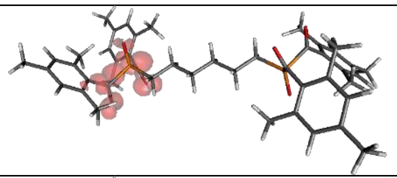	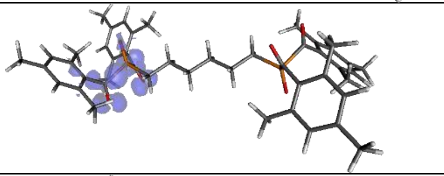	Local n–π* at C=O groups at other side of the chain
S3	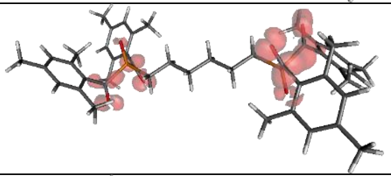	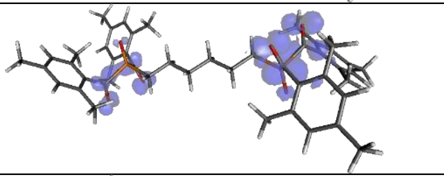	n–π* at C=O on both sides of the chain
S4	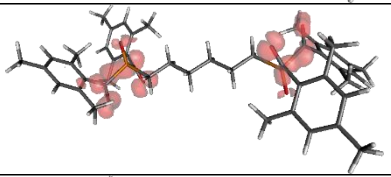	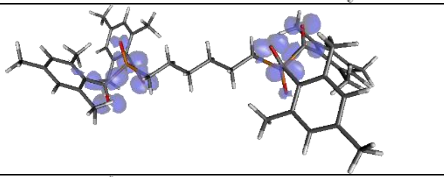	n–π* at C=O on both sides of the chain
S5	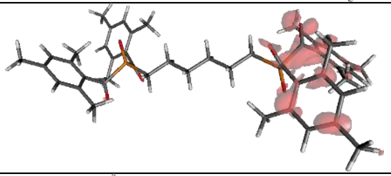	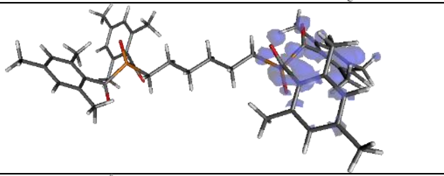	Local π–π* at one side of the chain
S6	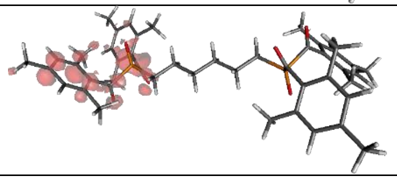	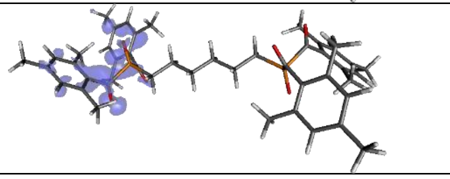	Local π–π* at the other side of the chain

Photochemical Characterization

Steady-State Photolysis and Determination of Quantum Yields

UV/Vis spectra were acquired on a fiber-coupled TIDAS S500 UV-Vis spectrometer equipped with a 1024-pixel diode-array detector (J&M Analytik AG, Essingen, Germany). Photobleaching experiments were performed with 385 nm and 450 nm LEDs (Roithner Lasertechnik GmbH, Vienna, Austria). All LEDs were operated using a Keithley 224 programmable constant current source. The optical output power of the LEDs was determined using a spectrophotometer (GLSpectis, GL Optics, Germany) equipped with an integrating sphere (Ulbricht sphere). Samples were LED-irradiated perpendicular to the optical path of the spectrometer. The setup and method are described in more detail in reference [7].

The determination of the photobleaching quantum yields follows the steps given below:

1. The photon flux emitted at 385 nm (11.3 mA LED current) was determined using ferrioxalate actinometry. 3 mL of a 11.2 mM ferrioxalate solution in 0.05 M H_2SO_4 was light-irradiated, while recording UV/Vis spectra every 2 seconds. The absorbance at 410 nm was plotted against time and fitted linearly. Following the procedure by Lehóczki *et al.*[8], the slope of the fitted line yields a photon flux of 5.04351 × 10^{-6} mol·s^{-1} assuming a quantum yield of ϕ = 1.2 and an extinction coefficient at the observation wavelength of $\varepsilon_{obs.}$ = 146 M^{-1} cm^{-1} according to Equation 1:

$$I_0 = \frac{slope}{\varepsilon_{obs}\,\phi} \qquad (1)$$

2. The molar extinction coefficients of **1a-b** at 385 nm and 371 nm were measured in tetrahydrofuran (THF) and in a THF/methyl methacrylate (MMA) mixture (1:1 by volume). Due to a slight solvatochromic shift, only the extinction coefficients in the THF/MMA mixture were employed for the subsequent calculations of the initiator concentrations.

3. Samples with a concentration of approximately 0.144 mM (corresponding to an initial absorbance of ~0.1) in 3 mL of the THF/MMA mixture. The solutions were transferred to standard fluorescence quartz cuvettes with a 1 cm optical pathlength, and a magnetic stir bar was added. Oxygen was removed by bubbling the solutions with argon for 5 minutes. The samples were then irradiated in a single step under continuous stirring for at least 15 minutes using the 385 nm LED (11.3 mA LED current). Absorption spectra were recorded at 2-second intervals.

4. The initial sample concentration was calculated using the absorbance at 385 nm expected from the previously determined molar extinction coefficients. The decay of the absorbance at the 371 nm maximum was plotted as a function of time and fitted using the exponential function $y=y_0+A\cdot\exp(R_0\cdot x)$, where R_0 corresponds to the observed rate constant k_{fit}. Using the initial absorbance A_0 at 385 nm and initial concentration c_0, the quantum yield (Φ) is calculated by:

$$\Phi = \frac{k_{fit}\,c_0}{I_0(1-10^{-A'_i})} \qquad (2)$$

The corresponding UV/Vis spectra are shown below.

Ferrioxalate actinometry

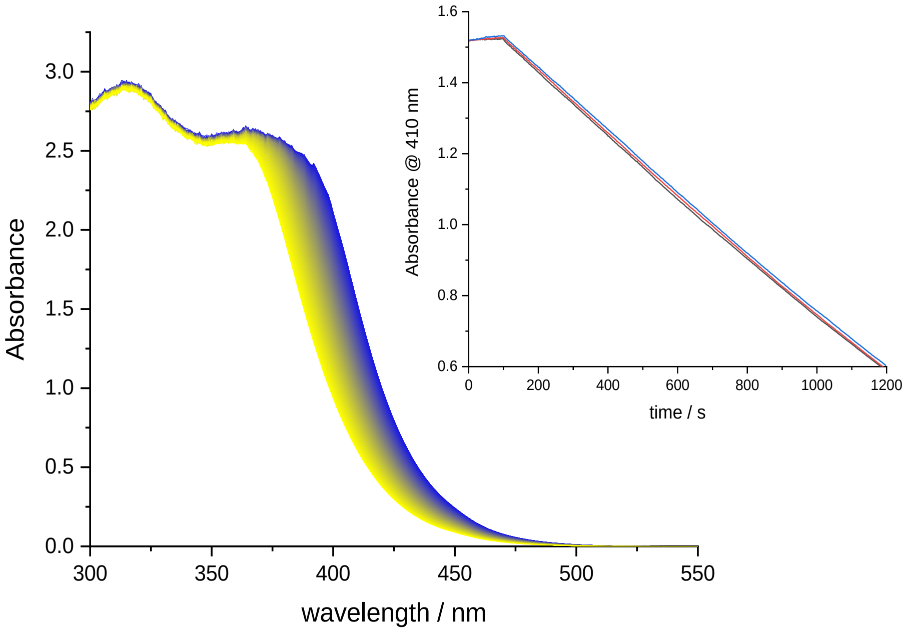

Figure S2. UV/Vis spectra of a 11.2 mM ferrioxalate solution in 50 mM H_2SO_4 upon irradiation at 385 nm (11.3 mA LED current). The inset shows the decreasing absorbance at 410 nm as a function of irradiation time for three replicate measurements. The region over which a linear fit of the absorbance is performed corresponds to the irradiation time in the consecutive photobleaching experiments.

Compound 1a

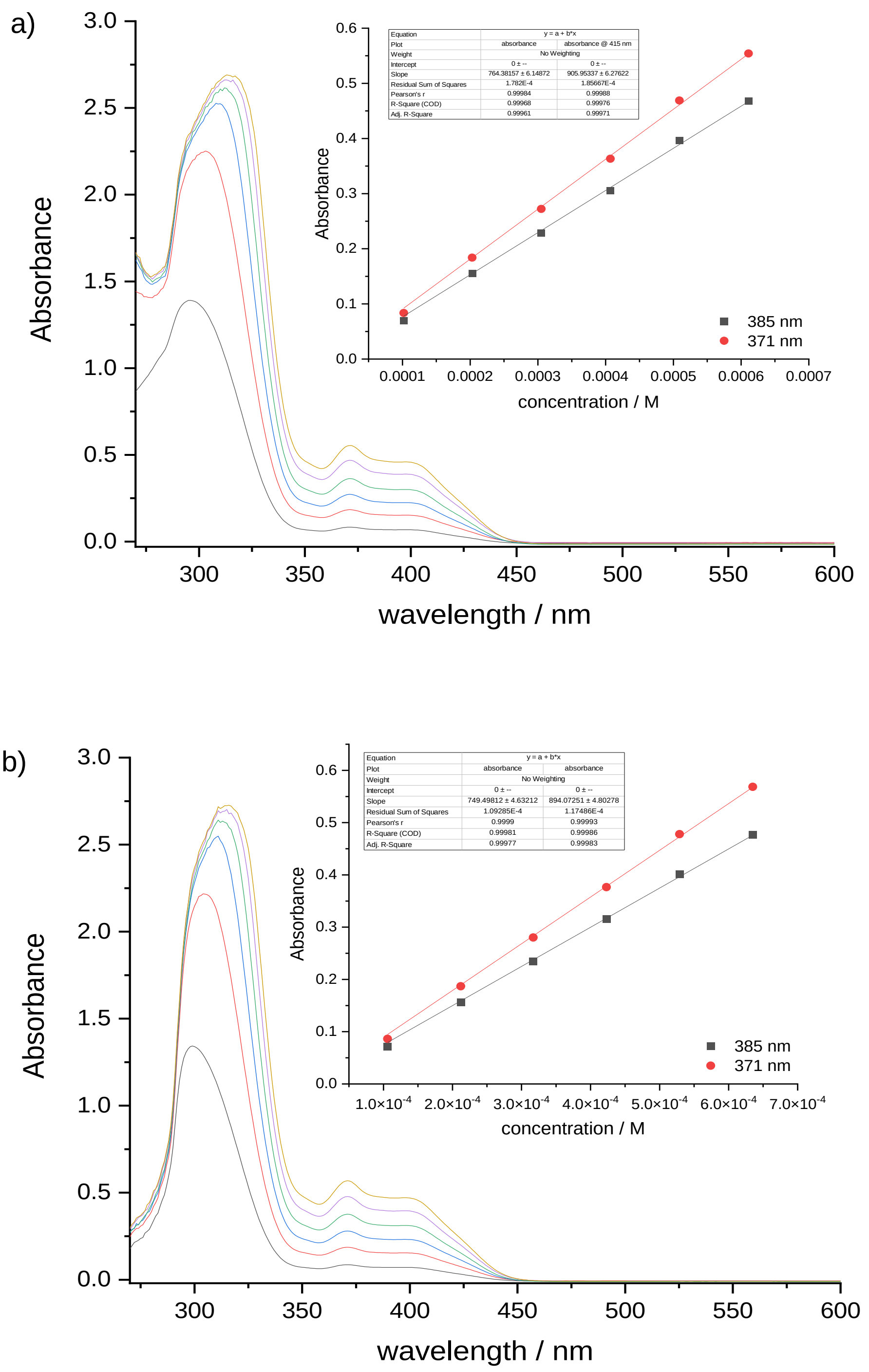

Figure S3. UV–vis spectra of 0.10 – 0.60 mM **1a** and fitted 385 nm and 371 nm absorbances for the determination of extinction coefficients (insert). a) in THF; b) in THF/MMA (1:1, v/v).

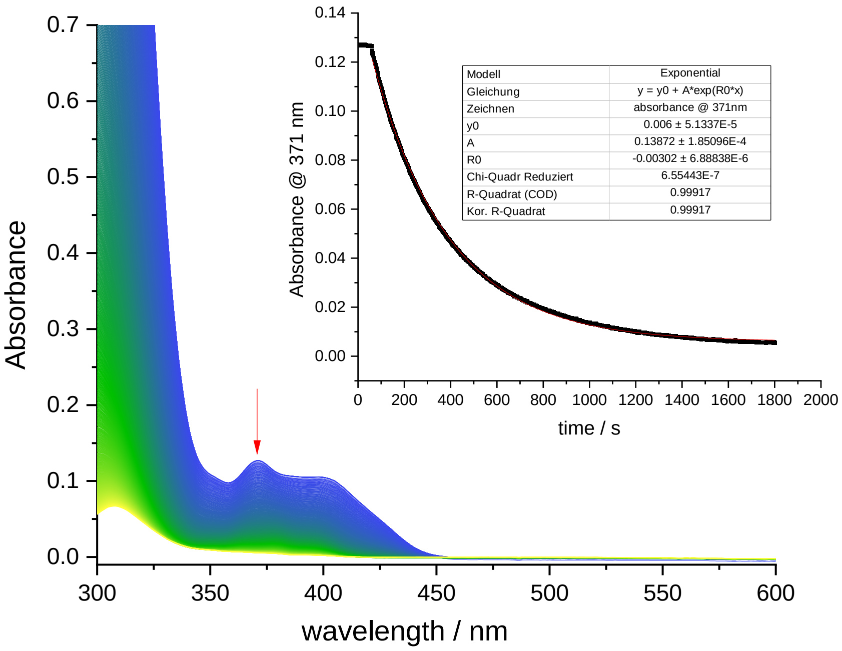

Figure S4. UV/Vis spectra of **1a** upon 385 nm LED irradiation, collected at 2-second intervals. The inset shows the decaying 371 nm absorbance maximum (red arrow) as a function of irradiation time. Conditions: 3 mL of 0.14 mM **1a** in THF/MMA (1:1, v/v), oxygen removed by bubbling with argon for 5 min.

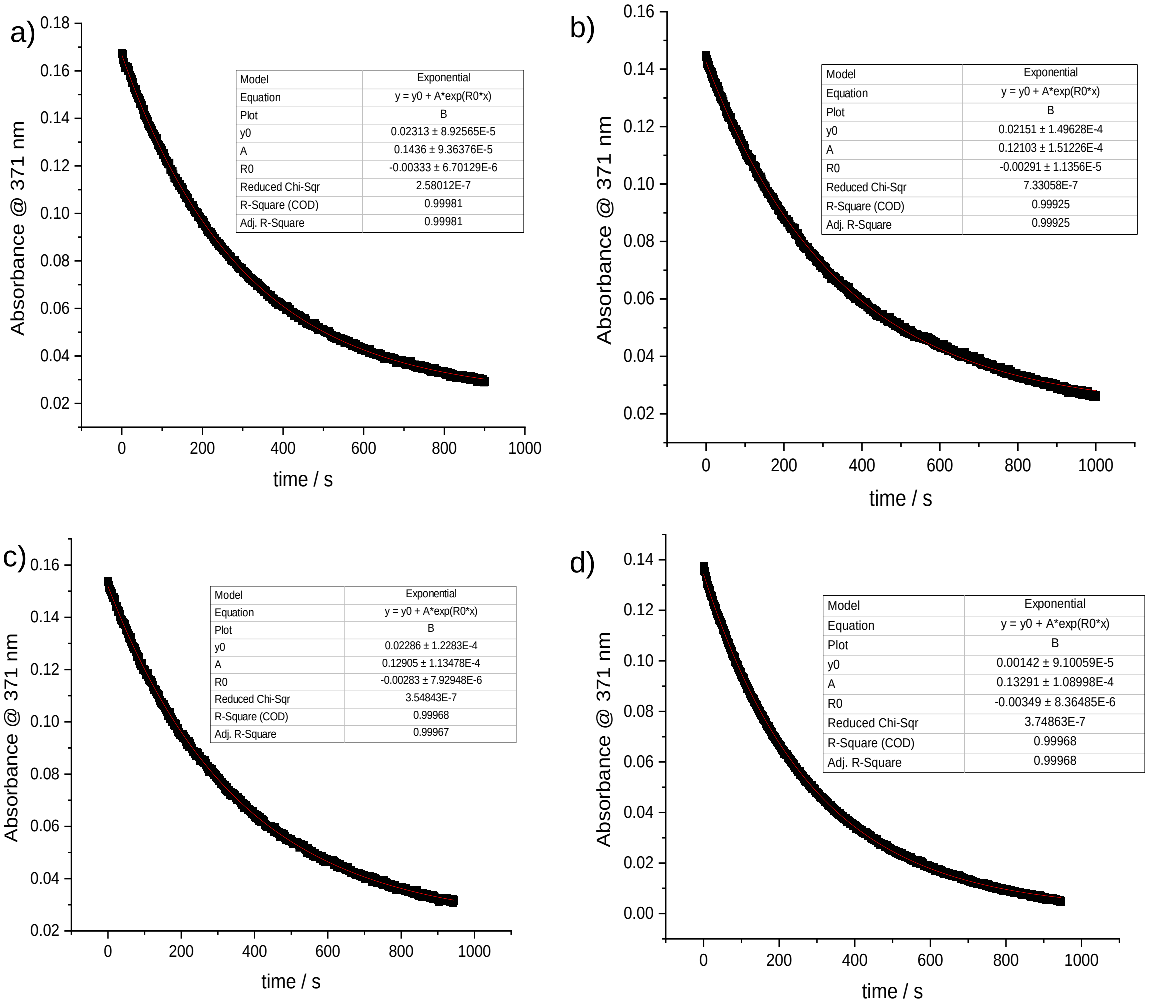

Figure S5. Decays of the 371 nm absorbance maximum of **1a** upon LED-irradiation at 385 nm. Panels a)–d) show four independent repetitions of the experiment.

Table S5. Summary of parameters used for the quantum yield calculation of **1a**.

	A_0 (385 nm)	A_0 (371 nm)	C_0 / M x 10^{-4}	k_{fit}	Φ
1	0.107	0.128	1.44	-0.00302	0.40
2	0.144	0.167	1.92	-0.00333	0.46
3	0.124	0.145	1.66	-0.00291	0.39
4	0.131	0.154	1.75	-0.00283	0.38
5	0.115	0.137	1.53	-0.00349	0.46

Compound 1b

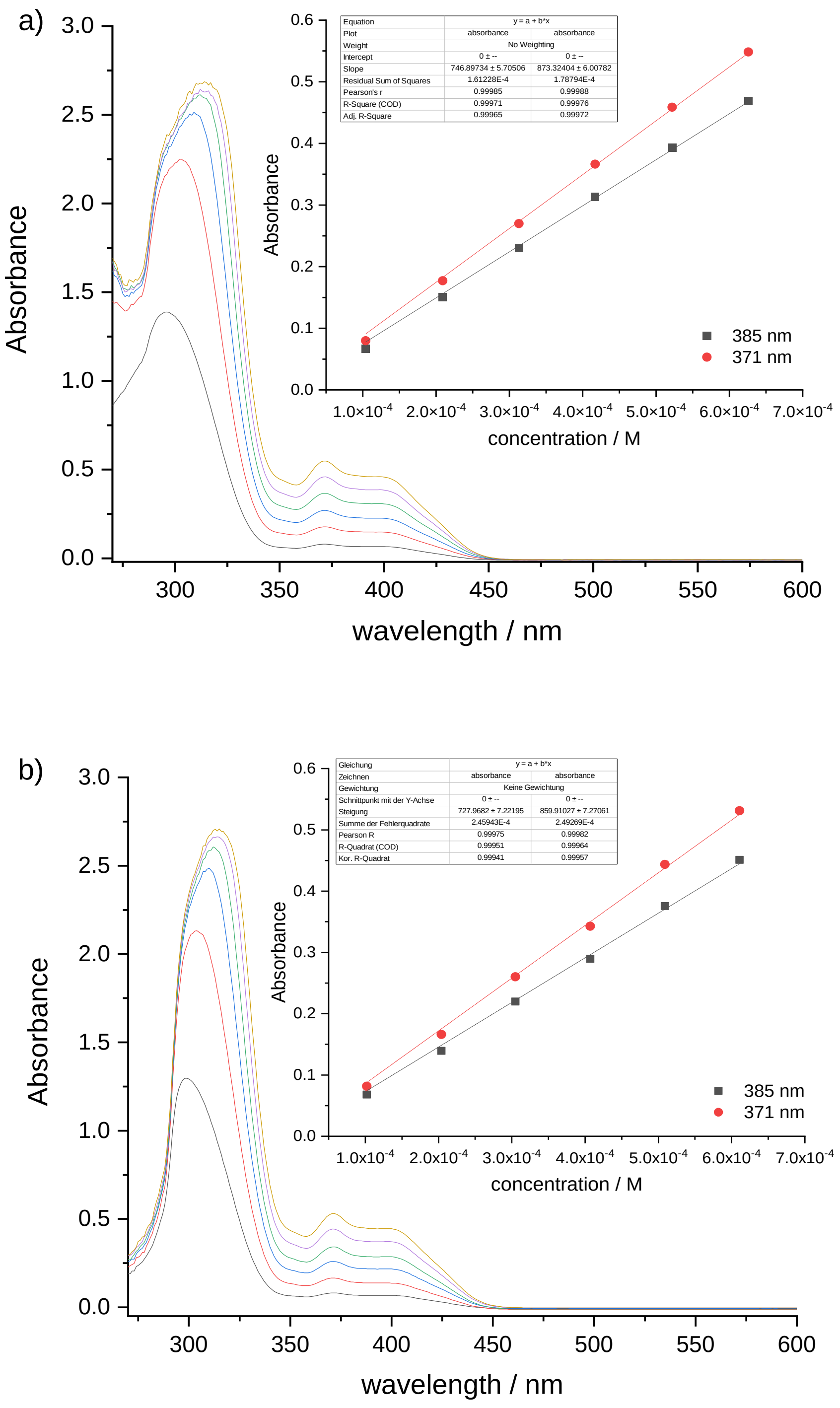

Figure S6. UV–vis spectra of 0.10 – 0.60 mM **1b** and fitted 385 nm and 371 nm absorbances for the determination of extinction coefficients (insert). a) in THF; b) in THF/MMA (1:1, v/v).

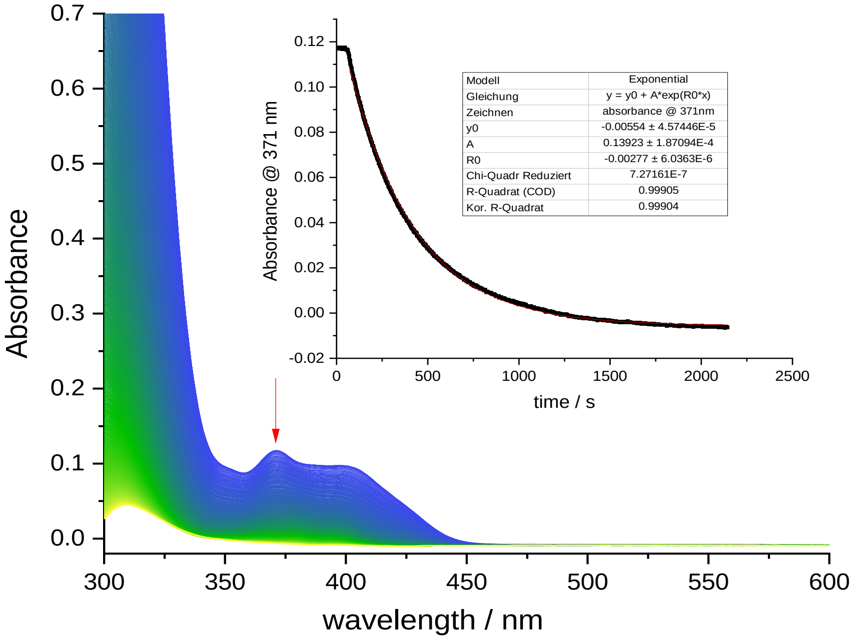

Figure S7. UV/Vis spectra of **1b** upon 385 nm LED irradiation, collected at 2-second intervals. The inset shows the decaying 371 nm absorbance maximum (red arrow) as a function of irradiation time. Conditions: 3 mL of 0.14 mM **1b** in THF/MMA (1:1, v/v), oxygen removed by bubbling with argon for 5 min.

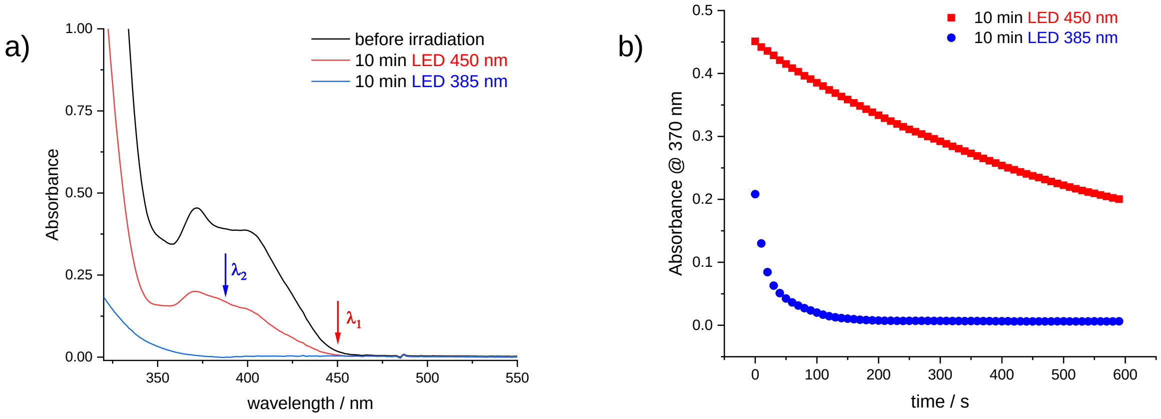

Figure S8. Stepwise photobleaching of **1b**. a) UV/Vis spectra of 0.38 mM **1b** in THF before irradiation (black trace), after 10 min of 450 nm LED irradiation (red trace), and after subsequent 10 min of 385 nm LED irradiation (blue trace). b) Time traces of the bleaching at the 371 nm absorbance maximum.

Omnirad® 819 - *Reference*

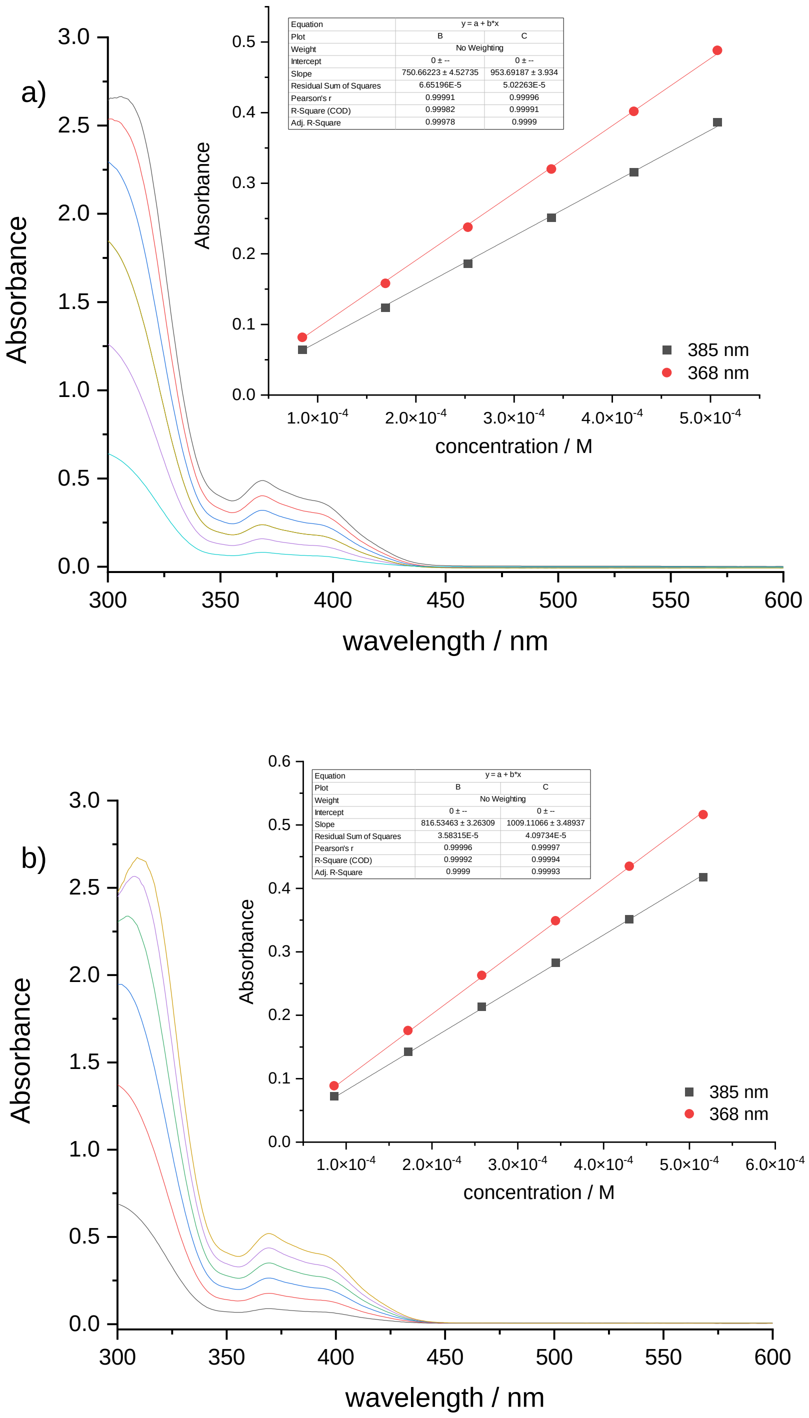

Figure S9. UV–vis spectra of 0.10 – 0.50 mM **Omnirad® 819** and fitted 385 nm and 368 nm absorbances for the determination of extinction coefficients (insert). a) in THF; b) in THF/MMA (1:1, v/v).

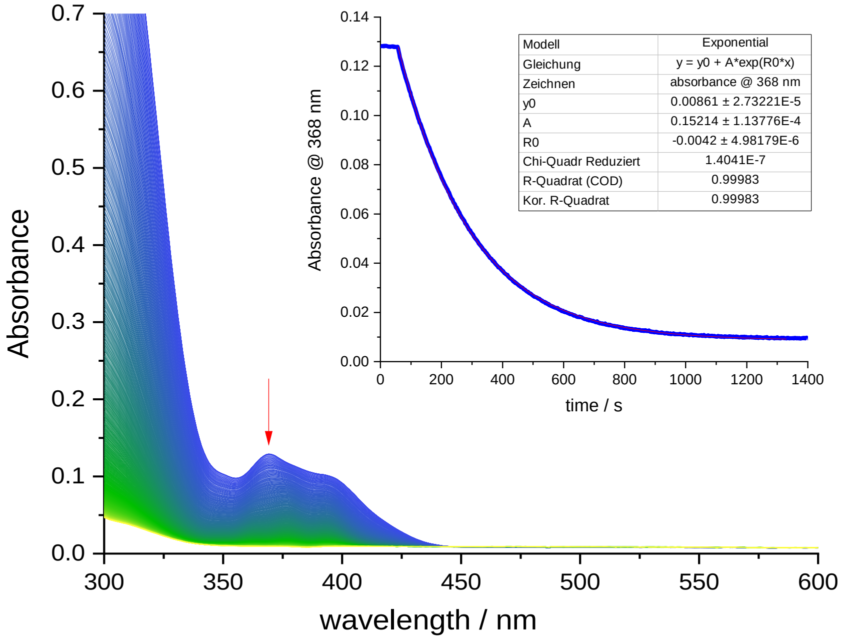

Figure S10. UV/Vis spectra of **Omnirad® 819** upon 385 nm LED irradiation, collected at 2-second intervals. The inset shows the decaying 368 nm absorbance maximum (indicated by the red arrow) as a function of irradiation time. Conditions: 3 mL of 0.14 mM **Omnirad® 819** in THF/MMA (1:1, v/v), oxygen removed by bubbling with argon for 5 min.

NMR-Spectroscopy

General Procedure. Solutions of 7 mM **1a** in tetrahydrofuran (THF-D_8) and benzene (C_6D_6) were prepared in conventional 5 mm NMR tubes and measured directly. NMR spectroscopy was performed on a 400 MHz Bruker Avance III System equipped with a Bruker H-D-X Z BBO probe. ^{13}Carbon spectra were recorded at 100.6 MHz with proton decoupling. ^{31}Phosphorus spectra were recorded at 161.9 MHz with and without proton decoupling. The reported chemical shifts are referenced against Tetramethylsilane (^{1}H and ^{13}C) or 80% H_3PO_4 (^{31}P) using the solvent lock signal as internal reference. All spectra were processed in MestReNova 15.1. Spectral simulations were performed employing the Modgraph NMR spin simulator implemented in MestReNova 15.1.

Procedure for Trapping of Phosphinoyl Radicals. ^{31}P-NMR spectroscopy was performed using 30 mM **1a** in the presence of 7 equiv. diphenyl disulfide ($(PhS)_2$) in $CDCl_3$ at 161.9 MHz on a Bruker Avance III System equipped with a QuadSystems X,F-H-D 05 PI BBO probe in conventional 5mm NMR tubes. During the first irradiation step, the samples were light-irradiated *in situ* inside the NMR probe for 10 min with 450 nm LEDs. The second irradiation step was performed *ex situ* with 385 nm LEDs for 10 min. Spectra were processed in MestReNova 15.1. and OriginPro 2021.

Procedure for Elucidating Heteronuclear Coupling. The alkyl-bridged bisacylphosphine oxide photoinitiators exhibit a ^{31}P NMR spectrum that is strongly influenced by coupling of the phosphorus nuclei to nearby protons in the alkyl chain. To map all relevant coupling interactions within the molecule, we employed heteronuclear and selective homonuclear decoupling on ^{1}H, ^{13}C and ^{31}P NMR spectra of **1a**. The experiments were performed in solvents with different dipole moments (THF-D_8 and C_6D_6). This is to show that the observed multiplicities are indeed a result of through-bond coupling, which is (typically) independent of the solvent. Soft pulses for ^{1}H homonuclear decoupling were calculated via the “selective 1D Expts” and “prohomodec” interface in TopSpin 3.6. Spin simulations were performed with the determined coupling constants and compared with the experimental spectra. The multiplicity of the proton-coupled ^{31}P resonance is assigned as a triplet of triplets (tt), appearing as pentet due to the experimental linewidth with an intensity ratio of 1:3:6:3: 1 (Figure S11).

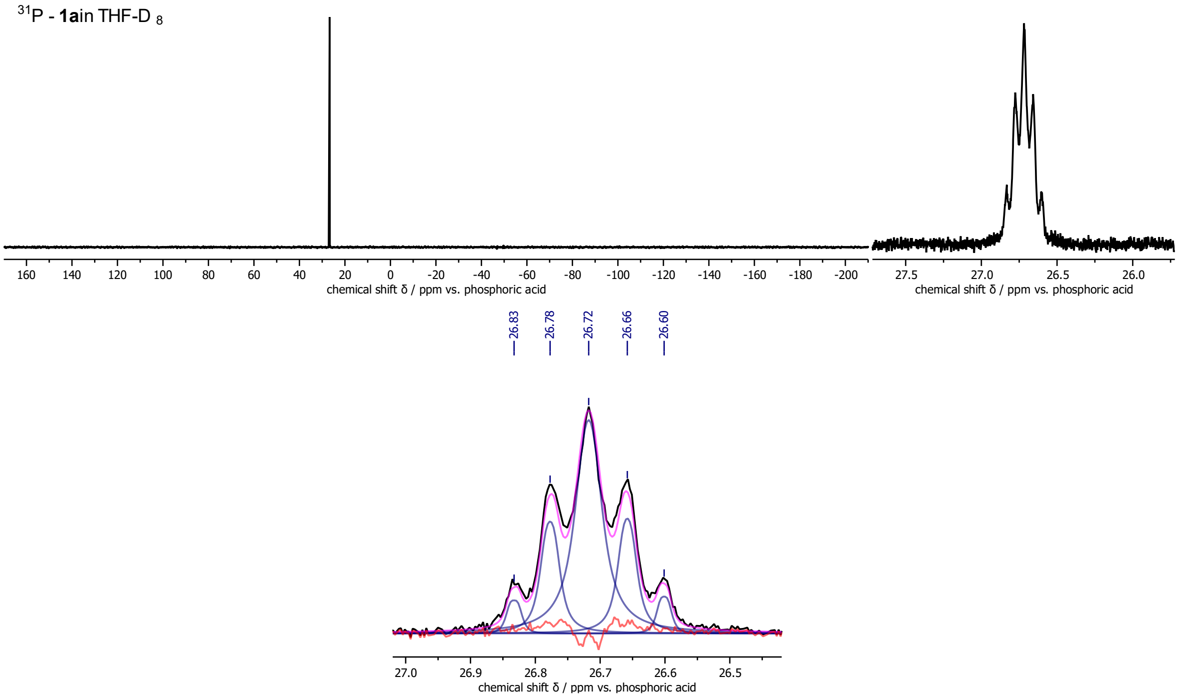

Figure S11. ^{1}H coupled ^{31}P NMR spectrum of **1a** (top left), magnified view of the 26.7 ppm resonance (top right) and spectral fit (bottom). Here, the black lines represent the experimental spectrum and blue lines represent the individual peak fits, whose sum gives rise to the purple (synthetic) spectrum. The orange line shows the fit-residues. Solvent: THF-D_8.

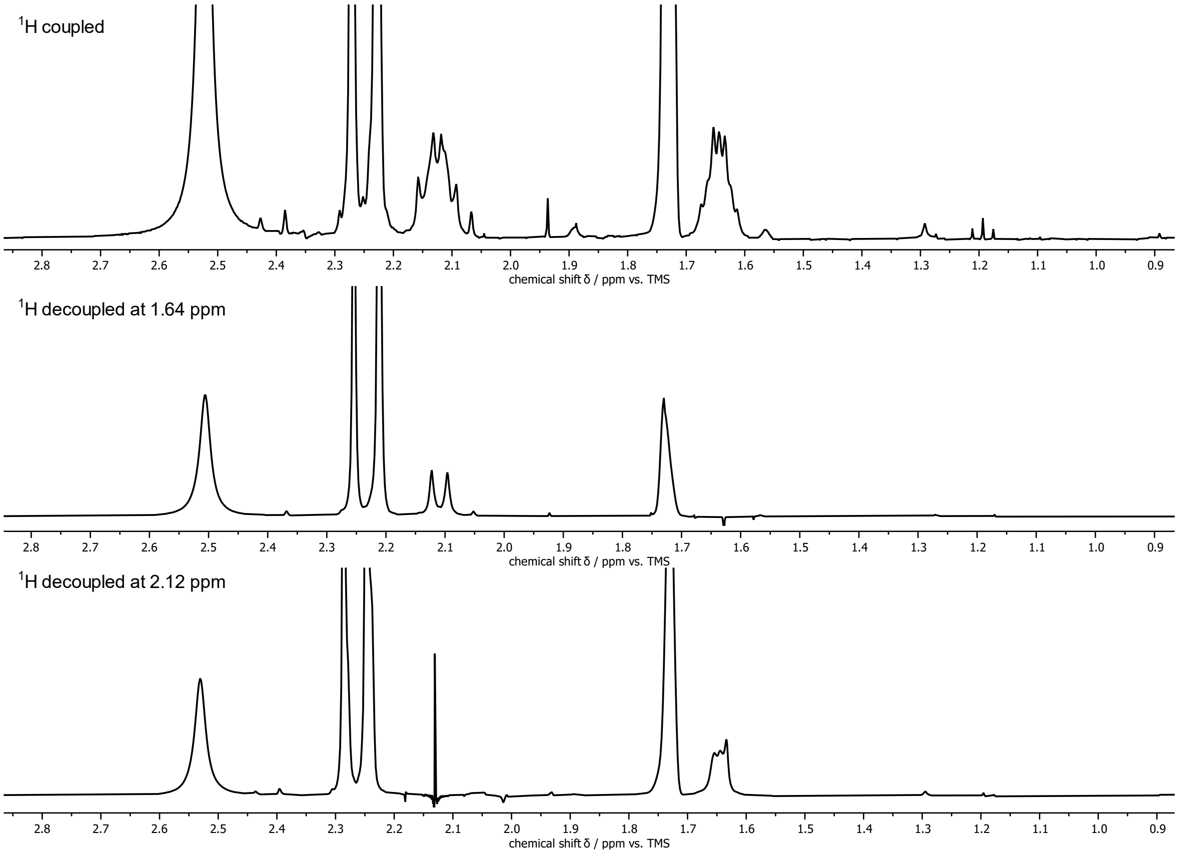

Figure S12. ^{1}H NMR spectrum of **1a** expanded around the alkyl peaks (top). Selective homo-decoupling is applied at 1.64 ppm (middle) and 2.12 ppm (bottom). From the splitting, the ^{31}P-^{1}H coupling constants are measured.

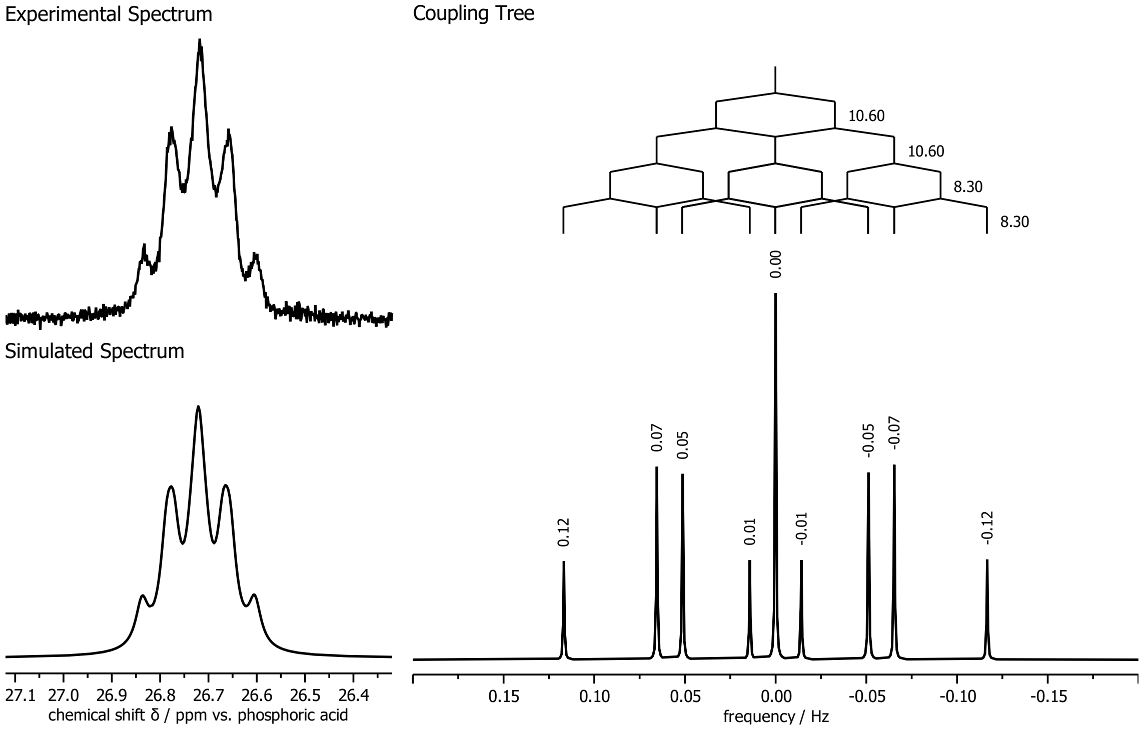

Figure S13. Experimental and simulated ^{1}H coupled ^{31}P NMR spectra (left) with corresponding coupling tree (right).

We have also investigated the ^{31}P-^{13}C couplings in **1a**. The corresponding coupling constants are readily determined from conventional proton-decoupled ^{13}C spectra. Figure S14 shows the resonances, for which coupling to ^{31}P is observed. The experimental coupling constants in Hz are $^{1}J_{CP}$ = 54.3 (G), $^{1}J_{CP}$ = 53.9 (H/K), $^{2}J_{CP}$ = 40.6 (F), $^{2}J_{CP}$ = 14.4 (I/J), $^{3}J_{CP}$ = 4.4 (I/J).

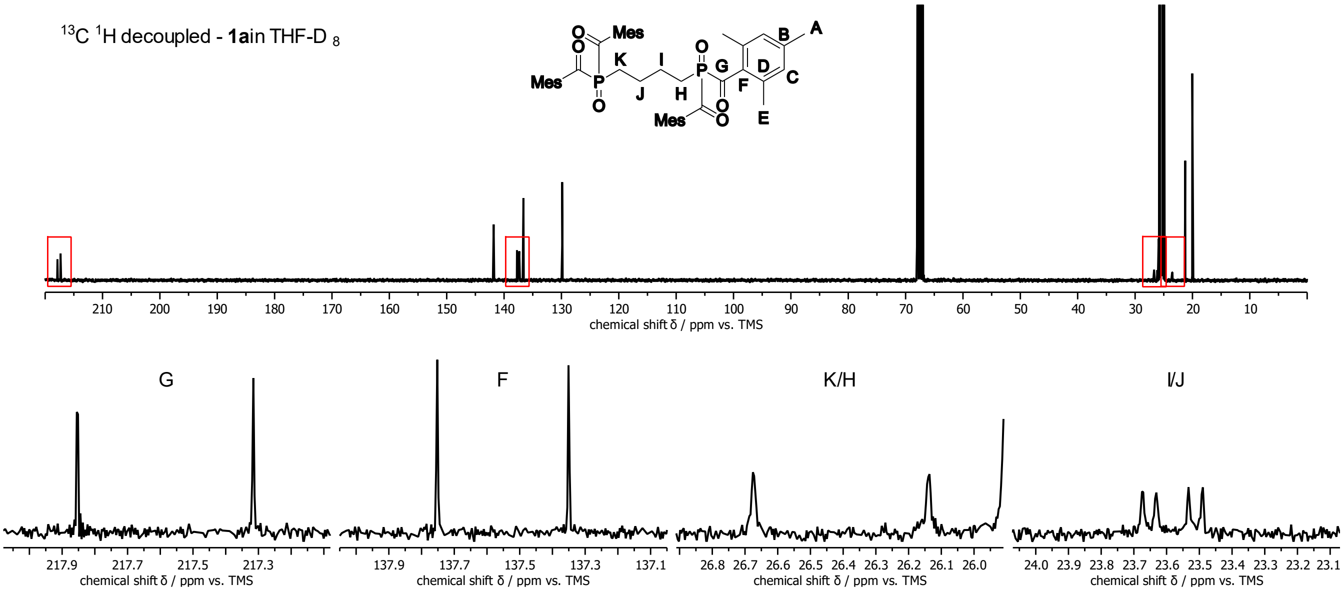

Figure S14. Proton-decoupled ^{13}C spectrum of **1a** (top). The resonances for which coupling to ^{31}P is observed are highlighted in red and magnified (bottom).

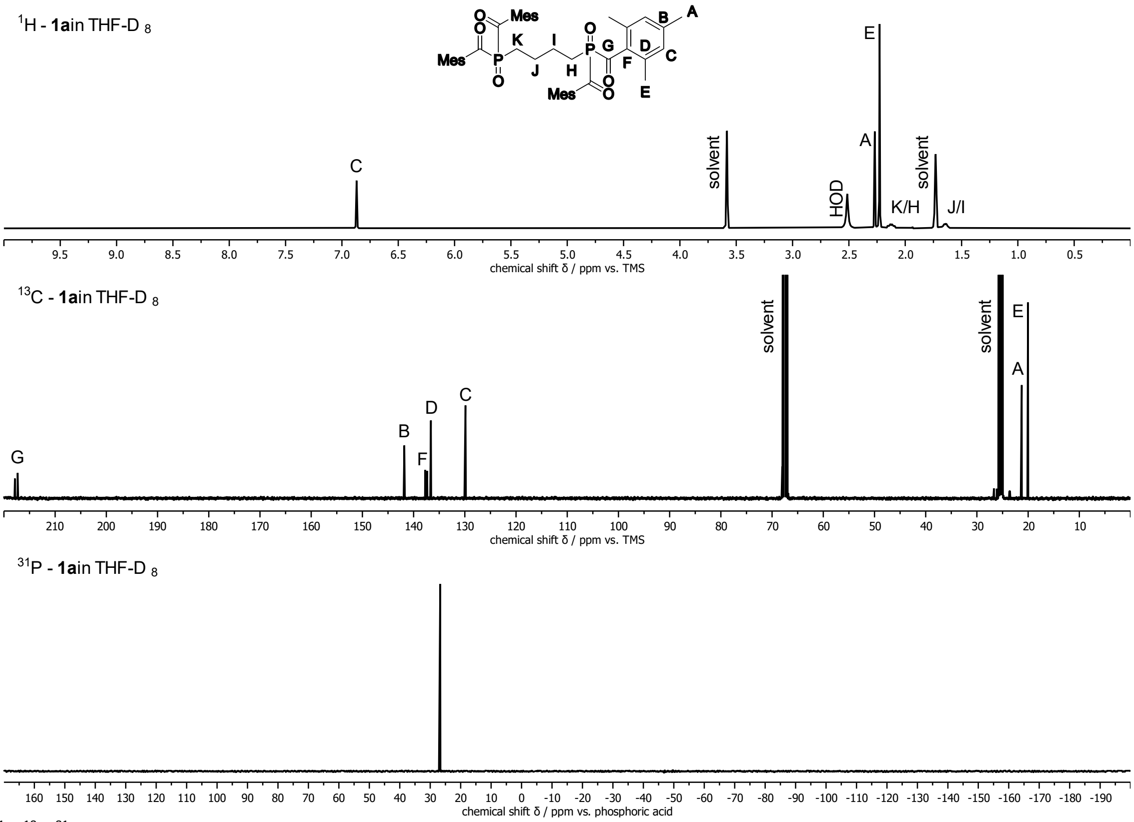

Figure S15. $^1H/^{13}C/^{31}P$-NMR spectra of **1a** in THF-D_8.

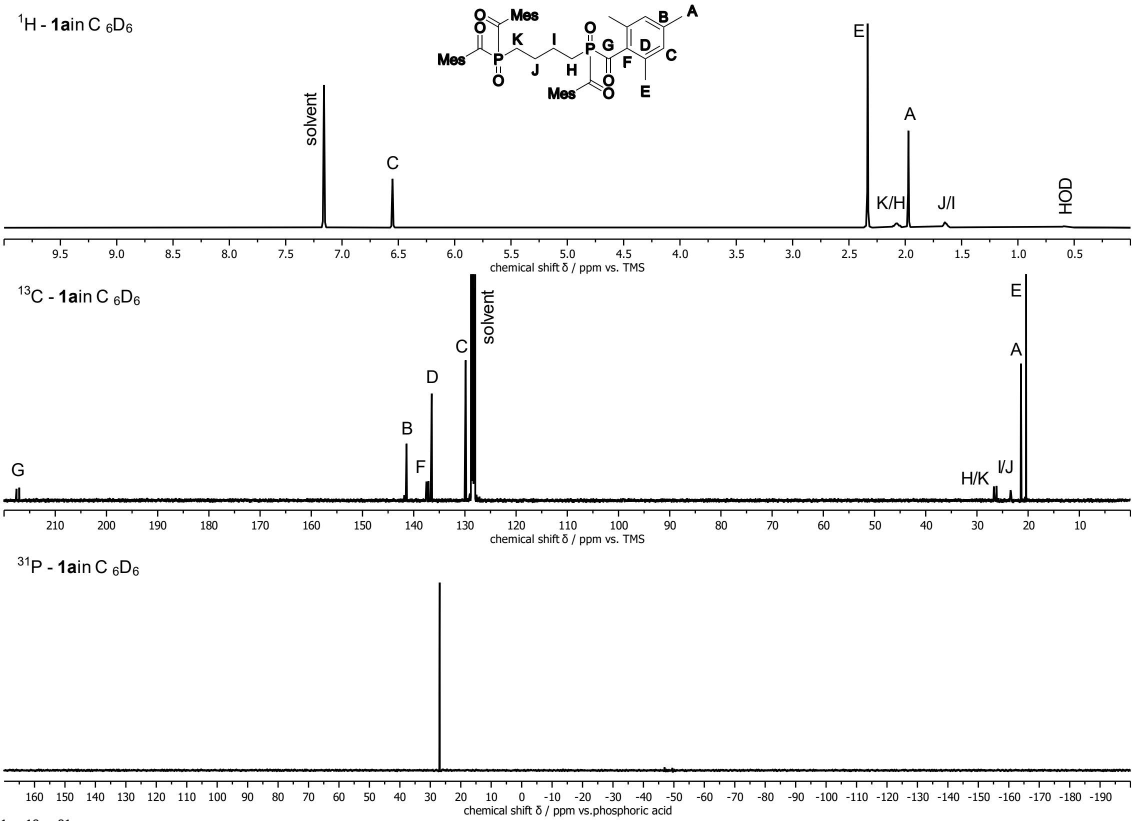

Figure S16. $^1H/^{13}C/^{31}P$-NMR spectra of **1a** in C_6D_6.

EPR-Spectroscopy

The EPR spin trapping measurements were performed on a Bruker EMX X-band spectrometer in tetrahydrofuran using 50 mM 5,5-dimethyl-1-pyrroline N-oxide (DMPO, Sigma-Aldrich; distilled prior to the application) as the spin trapping agent. The sample was irradiated directly inside the spectrometer during the measurement (40 scans) and the light source (450 nm or 385 nm LED) was turned on after the first scan was measured. The standard settings during EPR spin trapping experiments were: microwave frequency 9.751 GHz; 2.01 mW microwave power; sweep width 200 G; center field 3500 G; receiver gain 3.99×10^4; modulation amplitude 2 G; time constant 10.24 ms; sweep time 20.97 s. The experimental EPR spectra were processed and analysed using Bruker WinEPR, and the simulated spectra were calculated using Winsim 2002.

To confirm the selective stepwise α-cleavage mechanism, a combination of EPR spectroscopy and ^{31}P-NMR spectroscopy was employed. In the initial irradiation step, sample was exposed to 450 nm light in the presence of 5,5-dimethyl-1-pyrroline N-oxide (DMPO, 50 mM) in tetrahydrofuran (THF). The resulting EPR spectrum (Figure S17) exhibits a characteristic pattern corresponding to the DMPO spin adduct of the mesitoyl radical, as determined by spin Hamiltonian parameters obtained from simulation analysis (hyperfine coupling constants: α_N = 15.9 G, α_H = 13.8 G). For the second irradiation step, the sample was first subjected to *ex situ* bleaching with 450 nm light for 10 minutes in the absence of DMPO to prevent premature spin adduct formation. Following this treatment, DMPO was added at a concentration of 50 mM, and the sample was irradiated *in situ* using a 385 nm LED source. Under these conditions, EPR analysis revealed the formation of two distinct paramagnetic species. The dominant signal, comprising five prominent lines, corresponded again to the DMPO–mesitoyl radical adduct, now with slightly shifted hyperfine coupling constants (α_N = 14.97 G, α_H = 13.30 G). In addition, a second, less intense signal was observed, belonging to a phosphorus-centred radical adduct characterized by hyperfine couplings of α_P = 38.5 G, α_N = 14.5 G, α_H = 18.0 G.

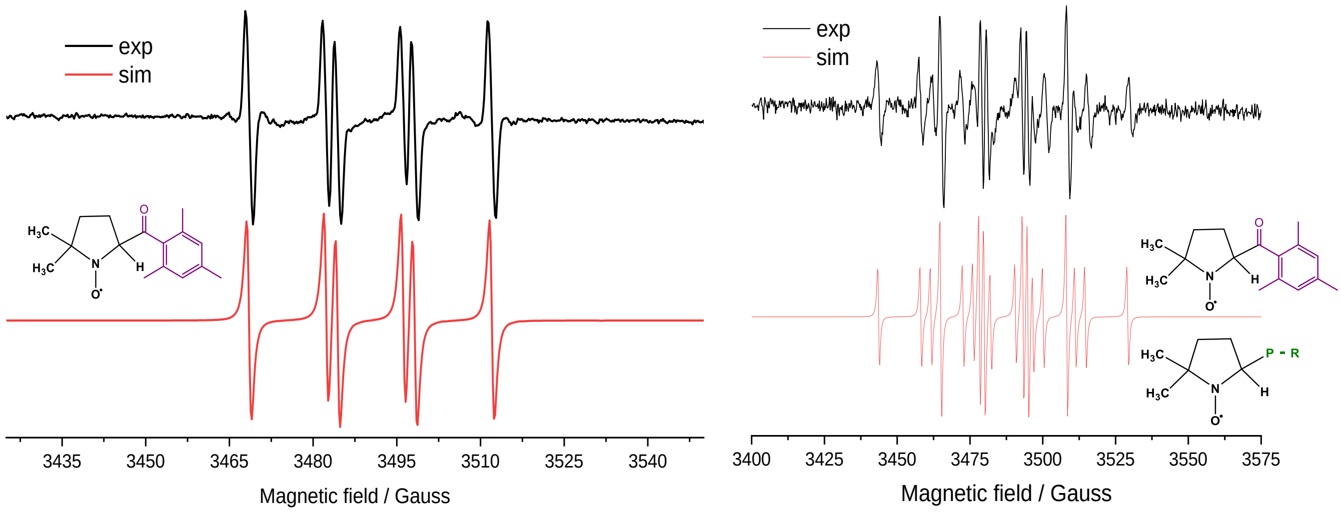

Figure S17. Left: Experimental (–) and simulated (–) EPR spectra acquired after irradiation with a 450 nm LED. Initial concentrations: c_0 (**1b**) = 5 mM; c_0(DMPO) = 50 mM dissolved in THF. Right: EPR spectrum of 2 trapped radical species after the irradiation of **1b**-MAPO.

Synthesis of Polymers

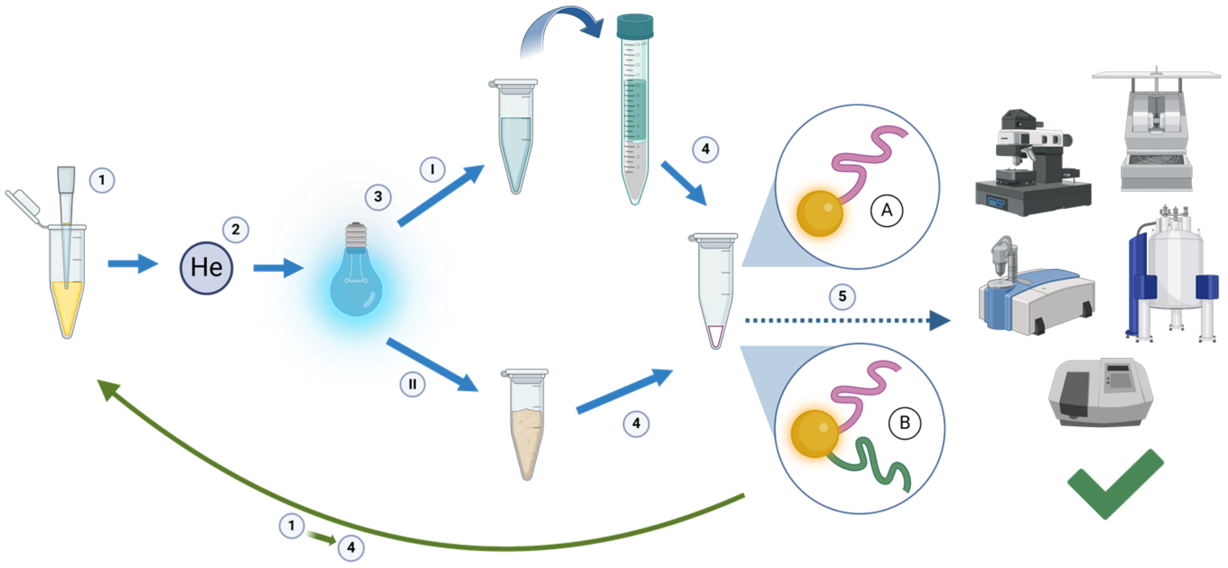

Figure S18. Scheme illustrating the preparation of polymers (created with BioRender.com).

The stepwise polymerization procedure is illustrated in Figure S18. The concentrations and volumes of reactants, as well as the irradiation times and intensities, were adjusted for each monomer to ensure the formation of a solid-state adduct that remains soluble in either hydrophilic (e.g., water) or hydrophobic (e.g., tetrahydrofuran (THF) or chloroform) solvents for further analysis. One-arm (pMMA, pAA), cross-linked (pMMApMMA, pAApAA), and star-shaped (pMMApBz, pAApAc, pMMApAA, pMMApAc) polymers were synthesized via stepwise bulk polymerization using **1b** as the photoinitiator. Since the photoinitiator is insoluble in the monomers used, it was prepared as a 10 mM solution in THF. The synthesis procedure consisted of four main steps:

1. **Preparation of the reaction mixture**: A mixture of photoinitiator, monomer, and solvent was prepared in either a glass vial or an Eppendorf tube. The specific amounts and concentrations for each monomer are summarized in Table S6.
2. **Degassing**: The mixture was degassed with helium for 5 minutes to remove oxygen, which could otherwise inhibit the polymerization process.
3. **Stepwise irradiation**: The sample underwent two sequential polymerization cycles: the first cycle involved irradiation with a 450 nm LED for 10 minutes, followed by a second cycle using a 378 nm LED for 2–10 minutes.
    - When a hydrophobic monomer such as MMA or BzMA was used, the resulting one-arm pMMA polymer was precipitated in 7 mL of cyclohexane, while two-arm polymers (pMMApMMA and pMMApBz) were precipitated in 3–4 mL of methanol.
    - If a hydrophilic monomer was used, precipitation was not required, yielding a honey-like or mash-like solid product.
4. **Drying**: In both cases, the resulting polymer was air-dried to obtain the final product.

Table S6. Summary of the preparation condition of different one-arm, cross-linked and star-shaped polymers.

Polymer Acronym	*Volume of 10 mM 1b-PI*	*Amount of polymer from step 1*	*Amount of Monomer*	*Volume of solvent*	*Bleaching time / min*	*Product soluble in*
pMMA	200 µl	-	900 µl MMA	900 µl THF	10	THF, $CHCl_3$
pMMApMMA	-	10 mg pMMA	200 µl MMA	200 µl THF	10	THF, $CHCl_3$
pMMApBzMA	-	10 mg pMMA	200 uL BzMA	200 µl THF	30	$CHCl_3$
pAA	100 µl	-	20 mg AA	400 µl THF	10	H_2O
pAAc	100 µl	-	100 µl AAc	100 µl THF	10	H_2O
pAApAA	-	5 mg pAA	20 mg AA	300 µl H_2O	5	H_2O
pAApAAc	-	5 mg pAA	50 µl AAc	200 µl H_2O	3	H_2O
pMMApAA	-	10 mg pMMA	20 mg AA	400 µl THF	10	-
pMMApAAc	-	5 mg pMMA	300 µl AAc	200 µl THF	2	H_2O

The optical and electron paramagnetic resonance (EPR) spectra of **1b**, poly(methyl methacrylate) (pMMA) homopolymer, and crosslinked pMMA (pMMApMMA) polymer, as presented in Figure S19 provide clear evidence of the successful stepwise α-cleavage process. Upon irradiation at 450 nm, the formation of DMPO-mesitoyl radical adducts was observed using EPR spin-trapping, indicating the cleavage of the first mesitoyl moiety. Concurrently, a significant decrease in the 370 nm absorption maximum of **1b** was detected in the UV-vis spectrum, confirming photobleaching. Notably, exposure of the pMMA homopolymer to the same 450 nm irradiation did not generate any detectable EPR signal, as this wavelength lacks the requisite energy to cleave the second mesitoyl moiety in the **1b**-MAPO-pMMA structure. Subsequent irradiation at 378 nm facilitated the cleavage of the second mesitoyl moiety, as evidenced by the reappearance of DMPO-mesitoyl radical adducts in the EPR spectrum as well as the same P-centered DMPO radical adduct observed by the bleaching of **1b** in Figure S19.

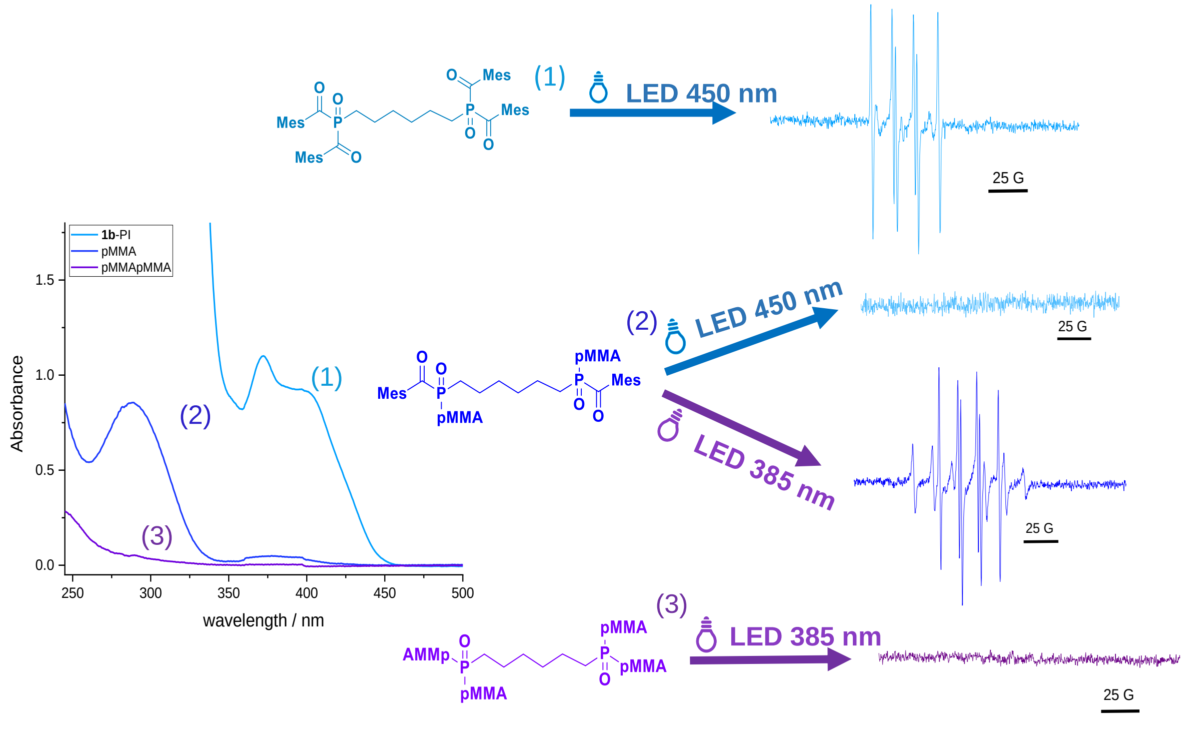

Figure S19. Left: UV-vis spectra of **1b** (light-blue trace), pMMA homopolymer (dark-blue trace) and pMMApMAA crosslinked polymer (purple trace) in THF. Right: EPR spectra of the stepwise cleavage of mesitoyl moiety and formation of DMPO-mesitoyl radical adduct in THF.

Bleaching of the **1b**-pMMApMMA crosslinked polymer using a 385 nm LED initially produces no EPR signal, as the mesitoyl moiety in the photoinitiator has already been cleaved and is no longer present. However, over time, a new DMPO adduct signal appears (Figure S18). This product corresponds to a DMPO–peroxyl radical adduct (DMPO–OO•), characterized by hyperfine splitting constants of α_N = 12.98 G and α_H = 8.33 G. The primary radical (R•), generated via backbone cleavage of the polymer upon 385 nm irradiation of **1b**-pMMApMMA, reacts with molecular oxygen to form a peroxyl radical (ROO•). This ROO• is subsequently trapped by DMPO, resulting in the observed EPR signal. The proposed reaction pathway is summarized as follows:

1b-pMMApMMA → R• + O_2 → ROO• → DMPO–OO• (3)

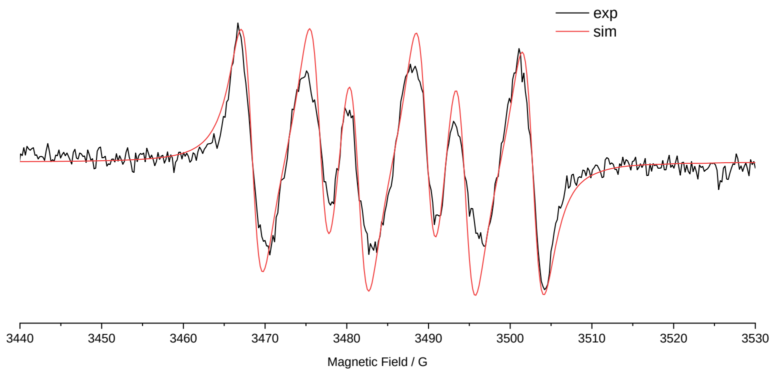

Figure S20. Experimental (–) and simulated (–) EPR spectra monitored after irradiation of **1b**-pMMApMMA polymer with 385 nm LED in THF in the presence of 50 mM DMPO. Spectrum shows DMPO–OO• with α_N = 12.98 G and α_H = 8.33 G.

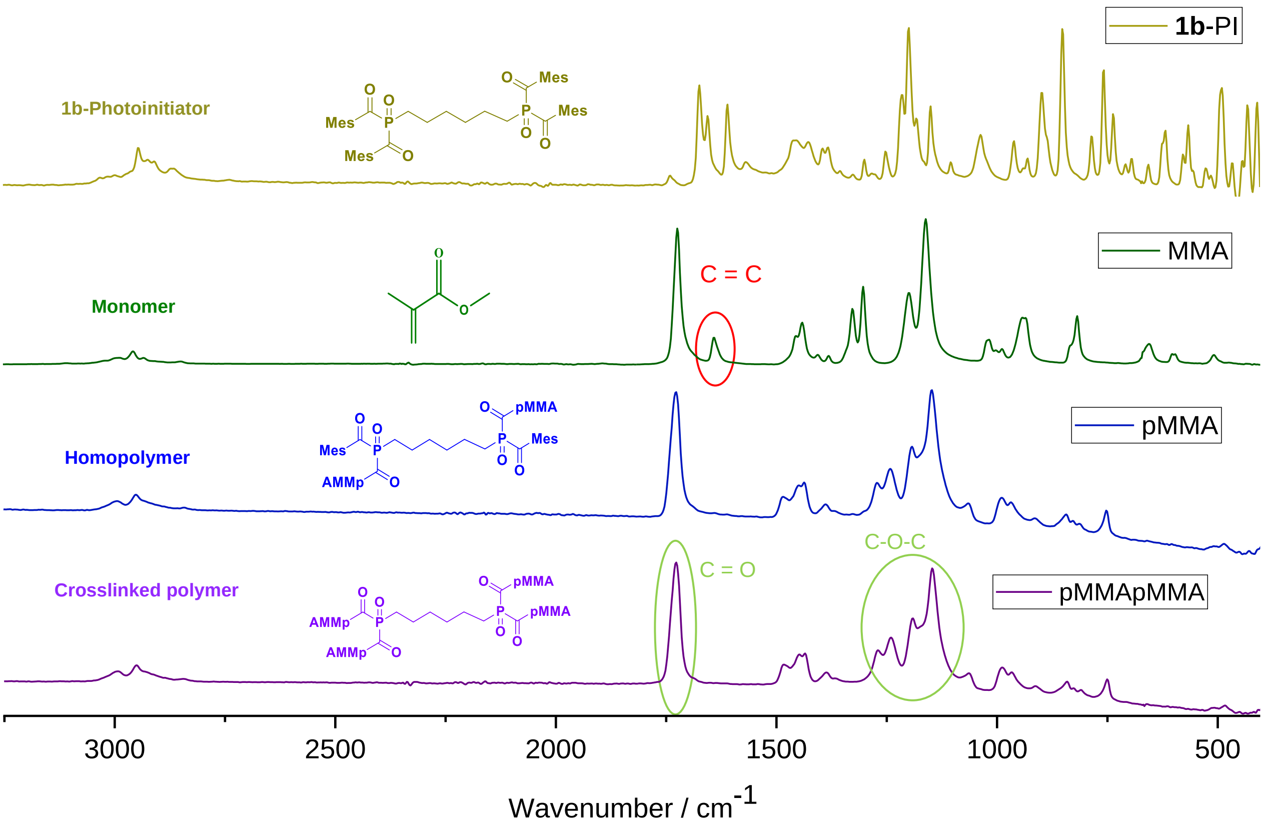

Figure S21. IR spectra of **1b** (yellow trace), metylmethacrylate monomer (green trace), **1b**-pMMA (dark-blue trace) and **1b**-pMMApMAA (purple trace).

The polymerization of methyl methacrylate into polymethylmethacrylate was confirmed using IR spectroscopy (Figure S21) and ^{1}H-NMR spectroscopy (Figure S22). FT-IR spectra in solid state were recorded on a Bruker Alpha spectrometer in absorption mode using OPUS 7.5 software. Spectra were processed in OriginPro 2021. The ^{1}H-NMR spectra of the prepared homo (**1b**-pMMA) and crosslinked (**1b**-pMMApMMA) polymers, as well as the unpolymerized **1b** and MMA (10 equiv.) mixture were measured on a 200 MHz Bruker Avance II Spectrometer in THF-D_8. The characteristic C=C stretching vibration at 1636 cm^{-1}, indicative of the vinyl functional groups in the monomer, was absent in the polymer spectrum. In contrast, the strong carbonyl (C=O) absorption band at 1721 cm^{-1} and the C-O-C stretching vibrations in the 1144–1190 cm^{-1} range, corresponding to the ester functionality, remained present with slight shifts, confirming the retention of the ester moiety in the polymer structure.

^{1}H-NMR analysis further supported the polymerization process. The two vinyl proton signals (CH_2=C) at δ = 5.5 and 6.0 ppm, characteristic of MMA, were significantly reduced in intensity in the polymer spectrum, confirming the consumption of the monomer. Similarly, the aromatic proton signal at δ = 6.7 ppm, attributed to the mesitoyl moiety in the **1b** structure (Figure S22), exhibited a marked decrease after the bleaching process. Conversely, the methoxy (-OCH_3) signal at δ = 3.6 ppm remained unchanged, indicating the preservation of the ester functional group. The broadening of the proton signals in the polymer spectrum was attributed to the increased molecular weight of pMMA and the formation of high-molecular-weight polymer chains. These spectral changes confirm the successful polymerization of MMA and the structural modifications associated with the crosslinking process. The results demonstrate a controlled, wavelength-selective photoinitiation and polymerization process, facilitating the stepwise construction of crosslinked polymer networks with precise radical generation.

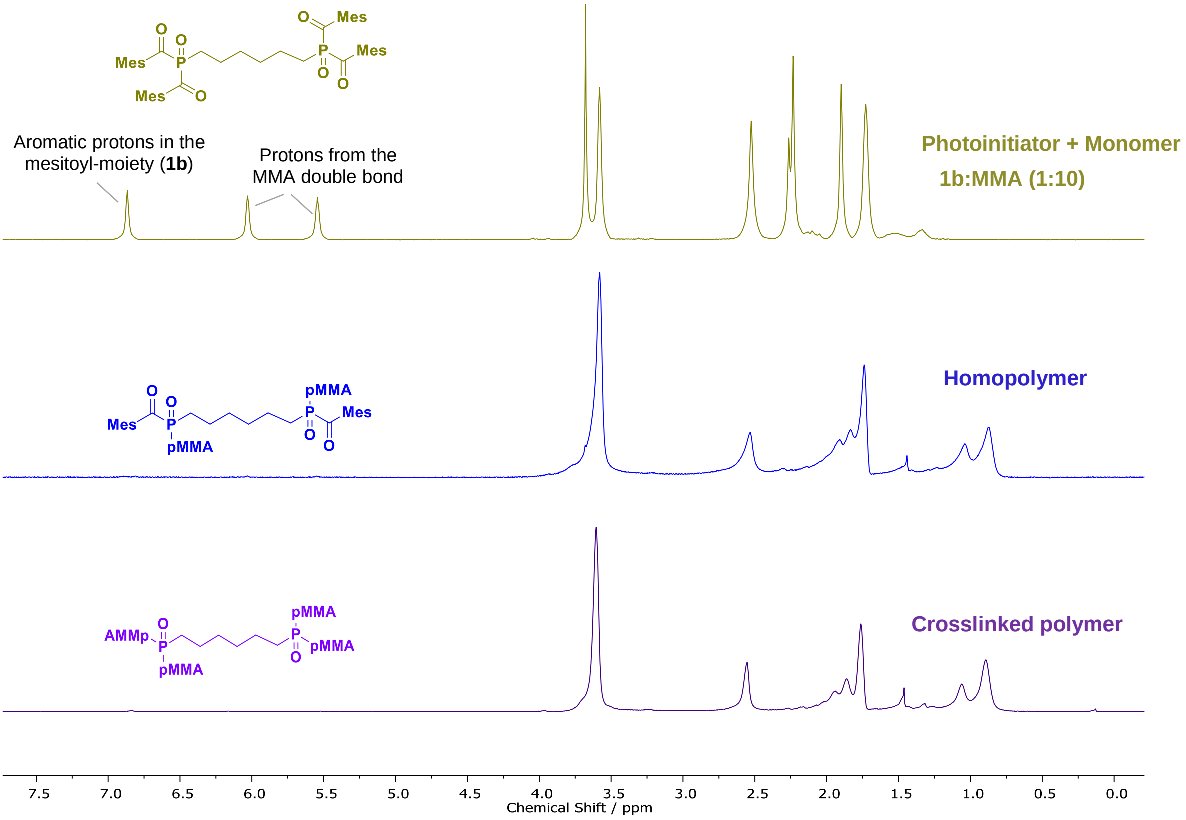

Figure S22. ^{1}H NMR spectra of a solution of **1b** and 10 eq. MMA in THF-D_8 (top, yellow trace), the corresponding **1b**-pMMA homopolymer (middle, dark-blue trace), and the crosslinked **1b**-pMMApMAA polymer (bottom, purple trace).

Atomic Force Microscopy

Polymeric thin films were prepared by spin coating onto silicon substrates. A 5 µL aliquot of a 1% (w/v) polymer solution, dissolved in either tetrahydrofuran, chloroform, or water, was deposited onto 1 × 1 cm silicon plates and left to dry in air to form thin films. AFM measurements were performed using an Asylum Research Cypher ES atomic force microscope (Oxford Instruments) in tapping mode equipped with blueDrive photothermal excitation. The images were acquired in air, at room temperature and at a scanning rate of 2 Hz, using Budgetsensors Tap300-G silicon probes. AFM images were collected on different regions to ensure they are representative of the samples. Topography images were subjected to a linear filtering.

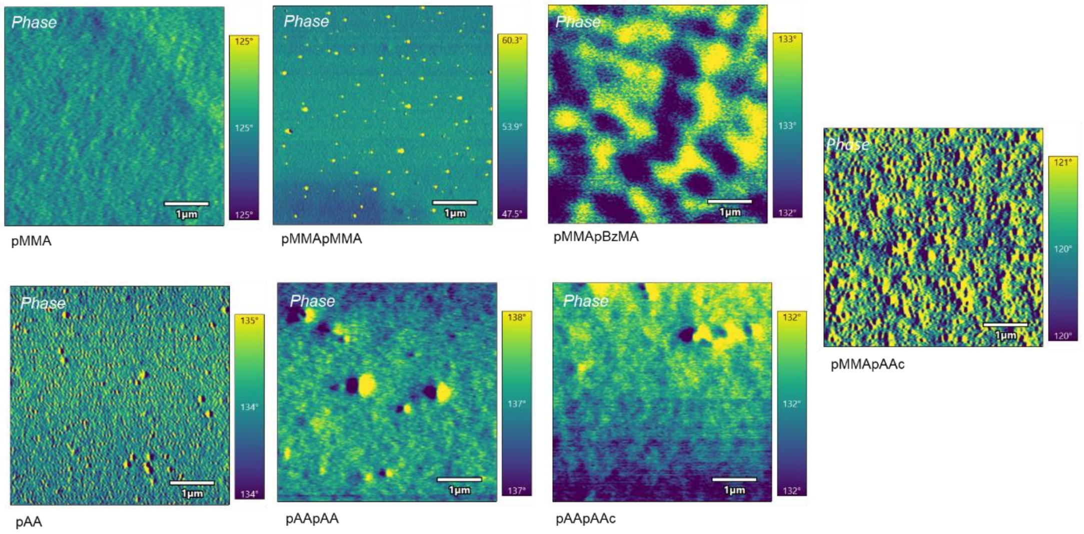

Figure S23. 5x5 micron AFM images of the phase retrace (ColorMap: Viridis).

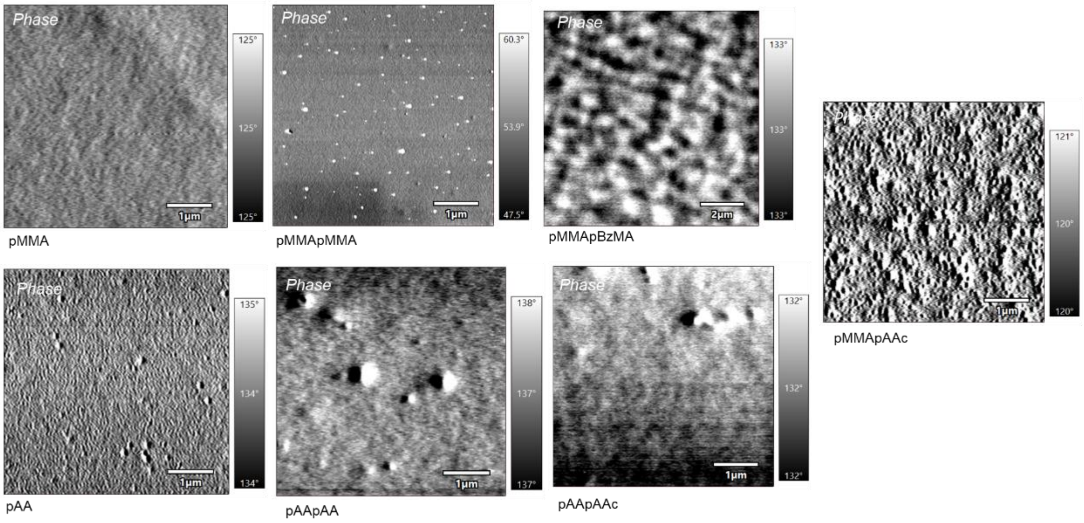

Figure S24. 5x5 micron AFM images of the phase retrace (raw data).

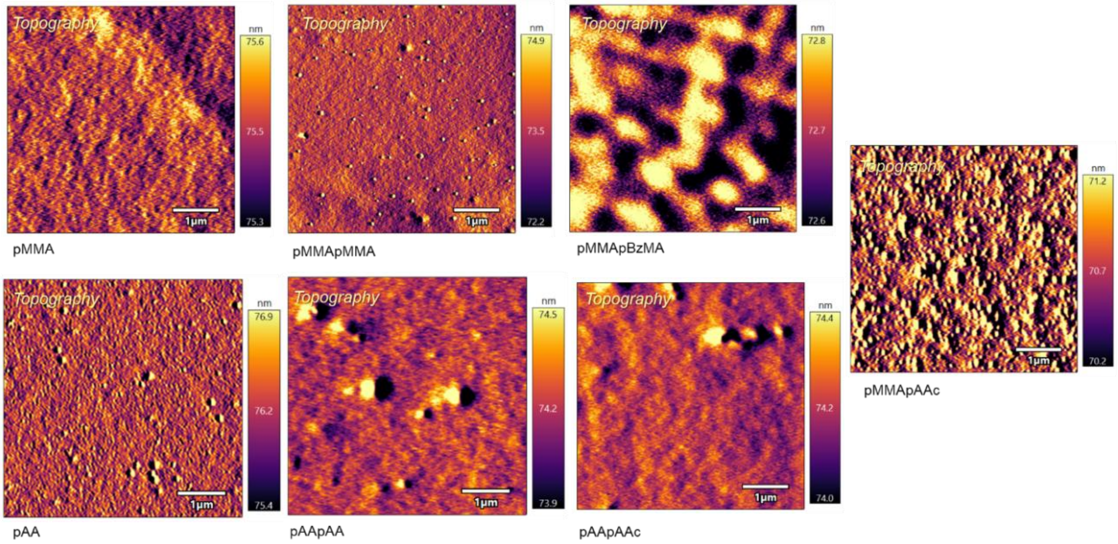

Figure S25. 5x5 micron AFM images of the topography (ColorMap: Inferno).

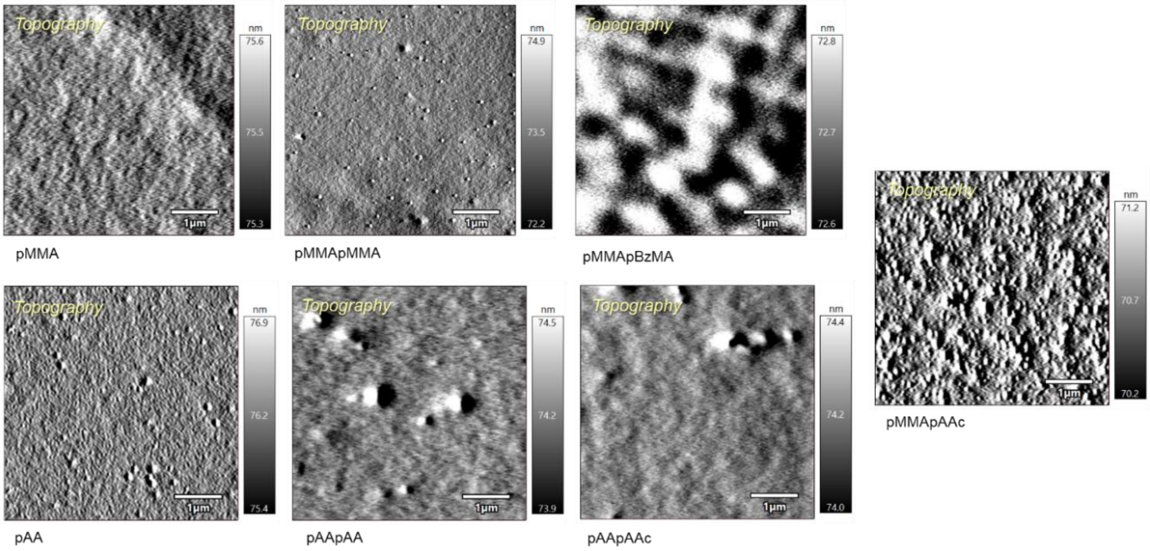

Figure S26. 5x5 micron AFM images of the topography (raw data).

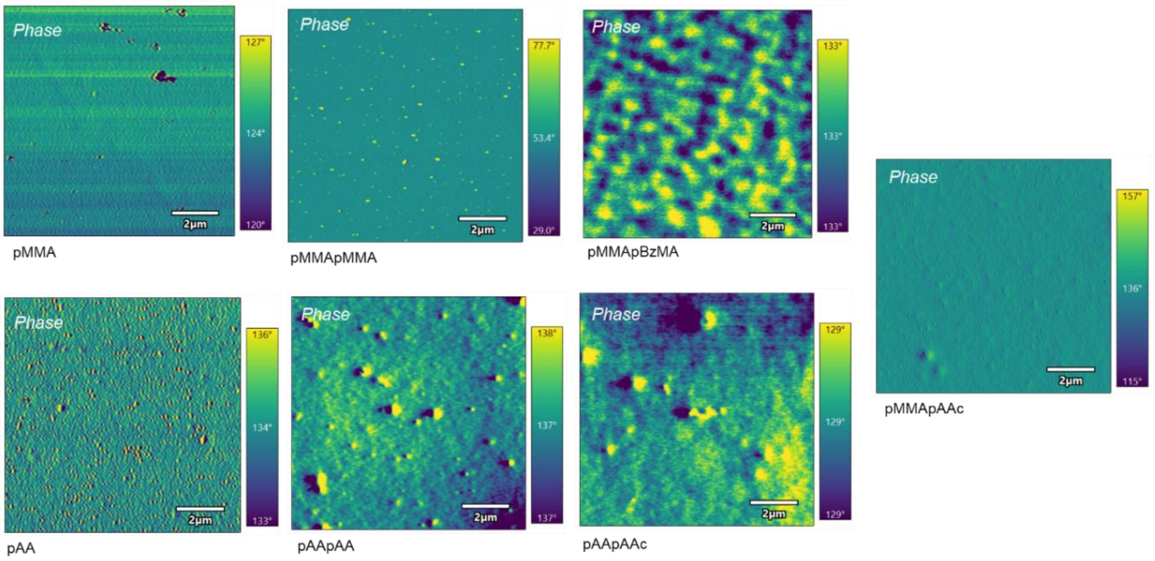

Figure S27. 10x10 micron AFM images of the phase retrace (ColorMap:Viridis).

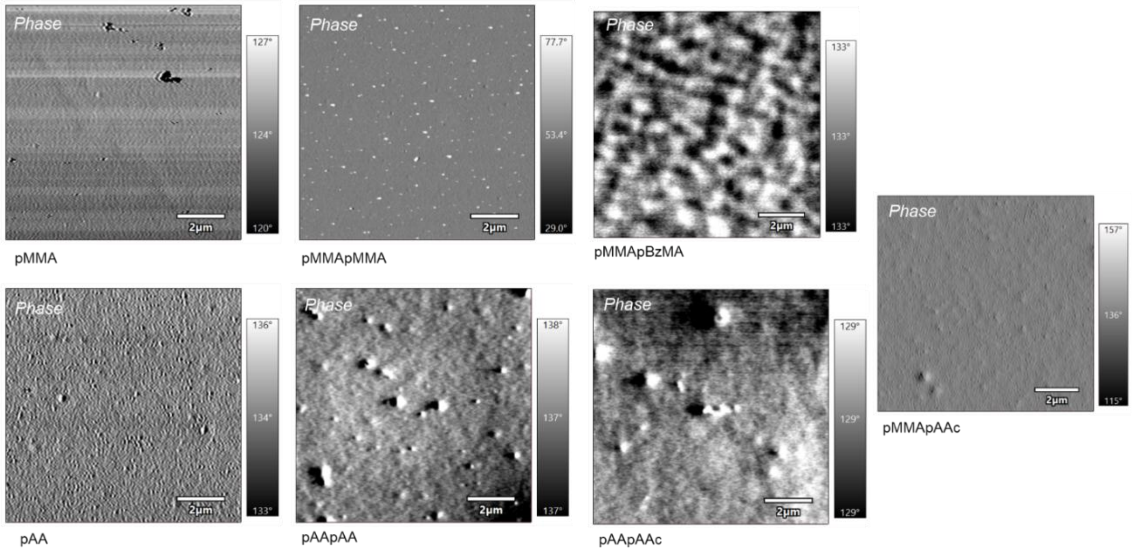

Figure S28. 10x10 micron AFM images of the phase retrace (raw data).

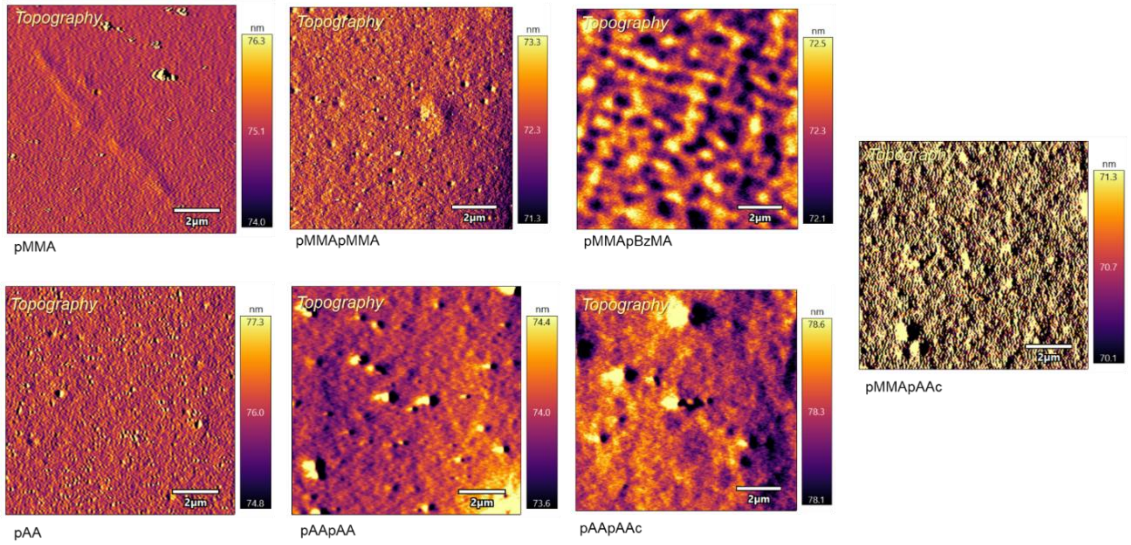

Figure S29. 10x10 micron AFM images of the topography (ColorMap:Inferno).

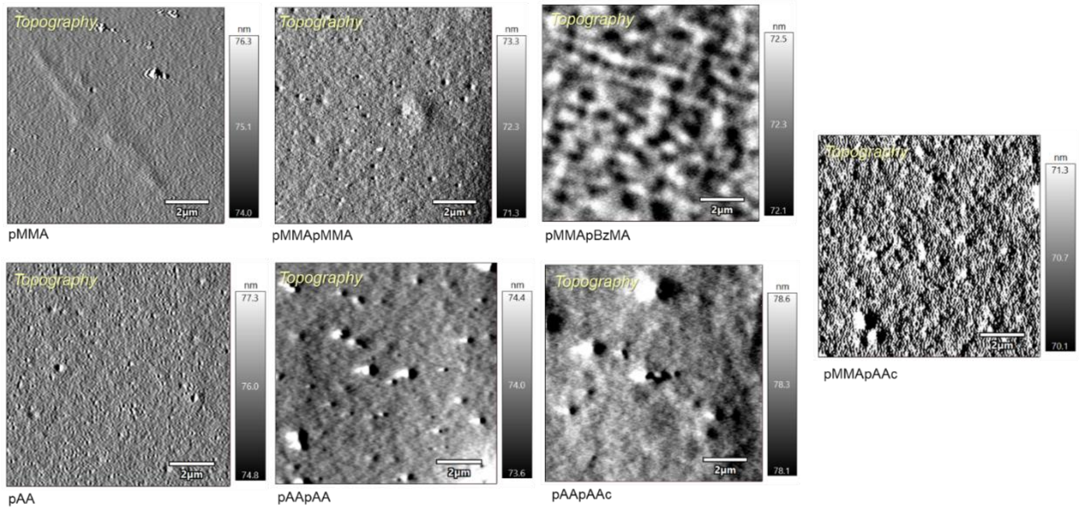

Figure S30. 10x10 micron AFM images of the topography (raw data).

Table S7. Summary of the RMS Roughness on 5 µm x 5 µm areas (pm) of the polymers.

Polymer	*RMS Roughness on 5 µm x 5 µm areas (pm)*
pMMA	372
pMMApMMA	924
pMMApBz	581
pAA	323
pAApAA	437
pAApAc	434

Contact Angle Measurements

Contact angle measurements were performed using a Krüss DSA100 drop shape analyzer (Krüss GmbH, Hamburg, Germany) equipped with a high-resolution camera and analysis software. Polymeric thin films were prepared by spin coating onto silicon substrates using 60 µL aliquot of a 1% (w/v) polymer solution, dissolved in either tetrahydrofuran, chloroform, or water and then deposited onto 3 × 2 cm silicon plates and left to dry in air to form thin films. A sessile drop method was employed, wherein 2 µL droplets of diiodomethane or deionized water were gently deposited on the sample surface using a micro-syringe. Measurements were taken at room temperature under ambient conditions. For each sample 3 independent measurements were taken at different locations. The contact angle was measured at both left and right edges of the droplet, and the average value was used.

Table S8. Determined contact angles for diiodomethane and water. The last row reports the average angles and corresponding standard deviation for each polymer.

**Diiodomethane drop**			**Water drop**		
pMMApBzMA	pMMApAAC	pAApAAc	pAApAAc	pMMApAAc	pMMApBzMA
36.2	46.0	47.8	61.0	73.2	79.4
38.0	44.9	46.4	61.3	73.8	73.6
36.3	43.5	46.8	62.0	77.2	78.2
36.9 ± 0.6	44.8 ± 0.7	47.0 ± 0.4	61.4 ± 0.3	74.7 ± 1.3	77.1 ± 1.8

NMR-Spectra

Compound 1a

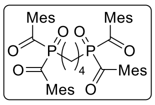

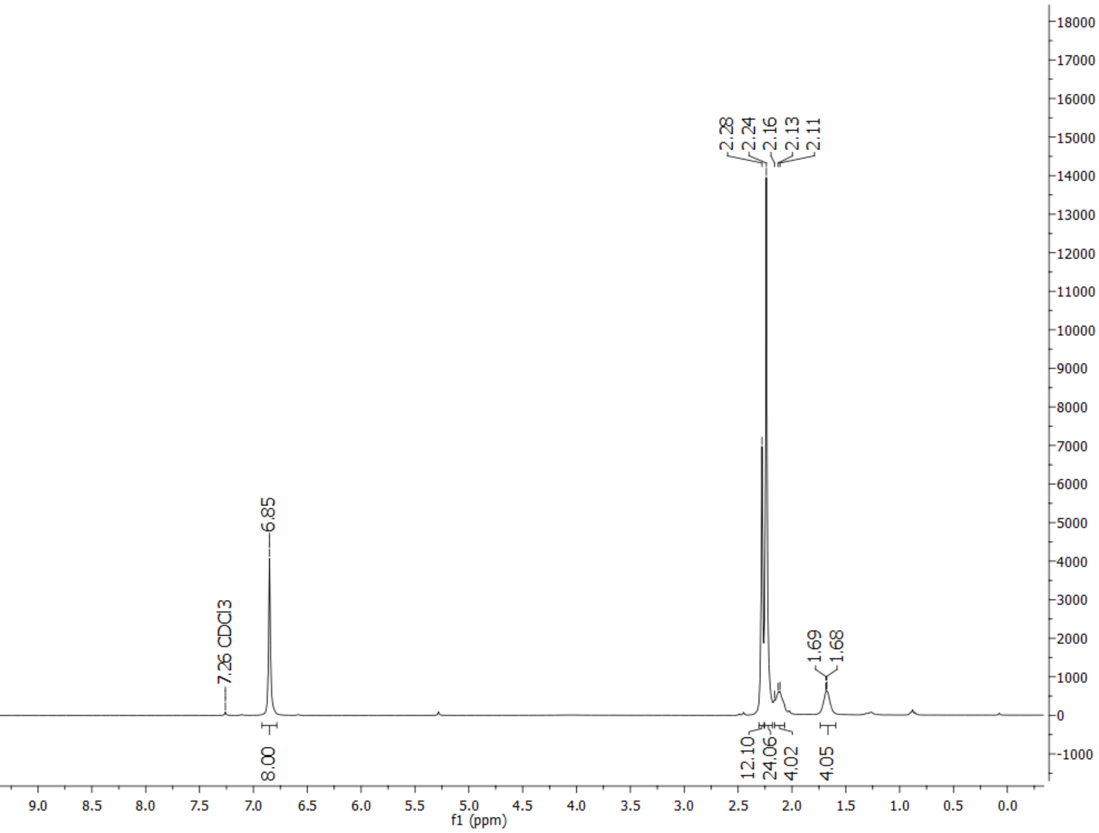

Figure S31. ^{1}H-NMR (300 MHz, $CDCl_3$) spectrum of **1a**.

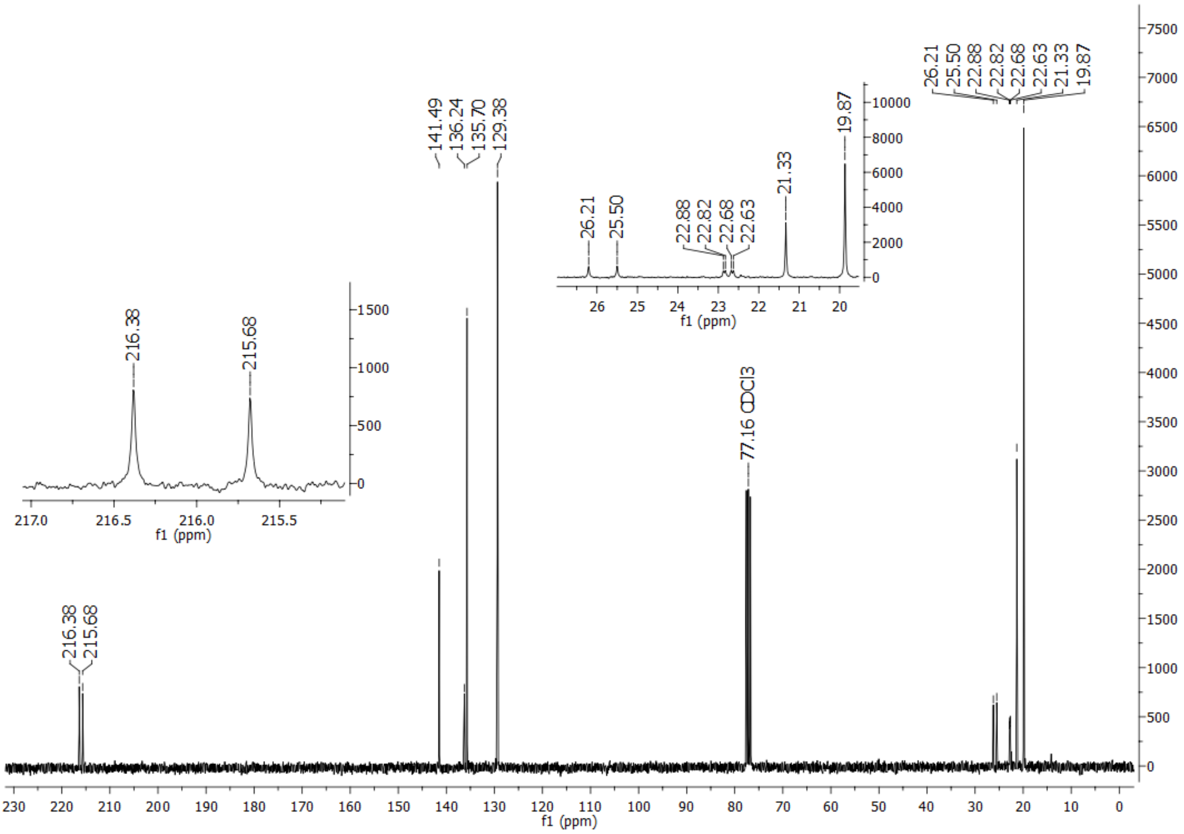

Figure S32. ^{13}C-NMR (75 MHz, $CDCl_3$) spectrum of **1a**.

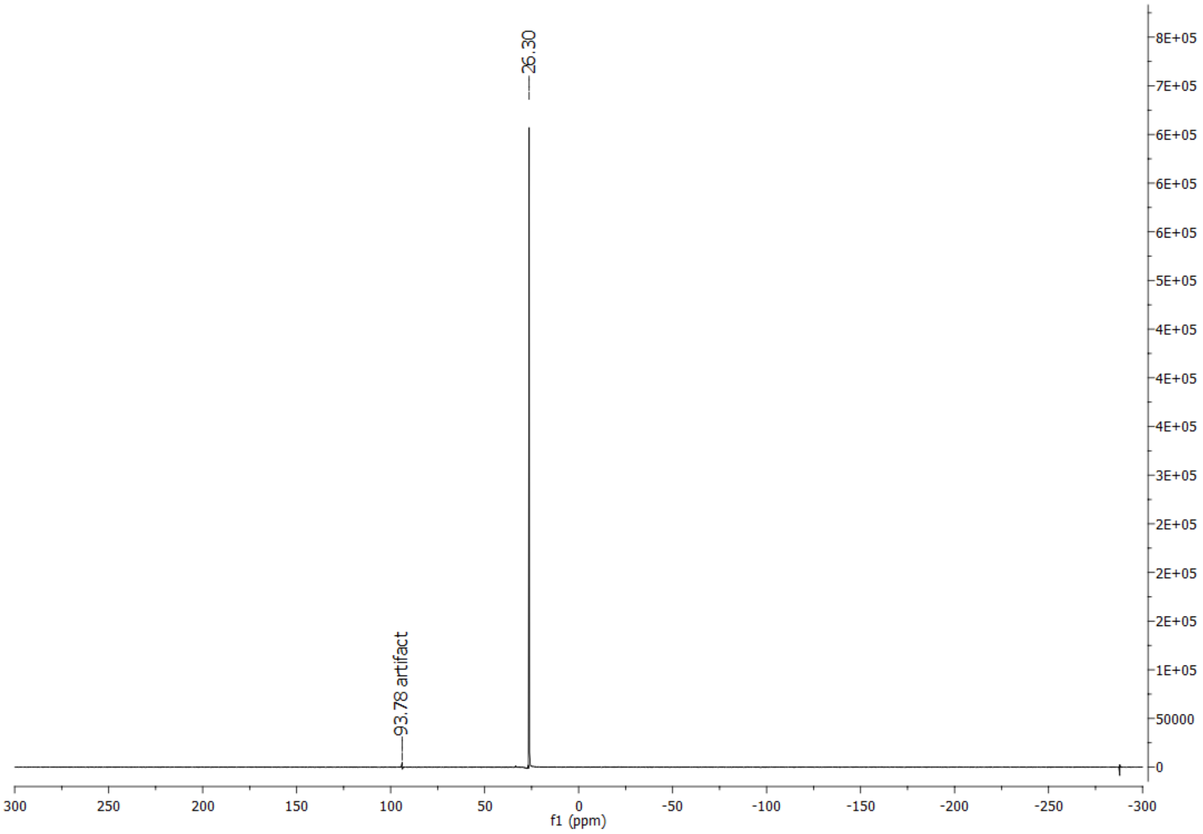

Figure S33. $^{31}P\{^{1}H\}$-NMR (121 MHz, $CDCl_3$) spectrum of **1a**.

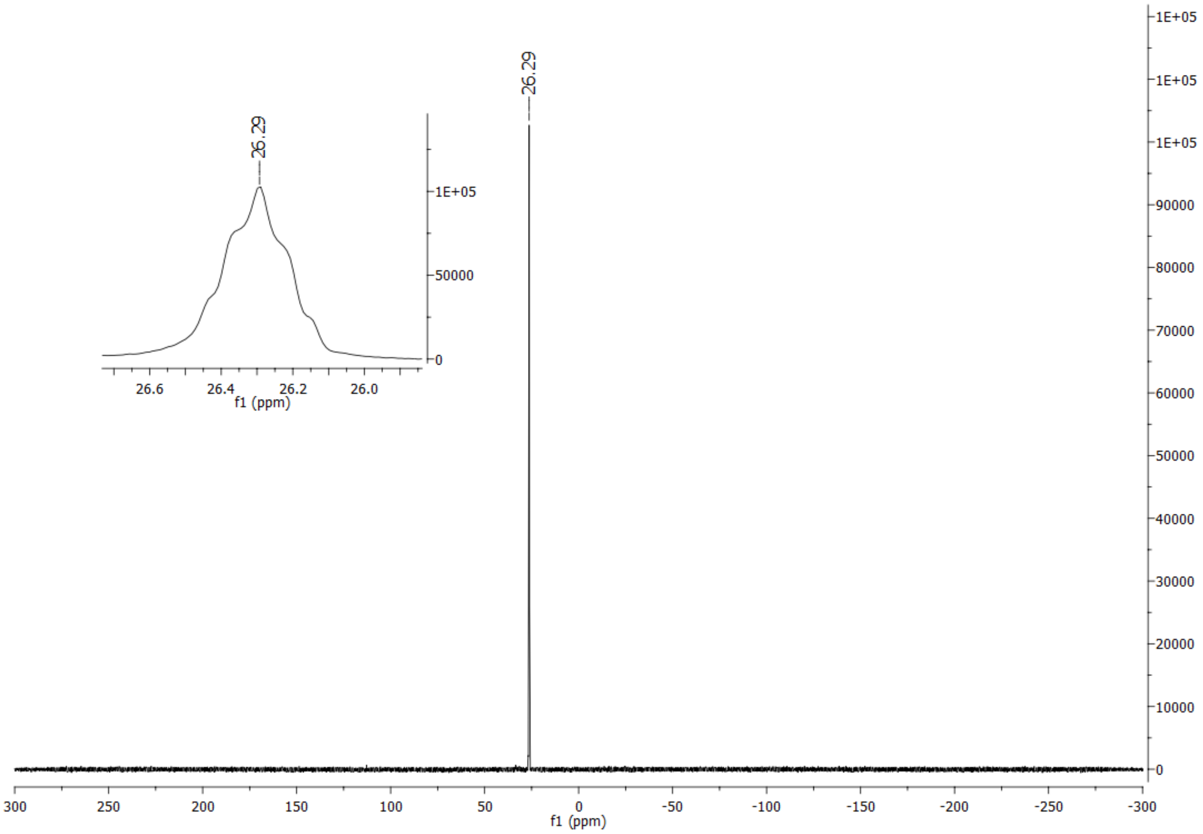

Figure S34. ^{31}P-NMR (121 MHz, $CDCl_3$) spectrum of **1a**.

Compound 1b

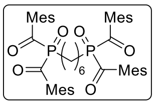

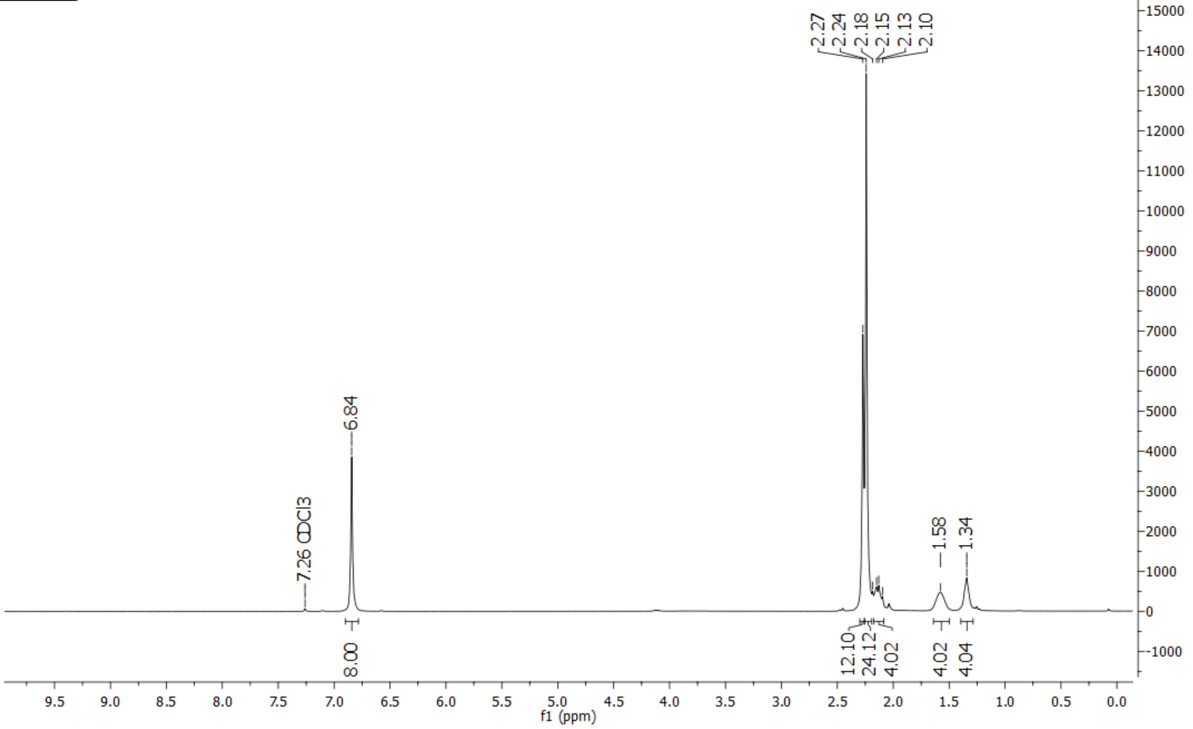

Figure S35. ^{1}H-NMR (300 MHz, $CDCl_3$) spectrum of **1b**.

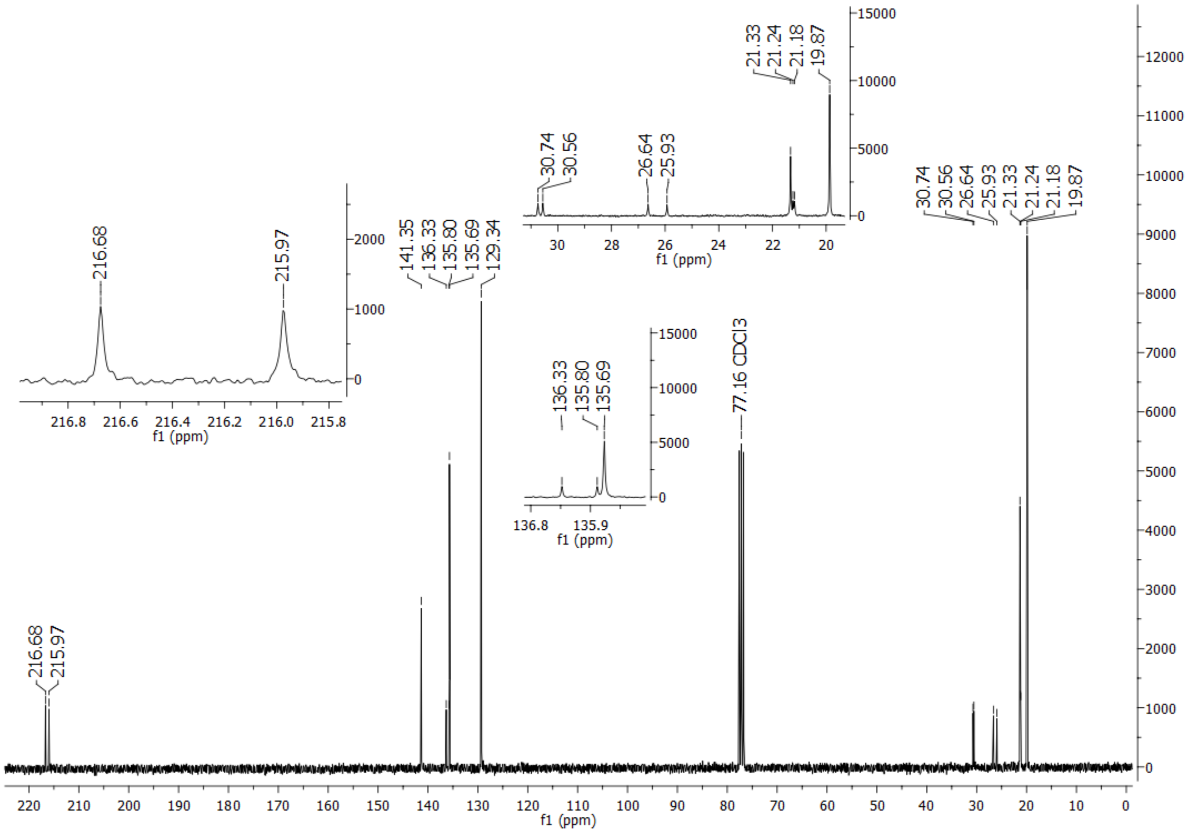

Figure S36. ^{13}C-NMR (75 MHz, $CDCl_3$) spectrum of **1b**.

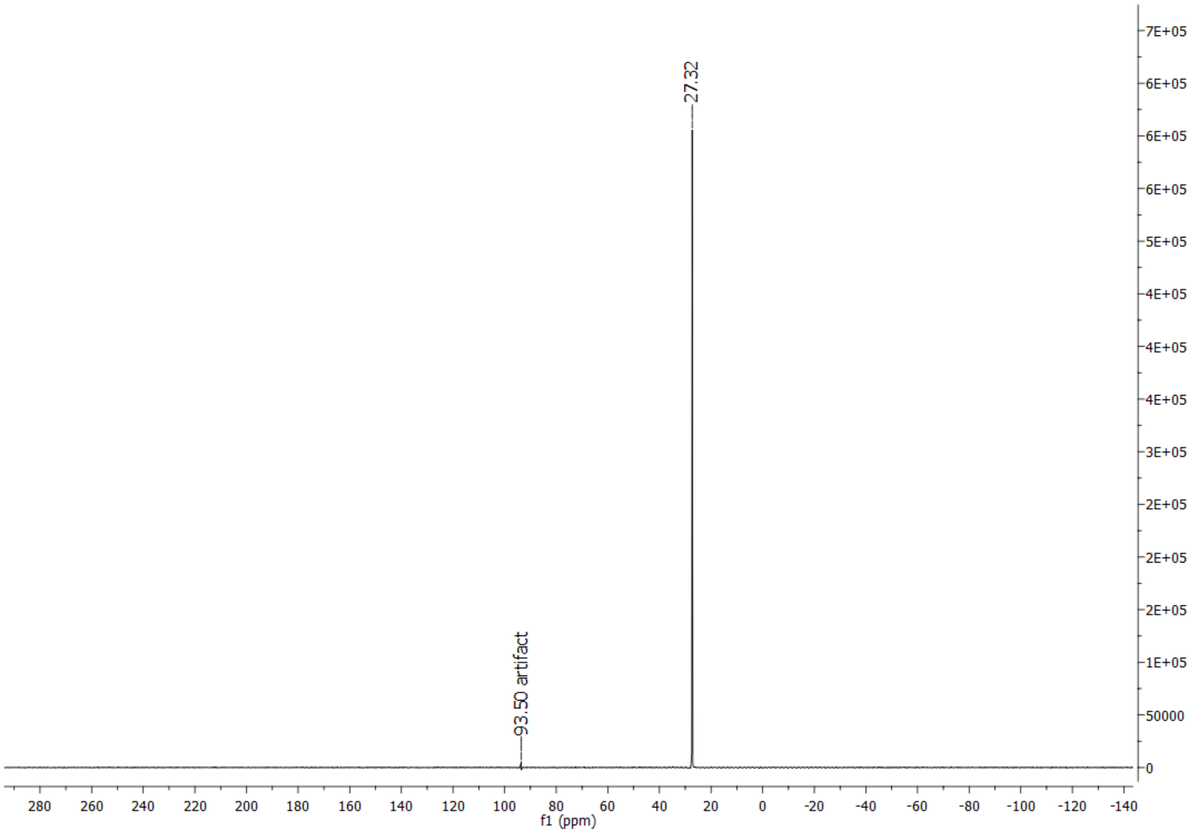

Figure S37. $^{31}P\{^{1}H\}$-NMR (121 MHz, $CDCl_3$) spectrum of **1b**.

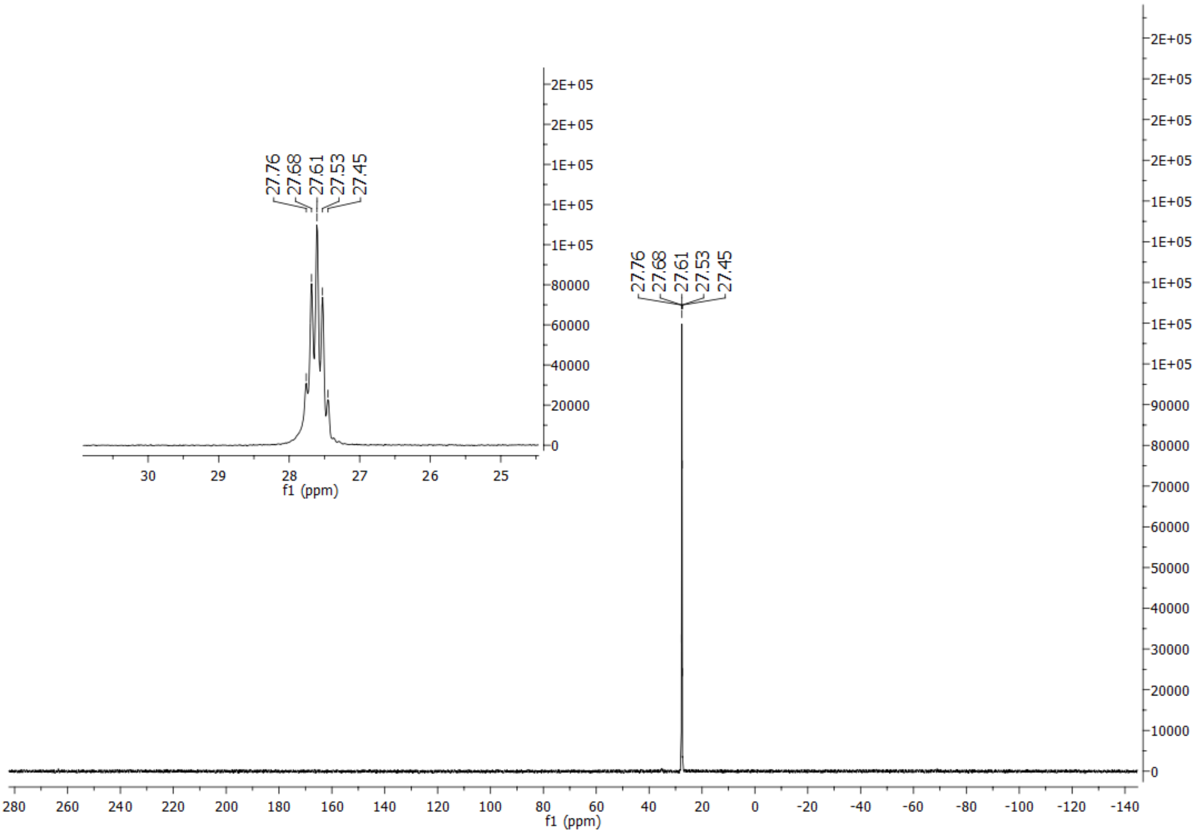

Figure S38. ^{31}P-NMR (121 MHz, $CDCl_3$) spectrum of **1b**.

Compound 1c

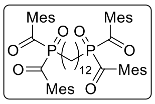

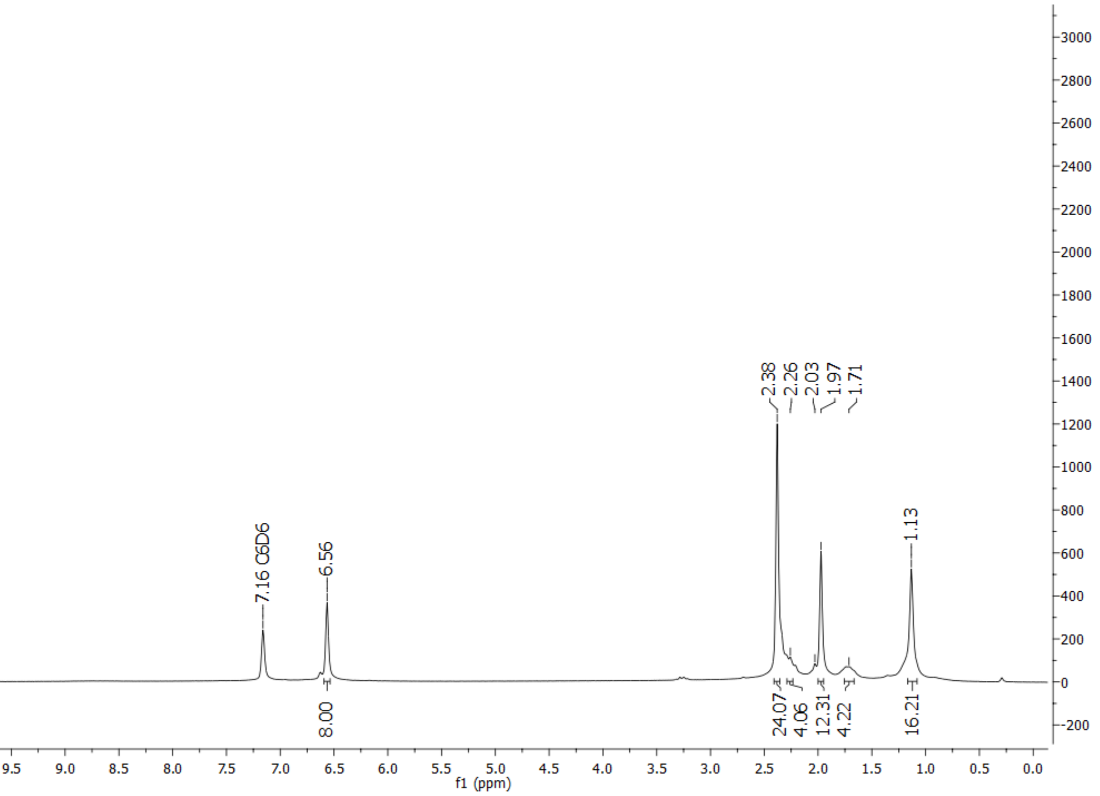

Figure S39. ^{1}H-NMR (300 MHz, C_6D_6) spectrum of **1c**.

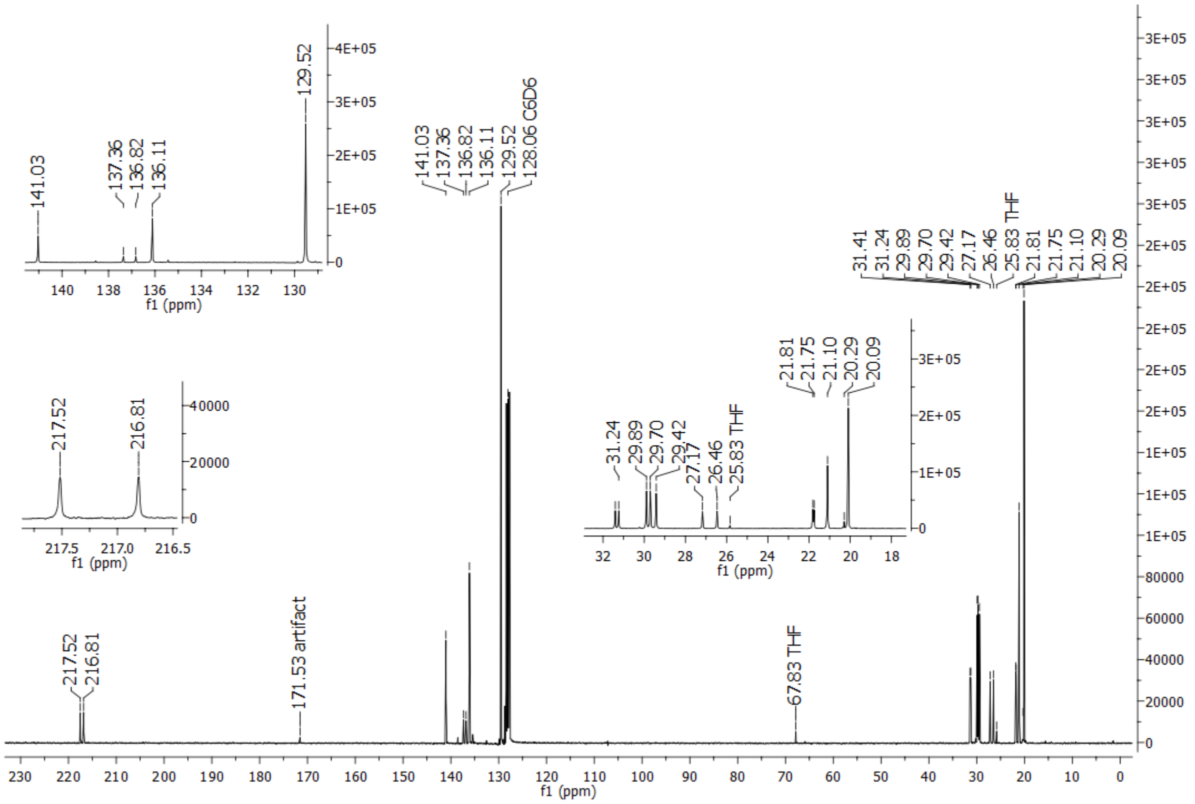

Figure S40. ^{13}C-NMR (75 MHz, C_6D_6) spectrum of **1c**

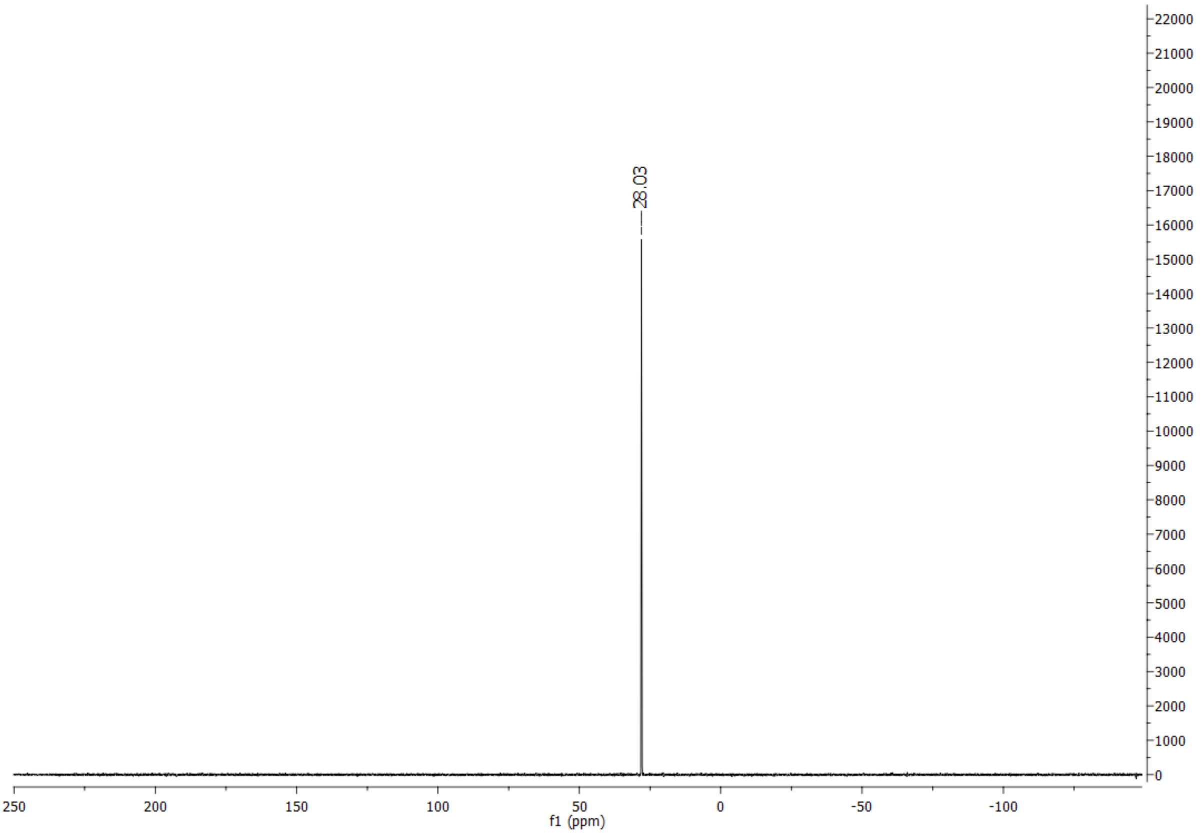

Figure S41. $^{31}P\{^{1}H\}$-NMR (121 MHz, C_6D_6) spectrum of **1c**

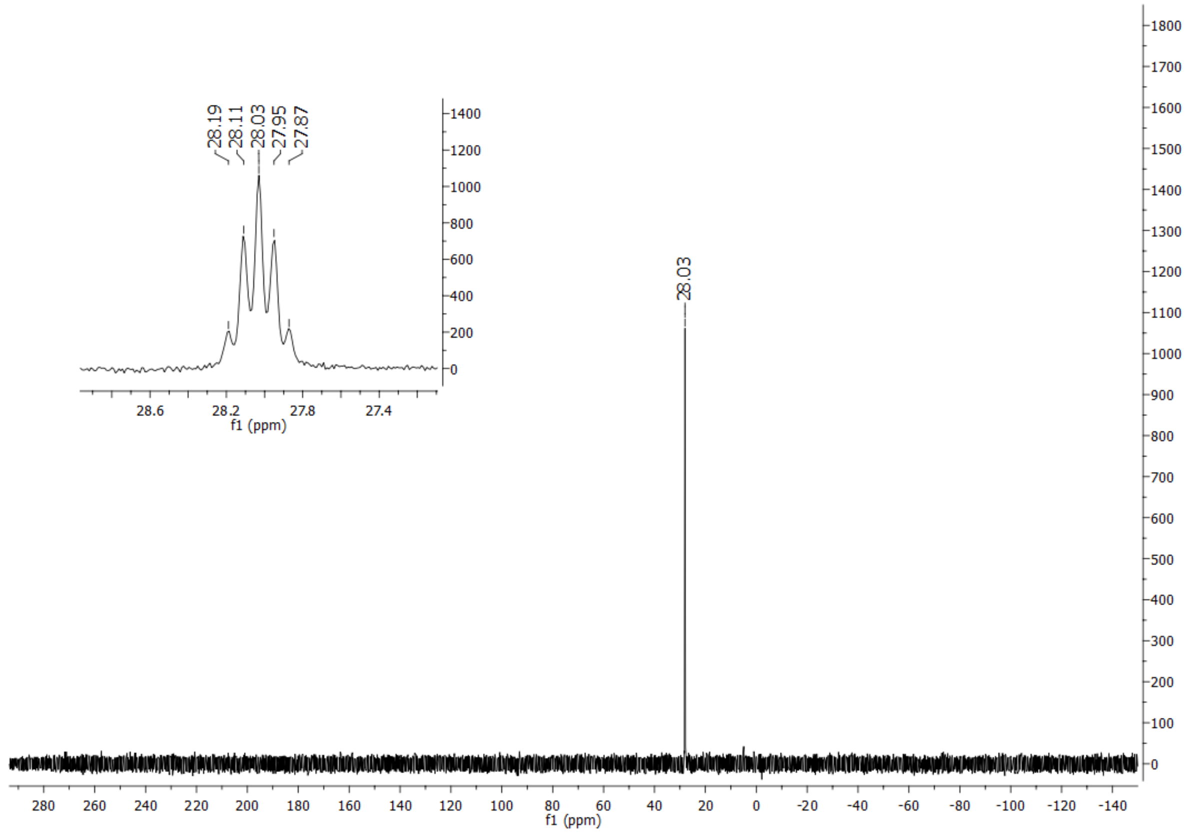

Figure S42. ^{31}P-NMR (121 MHz, C_6D_6) spectrum of **1c**

Compound 1d

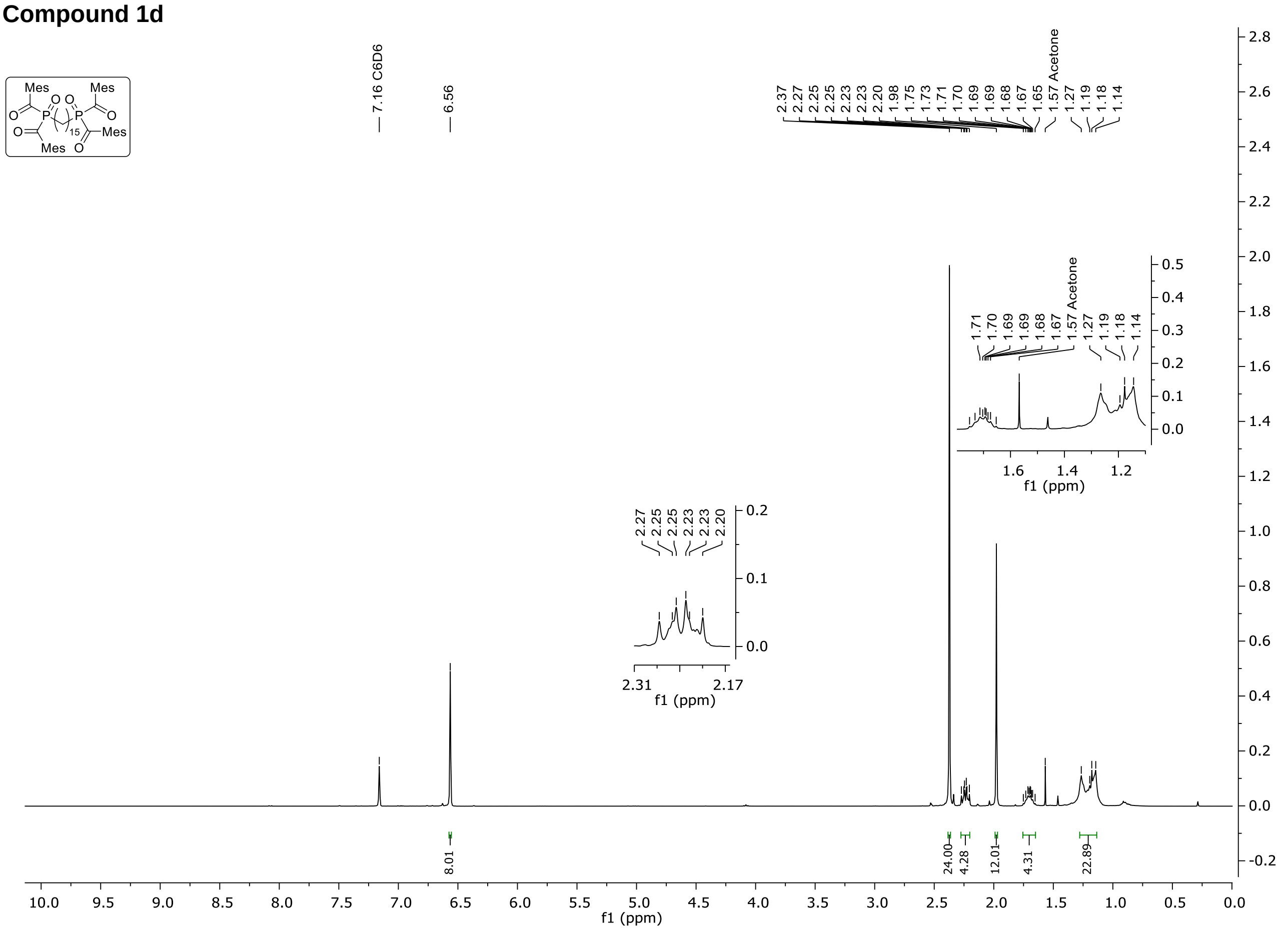

Figure S43. ^{1}H-NMR (400 MHz, C_6D_6) spectrum of **1d**

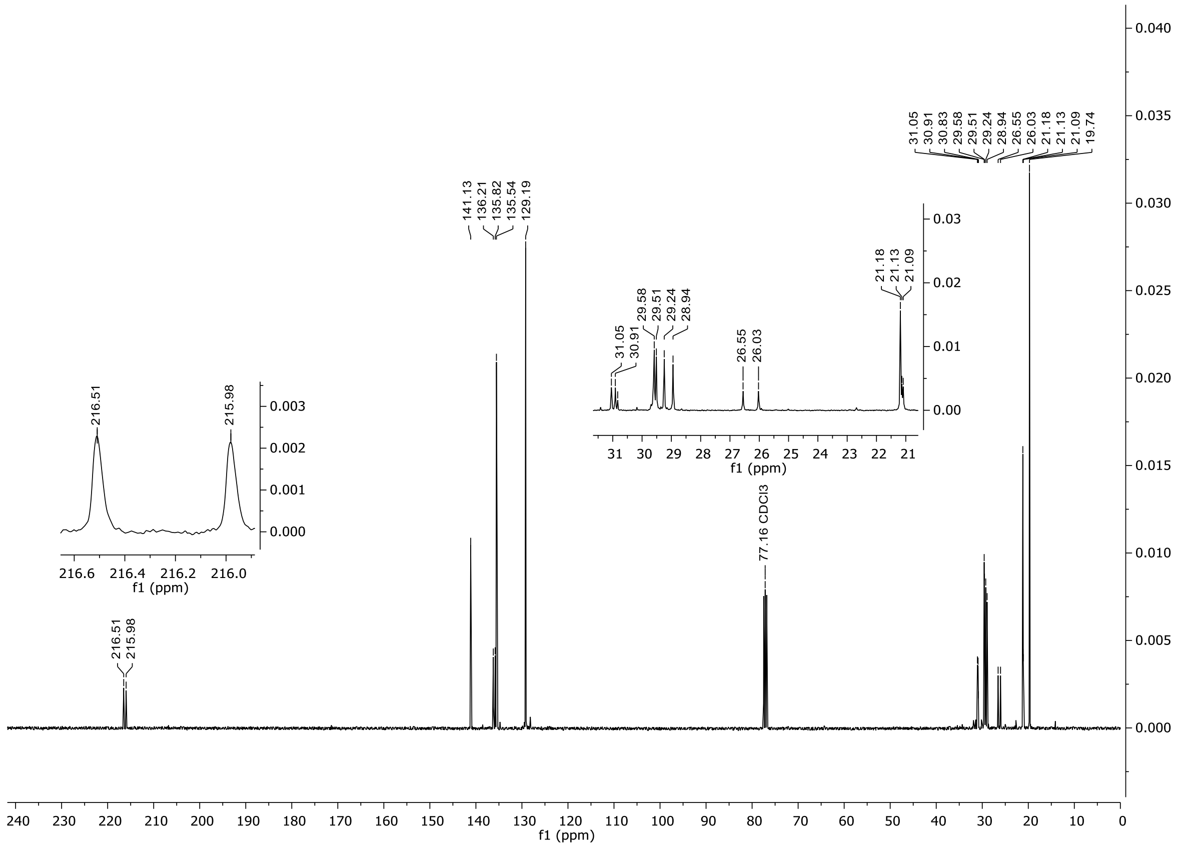

Figure S44. ^{13}C-NMR (100 MHz, $CDCl_3$) spectrum of **1d**

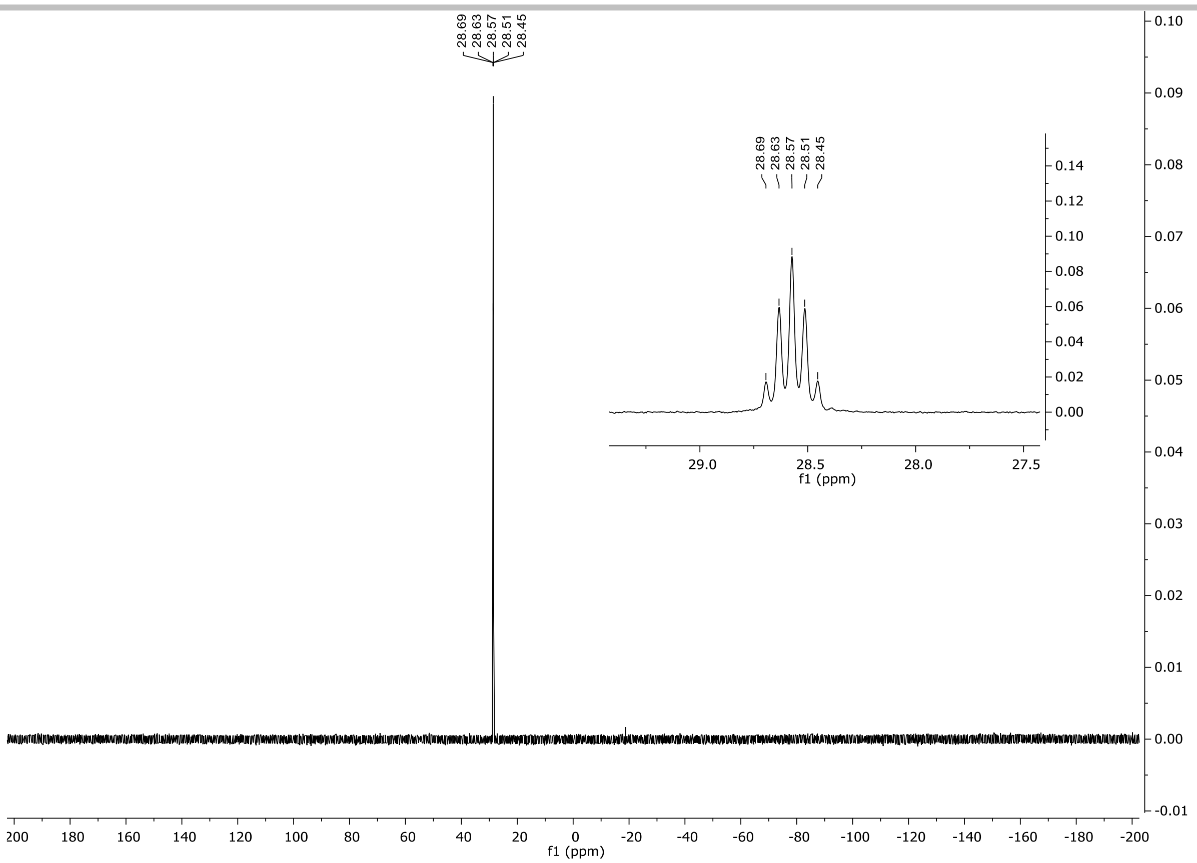

Figure S45. $^{31}P\{^{1}H\}$-NMR (162 MHz, C_6D_6) spectrum of **1d**

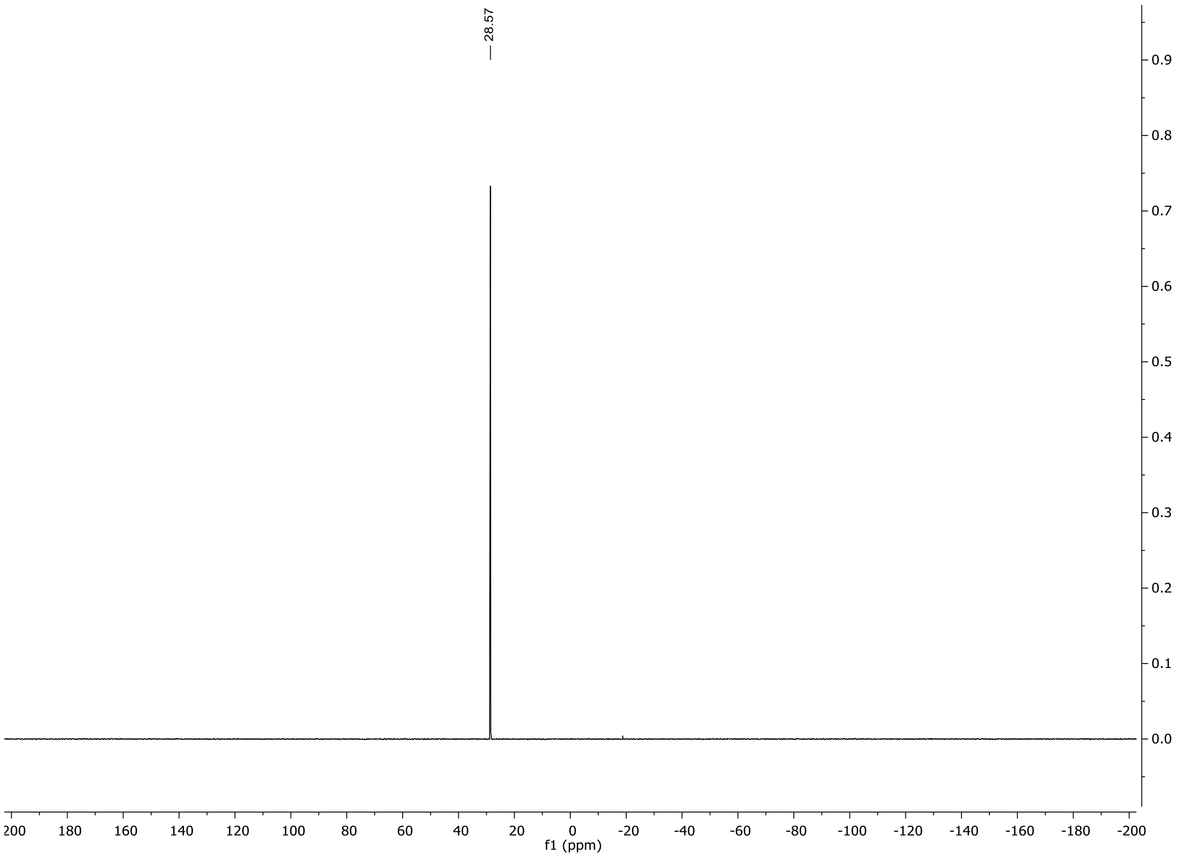

Figure S46. ^{31}P-NMR (162 MHz, C_6D_6) spectrum of **1d**

X-Ray Crystallography

General Considerations

All crystals suitable for single-crystal X-ray diffractometry were removed from a vial or Schlenk flask and immediately covered with a layer of silicone oil. A single crystal was selected, mounted on a glass rod on a copper pin, and placed in a cold N_2 stream provided by an Oxford Cryosystems cryometer (T=100 K). XRD data collection for compound **1a** and **1b** was performed on a Bruker APEX II diffractometer with use of an Incoatec microfocus sealed tube of Mo Kα radiation (λ = 0.71073 Å) from an IμS microsource and an APEX II CCD area detector. Data integration was carried out using SAINT.[9] Empirical absorption corrections were applied using SADABS or TWINABS.[9,10] The structures were solved with either the use of direct methods or the intrinsic phasing option in SHELXT and refined by the full-matrix least-squares procedures based on F^2 implemented in SHELXL[11] as implemented in SHELXLE.[12] The space group assignments and structural solutions were evaluated using PLATON.[13] Non-hydrogen atoms were refined anisotropically. All hydrogen atoms were either located in a difference map or in calculated positions corresponding to standard bond lengths and angles. CIF files were edited, validated and formatted with the programs encifer[14] and Olex2.[15] Structural plots and figures were generated with MERCURY.[16]

Crystallographic Data

Table S9 contains crystallographic data and details of measurements and refinements for compounds **1a**, and **1b**. Crystallographic data (excluding structure factors) have been deposited with the Cambridge Crystallographic Data Centre (CCDC) under the following numbers **1a**: **2532018**, **1b**: **2532017**.

Table S9. Crystallographic data of compounds **1a** and **1b**.

**Compound**	**1a CCDC: 2532018**	**1b CCDC:2532017**
Formula	$C_{44}H_{52}O_6P_2$	$C_{46}H_{56}O_6P_2$
M_r (g mol^{-1})	738.80	766.85
a (Å)	8.8313(4)	8.2682(4)
b (Å)	9.4049(4)	11.9013(6)
c (Å)	12.5226(5)	12.4910(6)
α (°)	76.124(2)	64.361(3)
β (°)	73.553(2)	73.193(3)
γ (°)	77.621(2)	71.115(3)
V (Å3)	956.37(7)	1032.33(9)
Z	1	2
Crystal size (mm)	0.25 × 0.18 × 0.10	0.16 × 0.13 × 0.10
Crystal habit	block, yellow	block, yellow
Crystal system	triclinic	triclinic
Space group	*P*-1	*P*-1
d_{calc} (g cm^{-3})	1.283	1.233
μ (mm^{-1})	0.16	0.15
T (K)	100	100
2θ range (°)	4.5–60.2	3.9-56.0
F (000)	394	410
T_{min}, T_{max}	0.480, 0.746	0.583, 0.746
R_{int}	0.090	0.122
No. of measured, independent and observed [I > 2σ(I)] reflections	55469, 5609, 4302	77260, 4971, 3788
independent reflections	5609	4971
No. of parameters, restraints	241, 0	250, 0
Δ›max, Δ›min (e Å^{-3})	0.61, -0.37	0.42, -0.47
R1, wR2 (all data)	R_1 = 0.0697	R_1 = 0.0689
	wR_2 = 0.1252	wR_2 = 0.1135
R1, wR2 (>2σ)	R_1 = 0.0470	R_1 = 0.0445
	wR_2 = 0.1128	wR_2 = 0.1032

Leaching Experiments

For the leaching experiments, 1,6-hexanediol diacrylate (HDDA) and a blend of 70 wt% urethane dimethacrylate (UDMA) with 30 wt% triethylene glycol dimethacrylate (TEGDMA) was used. Each resin was formulated with 2 wt% of the initiators **1a**–**d**. The resins were cured and then immersed in acetonitrile for 48 hours. The acetonitrile was subsequently filtered through a syringe filter to eliminate particles and other impurities. Inductively coupled plasma mass spectrometry (ICP-MS) was used to determine the amount of phosphorus in the solvent.

For ICP-MS analyses, 100 µL of a 100 ng/g indium solution were added to each sample as an internal standard, and the samples were filled up to a volume of approximately 20 mL with 1 % m/v HNO_3 solution. Measurements were performed using an Agilent 8800 ICP-MS/MS (Agilent Technologies, Santa Clara, USA), operated in MS/MS mode using O_2 as a reaction gas in order to remove polyatomic interferences. Quantification was carried out using aqueous phosphorus standard solutions (Merck KGaA, Darmstadt, Germany) for external calibration.

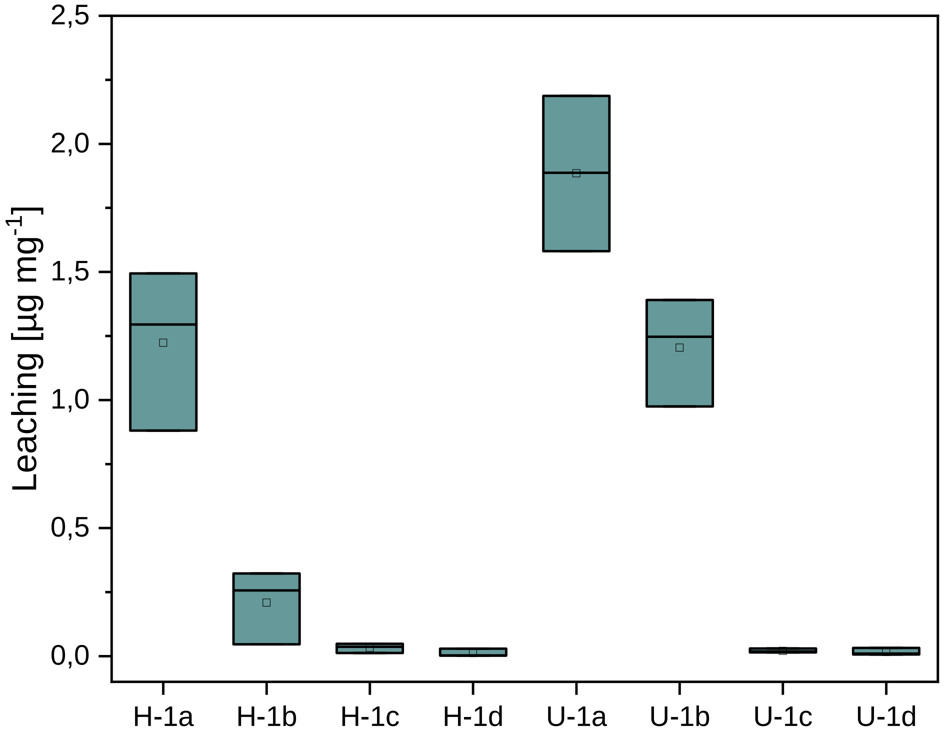

Figure 47. Boxplot of the concentration of phosphorous in the individual extracts of the migration analysis of photoinitiators **1a-d** in 1,6-Hexandioldiacrylate (H-**1a**-**1d**) and a mixture of 70 wt% Urethane dimethacrylate and 30 wt% Triethylenglycol dimethacrylate (U-**1a**-**1d**).

References

[1] A. B. Pangborn, M. A. Giardello, R. H. Grubbs, R. K. Rosen, F. J. Timmers, Organometallics 1996, 15, 1518.
[2] S. Grimme, J. G. Brandenburg, C. Bannwarth, A. Hansen, The Journal of chemical physics 2015, 143, 54107.
[3] F. Neese, F. Wennmohs, U. Becker, C. Riplinger, The Journal of chemical physics 2020, 152, 224108.
[4] V. Barone, M. Cossi, J. Phys. Chem. A 1998, 102, 1995.
[5] T. Petrenko, F. Neese, The Journal of chemical physics 2007, 127, 164319.
[6] M. D. Hanwell, D. E. Curtis, D. C. Lonie, T. Vandermeersch, E. Zurek, G. R. Hutchison, Journal of cheminformatics 2012, 4, 17.
[7] E. Stadler, A. Eibel, D. Fast, H. Freißmuth, C. Holly, M. Wiech, M. Moszer, G. Gescheidt, Photochemical & photobiological sciences 2018, 17, 660.
[8] T. Lehóczki, É. Józsa, K. Ősz, Journal of Photochemistry and Photobiology A: Chemistry 2013, 251, 63.
[9] Bruker APEX2 and SAINT, Bruker AXS Inc.: Madison, Wisconsin, USA, 2012.
[10] a) R. H. Blessing, Acta crystallographica. Section A, Foundations of crystallography 1995, 51 (Pt 1), 33; b) G. M. Sheldrick, SADABS, Univ. Göttingen, Ger.
[11] a) G. M. Sheldrick, Acta Crystallogr A Found Crystallogr 1990, 46, 467; b) G. M. Sheldrick, Acta crystallographica. Section A, Foundations of crystallography 2008, 64, 112; c) G. M. Sheldrick, Acta crystallographica. Section A, Foundations and advances 2015, 71, 3.
[12] C. B. Hübschle, G. M. Sheldrick, B. Dittrich, J Appl Crystallogr 2011, 44, 1281.
[13] a) A. L. Spek, J Appl Crystallogr 2003, 36, 7; b) A. L. Spek, Acta crystallographica. Section D, Biological crystallography 2009, 65, 148.
[14] F. H. Allen, O. Johnson, G. P. Shields, B. R. Smith, M. Towler, J Appl Crystallogr 2004, 37, 335.
[15] O. V. Dolomanov, L. J. Bourhis, R. J. Gildea, J. A. K. Howard, H. Puschmann, J Appl Crystallogr 2009, 42, 339.
[16] C. F. Macrae, I. Sovago, S. J. Cottrell, P. T. A. Galek, P. McCabe, E. Pidcock, M. Platings, G. P. Shields, J. S. Stevens, M. Towler et al., J Appl Crystallogr 2020, 53, 226.
